# Conformationally Locked Carbocyclic Nucleosides Built on a 4′-Hydroxymethyl-3′-hydroxybicyclo[4.1.0]heptane Template. Stereoselective Synthesis and Antiviral Activity

**DOI:** 10.1021/acs.joc.2c01661

**Published:** 2022-10-27

**Authors:** Sergio Jurado, Ona Illa, Angel Álvarez-Larena, Christophe Pannecouque, Félix Busqué, Ramon Alibés

**Affiliations:** † Departament de Química, Universitat Autònoma de Barcelona, Bellaterra, Barcelona 08193, Spain; ‡ Servei de Difracció de Raigs X, Universitat Autònoma de Barcelona, Bellaterra, Barcelona 08193, Spain; § Department of Microbiology and Immunology, Laboratory of Virology and Chemotherapy, Rega Institute for Medical Research, KU Leuven, Herestraat 49, Leuven B-3000, Belgium

## Abstract

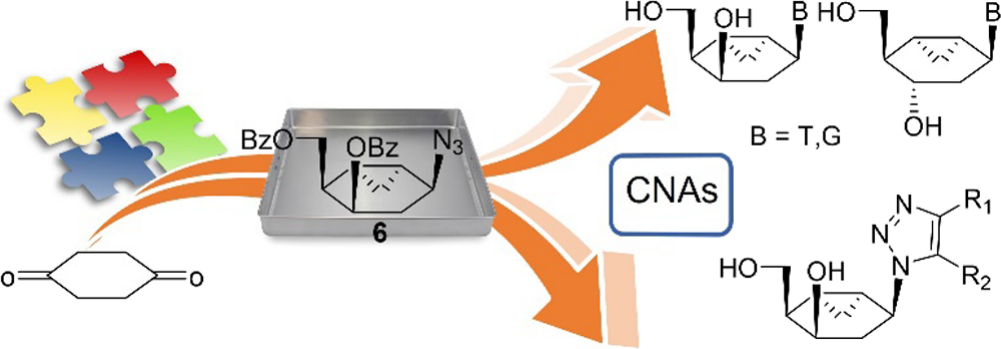

Two new families of enantiomerically pure carbocyclic nucleoside analogues based on a cyclohexane moiety with five chiral centers and a fused cyclopropyl ring have been synthesized. A highly regio- and stereoselective synthetic approach for the modular construction of the functionalized bicyclo[4.1.0]heptyl azide intermediate **6** has been established. Key steps to achieve this asymmetric synthesis involved highly diastereoselective allylic oxidation and hydroboration reactions. The first family of compounds, **1a**,**b** and **2**, presents different natural nucleobases, whereas the second one **3a**–**e** bears functionalized 1,2,3-triazoles. These derivatives have been tested as antiviral agents, and compound **3d** has shown to display moderate activity against coxsackie B4 virus.

## Introduction

Nucleoside analogues (NAs) are an important class of small molecule-based antivirals, which mainly act by interfering with the metabolism and function of natural nucleosides. These prodrugs constitute the backbone for the treatment of chronic infections provoked by HIV, herpes viruses, and hepatitis B or C viruses.^1−3^ However, only 10 human viral pathogens can be treated with antiviral drugs.^4,5^ Moreover, the SARS-CoV-2 pandemic has emphasized the need for compounds that can respond to future outbreaks provoked by other emerging viruses. Thus, the fight against COVID has renewed the researchers’ interest in NAs.^6,7^ In the search for novel antiviral NAs, natural nucleosides have been submitted to several chemical modifications. Among them, those resulting in changes on the sugar moiety and/or on the heterocyclic bases of endogenous nucleosides have led to the development of a series of compounds displaying a wide range of antiviral activity.^8^

**Figure 1:**
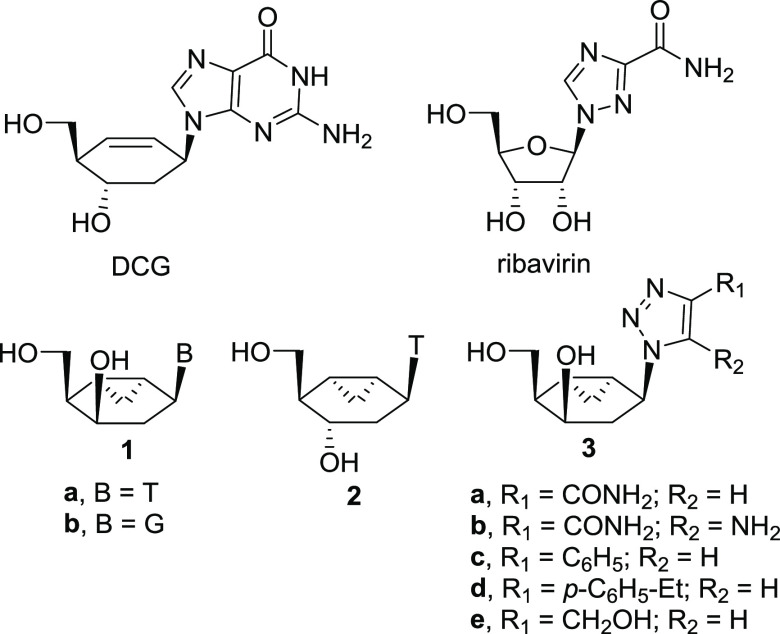
Selected biologically active nucleoside analogues: cyclohexenyl-G (DCG) and ribavirin, and targeted carbocyclic analogues **1a**,**b**, **2**, and **3a**–**e**.

Carbocyclic NAs (CNAs) are structurally related to ribonucleosides in which a carbocycle is in the place of the sugar moiety.^9,10^ CNAs are recognized by the same enzymes as natural nucleosides and are more stable toward hydrolysis by phosphorylases displaying enhanced biostability. This family of CNAs includes, among others, the cyclopentene derivatives abacavir and entecavir and the six-membered counterpart cyclohexenyl G (DCG, Figure 1).^11^ In particular, cyclohexenyl nucleosides are an interesting class of antiviral compounds, wherein the presence of a double bond instead of the oxygen atom of the furanose ring produces a comparable annular flexibility to that of the parent nucleoside.^12^ Interestingly, antiviral activity against some herpes viruses (HSV-1, HSV-2, VZV, CMV) was found to be the same for both enantiomers of DCG.^13^ The conformational restriction of a nucleoside has often been used to enhance selectivity and/or potency against the target enzymes.^14,15^ Reducing the overall number of flexible conformations could favor the adoption of a bioactive conformation and, therefore, molecular recognition by the target enzyme. Some conformationally locked carbocyclic analogues by a fused cyclopropane have been described to present similar conformations than those of the natural counterparts and have shown improved enzyme recognition.^16,17^

On the other hand, modification of the natural nucleobases, including five-membered heterocyclic nucleobases such as a triazole ring, has resulted in the discovery of a series of compounds with a broad spectrum of activities against diverse viruses.^2,5,8^ A remarkable example of this group of compounds is ribavirin, which is a 1,2,4-triazole nucleoside drug with a wide range of antiviral activities.^18^ 1,2,3-Triazole derivatives have also been reported as antimicrobial,^19^ anticancer,^20^ and potential antiviral^21,22^ agents even in the field of CNAs.^23,24^

Over the past years, our research group has worked on the development of new routes for the enantioselective preparation of cyclohexane NAs as prodrug candidates.^25^ We recently described the design and synthesis of a new class of CNAs based on a bicyclo[4.1.0]heptane scaffold.^26^ As part of our continuing research program to identify bioactive NAs, we were interested in developing novel cyclohexenyl-G derivatives bearing an appended cyclopropyl ring **1a**,**b** and unnatural bases, such as a triazole heterocycle **3a**–**e** (Figure 1). It was expected that the fusion to a cyclopropane would impart a significant rigidity to the resulting nucleosides similar to that of the cyclohexene moiety but should be less prone to hydrolytic processes and have more lipophilicity. Limiting flexible conformation with a fused cyclopropane is a useful strategy to increase the potency and selectivity of a nucleoside.^16,17,27^

Herein, we present a full account of the stereoselective synthesis and the antiviral testing of several enantiomerically pure six-membered CNAs with five chiral centers starting from 1,4-cyclohexanedione.

**Scheme 1:**
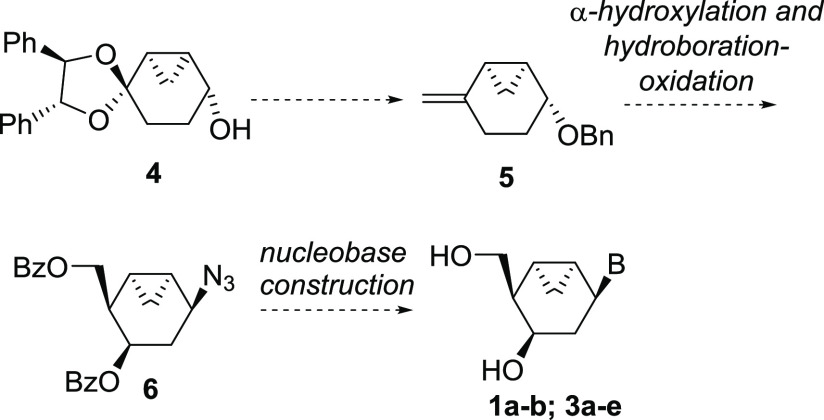
Synthetic Approach Toward CNAs **1a**,**b** and **3a**–**e**

The known enantiomerically pure bicyclo[4.1.0]heptane alcohol **4** was envisaged as a suitable starting material (Scheme 1).^26^ The synthetic plan contemplated the preparation of the pivotal azide **6** from which thymine and guanine nucleobases would be stepwise constructed and the substituted 1,2,3-triazoles would be assembled via cycloaddition. A major challenge of the synthetic approach would be the regio- and stereoselective allylic hydroxylation and olefin hydration on compound **5**.

## Results and Discussion

**Scheme 2:**
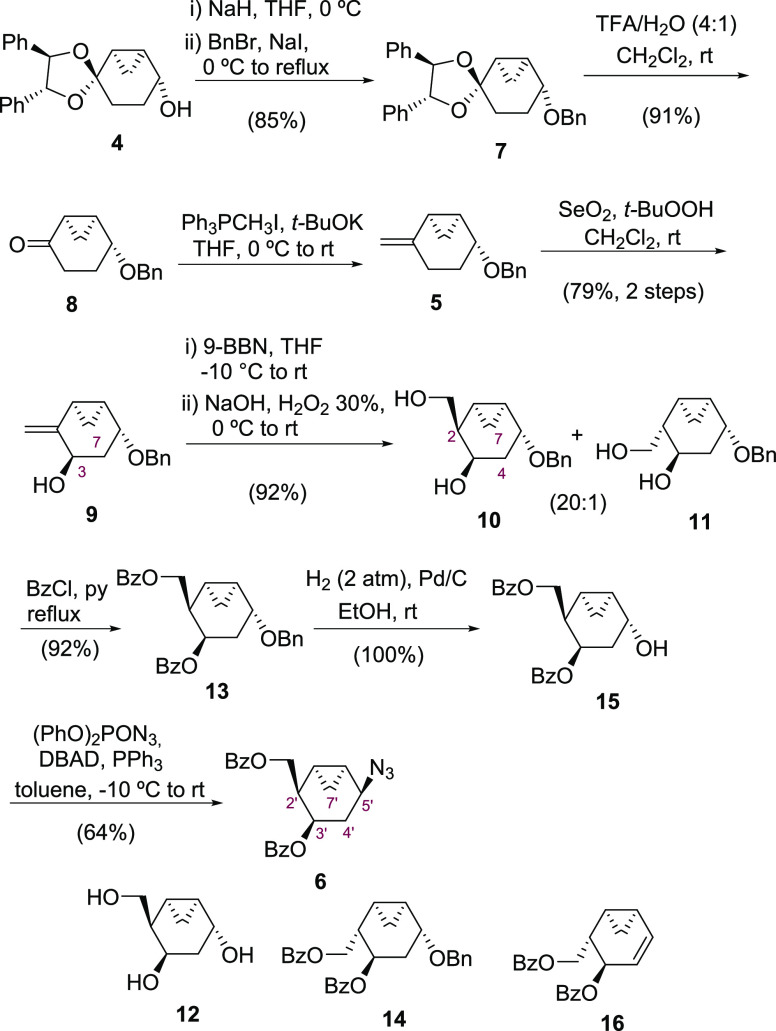
Synthesis of Key Azide **6**

The synthesis started with alcohol **4** previously prepared in 80% yield in four steps from commercially available 1,4-cyclohexanedione on a multigram scale by a methodology devised in our group (Scheme 2).^26^ Benzyl etherification of the alcohol under standard conditions, followed by ketal removal under acidic conditions (4:1 mixture of TFA/H_2_O), afforded ketone **8** in 77% for the two steps. Reaction of ketone **8** with freshly prepared ylide generated from methyltriphenylphosphonium iodide (Ph_3_PCH_3_I) and *t*-BuOK afforded the corresponding terminal alkene **5**, which was submitted without further purification to allylic oxidation. Accordingly, compound **5** was treated with catalytic SeO_2_ and stoichiometric *tert*-butyl hydroperoxide^28,29^ to furnish the corresponding allylic alcohol **9** as a single diastereoisomer in 79% yield from ketone **8**. The excellent regio- and stereoselectivity furnished by this oxidation reaction can be rationalized by the generally accepted mechanism that implies two consecutive pericyclic reactions, an ene reaction followed by a [2,3]-sigmatropic rearrangement.^30^ The latter process occurs through the less hindered face delivering the anti-adduct. The anti-relative configuration was determined by a 2D NOESY experiment, where cross peaks between H-3 and H-7endo of the cyclopropane revealed the *cis* relative disposition between H-3 and cyclopropane.

The next step of our synthetic plan was the stereoselective hydroxymethyl installation by a hydroboration-oxidation procedure. In a first attempt, hydroboration of **9** using BH_3_·THF followed by alkaline hydrogen peroxide workup furnished a 5:1 mixture of diols **10** and **11** in 90% yield. However, when the reaction was carried out using 9-BBN, a 20:1 mixture of diols **10** and **11** was obtained in good yield (92%). The origin of the remarkable stereoselectivity observed might be rationalized by an initial coordination of the borane reagent (9-BBN) to the secondary hydroxyl group. The resulting borinate ester shields the top face, favoring the hydroboration of the olefinic functionality from the bottom face by a second borane reagent. Thus, the mainly formed hydroxymethyl group was found at the top face of the ring.^31^ The hydrolysis of the borate intermediate in the oxidation step under basic conditions delivers mainly alcohol **10**.^32,33^ Therefore, in this reaction, more than two equivalents of the borane reagent were employed.

A pure fraction of **10** was obtained after several purifications by column chromatography from the mixture of diols. The configuration of the new stereogenic center was determined by the 2D NOESY experiment, which displays cross peaks between H-2, H-7endo, and H-4ax disclosing the *cis* relationship between these three protons. The relative configuration was further confirmed by the cleavage of the benzyl protecting group to deliver the corresponding triol **12**, which provided adequate crystals for X-ray diffraction analysis.

Next, reaction of the diastereomeric mixture of diols **10** and **11** with an excess of benzoyl chloride furnished a chromatographically separable mixture of **13** and **14** in 92 and 5% yields, respectively. Hydrogenation of the benzyl protecting group in **13** under palladium catalysis furnished alcohol **15** in quantitative yield. Finally, alcohol **15** was transformed into azide **6** in 64% yield following a Mitsunobu protocol^34^ using diphenylphosphoryl azide (DPPA) in the presence of di-*tert*-butyl azodicarboxylate (DBAD) and triphenylphosphine (PPh_3_). Along with the desired azide, the elimination product **16** was also obtained in low yield (5%). The configuration of C-5′ was determined by the presence of strong cross peaks between H-5′ and H-7′endo and H-4′ax and between H-5′ and H-2′ and H-3′ in the NOESY spectrum and was further confirmed by X-ray diffraction analysis. Therefore, the stereocontrolled preparation of the chiral cyclohexane scaffold bearing the five stereogenic centers has been successfully accomplished starting from 1,4-cyclohexanedione.

**Scheme 3:**
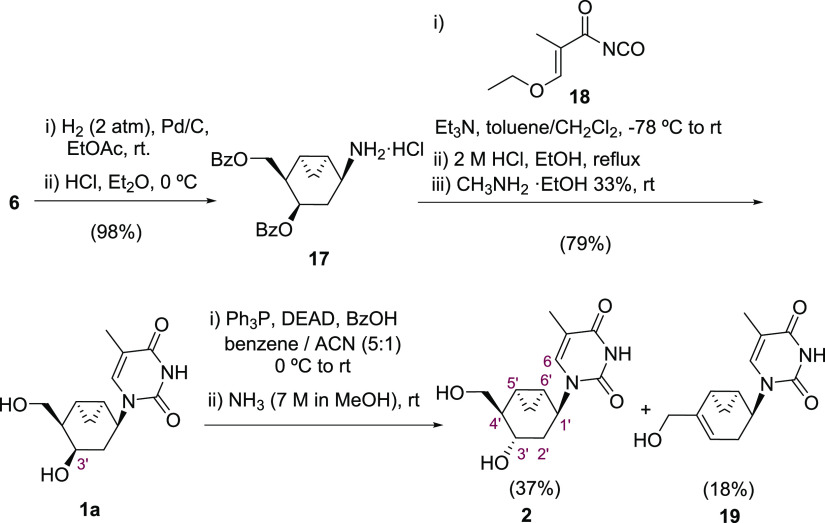
Synthesis of Bicyclic Nucleoside Analogues **1a**, **2a**, and **19**

With **6** in hand, we next examined the stepwise construction of the thymine and guanine nucleobases (Scheme 3). First, catalytic hydrogenation delivered quantitatively the corresponding primary amine, which was isolated as the ammonium chloride salt **17**. Then, the primary amine was treated with the freshly prepared acyl isocyanate **18** at low temperature in basic media^35,36^ and the formed acryloyl urea was successively heated at reflux in ethanolic acid and treated with 7 M methanolic ammonia solution to furnish the pyrimidine NA **1a** in 79% yield. This compound provided an X-ray crystal structure that confirmed the stereostructure. To prepare epimeric analogue **2**, the unprotected NA **1a** was subjected to standard Mitsunobu conditions using benzoic acid followed by subsequent alcohol deprotection with an ammonia solution in methanol.^17^ These conditions provided the epimeric NA **2** in 37% yield along with alkene **19** in 18% yield. The inversion of the configuration at C-3′ was assessed by a NOESY experiment, which exhibited a strong cross peak between H-3′ and H-6 of the thymine base.^37^

**Scheme 4:**
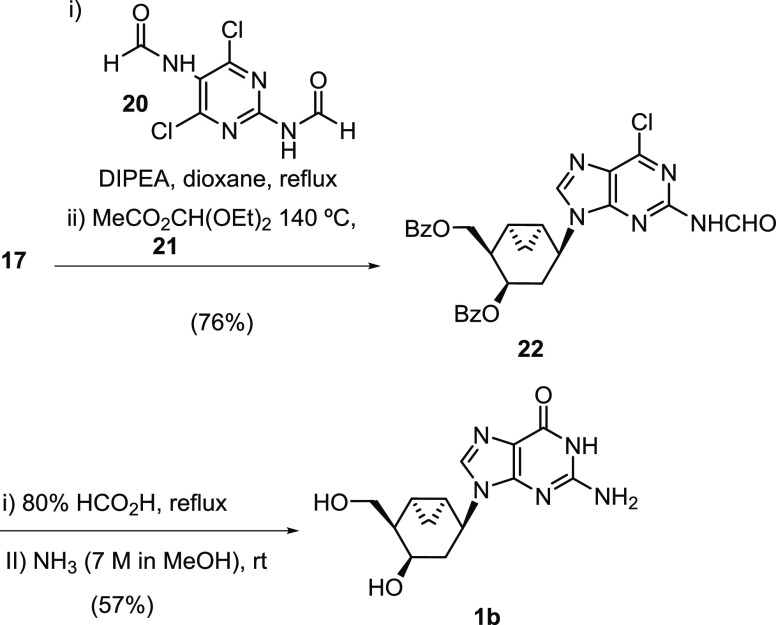
Synthesis of Bicyclic Guanine Analogue **1b**

The guanine base was also assembled from carbocyclic amine **17** (Scheme 4). The purine ring was efficiently built (76%) through a two-step procedure by reacting **17** first with the diformyl derivative **20** at the reflux temperature and then immediately cyclizing the transiently isolated intermediate in the presence of diethoxymethyl acetate, **21**, at 140 °C to give compound **22**.^38,39^ Acidic hydrolysis under reflux followed by reaction with a 7 M solution of MeNH_2_ in EtOH afforded the desired NA **1b** in 57% overall yield. Unexpectedly, all attempts to epimerize C-3′ of **1b** using standard Mitsunobu conditions were unsuccessful, resulting in degradation products only.

**Scheme 5:**
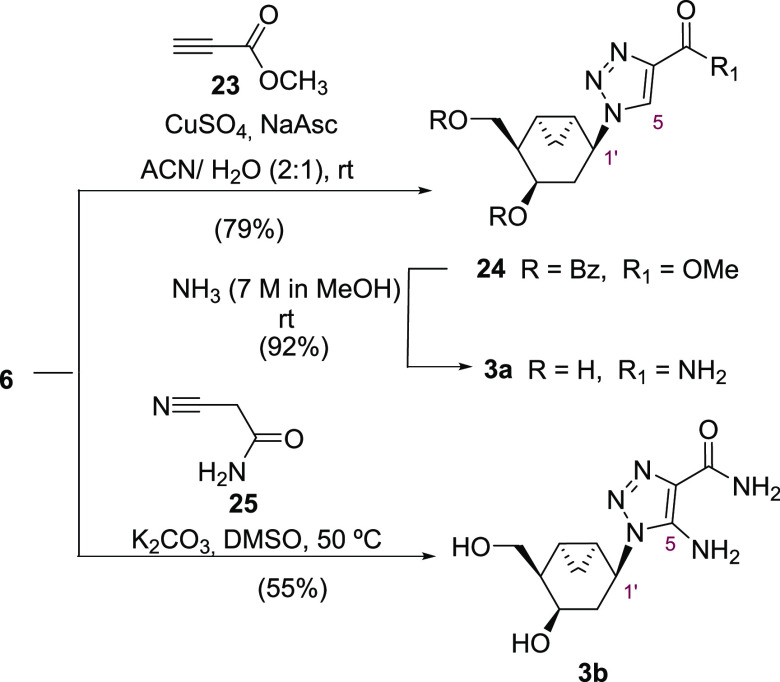
Synthesis of Bicyclic 1,2,3-Triazolo Carbanucleosides **3a** and **3b**

**Scheme 6:**
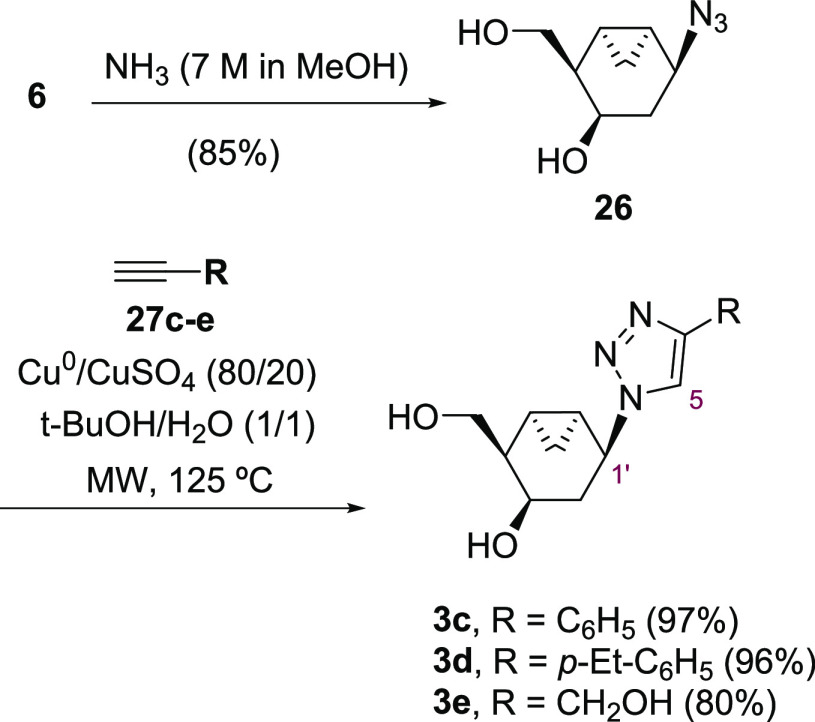
Synthesis of Bicyclic 1,2,3-Triazolo Carbanucleosides **3c**–**3e**

Our next effort focused on exploiting the common intermediate **6** to build a substituted 1,2,3-triazolo moiety (Schemes 5 and 6). First, the preparation of 1,2,3-triazole-4-carboxamide ribavirin analogues was attempted via copper(I)-catalyzed azide alkyne cycloaddition (CuAAC) using standard conditions.^40^ Thus, azide **6** was reacted with methyl propiolate **23** in the presence of CuSO_4_·5H_2_O (10 mol %) and sodium ascorbate (20 mol %) in a 2:1 EtOH/H_2_O mixture overnight. Unfortunately, only starting material was recovered, as the reaction did not work. The same result was obtained using MeOH or *t-*BuOH. Alcohol deprotection to increase the solubility of **6** was precluded due to the known conjugated addition of alcohols on the methyl propiolate.^40^ After some experimentation, it was found that using a 2:1 mixture of ACN/H_2_O, the cycloaddition proceeded readily to deliver the 4-metylcarboxylate triazole **24** in 79% yield. The latter was further easily transformed into the 1,2,3-triazole-4-carboxamide carbanucleoside **3a** by treatment with an ammonia solution in methanol (Scheme 5). Next, to explore more structural diversity, efforts were focused on the synthesis of a 5-amino-4-carboxamide-1,2,3-triazolo carbanucleoside **3b** related to acadesine^41^ using a known cycloaddition reaction under basic conditions.^42−44^ Accordingly, azide **6** was treated with 2-cyanoacetamide **25** in the presence of K_2_CO_3_ in DMSO at 50 °C to afford directly the 1,4,5-trisubstituted-triazolo deprotected carbanucleoside **3b** in moderate yield.

Finally, to expand the collection of NAs bearing triazole rings, the syntheses of various 4-substituted triazole compounds were undertaken starting from terminal alkynes **27c**–**e** via CuAAC (Scheme 6). First, to avoid the previously described solubility issues of **6**, the benzoyl protecting groups were removed using an ammonia solution in methanol providing the deprotected azide **26** in 85% yield. Then, **26** was reacted with alkynes **27c**–**e** using a Cu^0^/CuSO_4_ mixture in *t-*BuOH/H_2_O (1:1) media under microwave (MW) irradiation delivering the expected 4-substituted-1,2,3-triazole carbanucleosides **3c**–**e** in good yields in short reaction times.

The regioselectivity of the cycloaddition reactions (Schemes 5 and 6) was confirmed by HMBC experiments in compounds **3a**–**3e**, which show cross peaks between H-1′ of the bicyclo[4.1.0]heptane moiety and C-5 of the 1,2,3-triazole ring.

The antiviral activity against various viruses and the cytotoxicity of the synthesized compounds **1a**,**b**, **2**, **19**, and **3a**–**e** have been tested, as shown in the Supporting Information. While all the carbocyclic nucleosides did not display any significant antiviral activity, the 1,2,3-triazole analogue **3d** exhibited moderate antiviral activity [EC_50_ 9.4 μg/mL, selectivity index (SI) = 8.4] against coxsackie B4 virus. The coxsackie virus causes a great variety of diseases such as myocarditis^45^ or pericarditis,^46^ or aseptic meningitis,^47^ and is one of the main enteroviruses causing the *hand, foot, and mouth* disease in children under the age of 10.^48^ Moreover, coxsackie B4 virus has been related to the infection of β cells in patients with type 1 diabetes and that infection is associated with inflammation and functional impairment.^49^ Precedents of active NAs against coxsackie B4, using ribavirin derivatives, showed similar antiviral activity to our compounds.^50^ Remarkably, a seminal work using neplanocin-related CNAs described higher activities against coxsackie B4.^51^ Interestingly, another precedent using CNAs pointed out that the use of cyclopentenyl derivatives showed higher activity against coxsackie B4 than the corresponding saturated counterparts.^52^ Recently, other non-CNAs have been described with important antiviral activity toward the closely related coxsackie B3 virus.^53,54^ In one of these works, the use of 1,2,3-triazole-based non-natural nucleobases proved to be effective to improve the antiviral activity.^54^ However, in other cases, the use of non-natural nucleobases in linear CNAs has not led to significant antiviral activity against coxsackie B4 virus.^55,56^ Despite all these precedents, at present, there is no approved specific treatment for coxsackie virus infections. The preliminary *in vitro* antiviral activity of the novel synthesized carbocyclic nucleoside **3d** opens the door to carry out additional studies of structure–activity relationships as well as studies of the mode of action.

## Conclusions

In summary, a highly stereoselective route to enantiomerically pure 4′-hydroxymethyl-3′-hydroxybicyclo[4.1.0]heptyl NAs has been finely tuned, from a common azide derivative **6**, which in turn derives from 1,4-cyclohexandione. Key achievements in the asymmetric synthesis of these CNAs have been the highly diastereoselective allylic oxidation and hydroboration reactions. Four of them, **1a**, its epimer **2**, the related alkene derivative **19**, and **1b**, contain thymine and guanine, respectively, which have been constructed stepwise from **6**. The other five CNAs, **3a**–**3e**, incorporate 1,2,3-triazoles as nucleobase and have been prepared from **6** in excellent yields via cycloadditions. It is noteworthy that, although most of the compounds do not display relevant antiviral activity, compound **3d** has shown interesting antiviral activity against coxsackie B4 virus. Because to date there are no approved treatments against this virus, this preliminary result prompts us to further study this privileged scaffold.

## Experimental Section

### General Methods

Unless otherwise indicated, all reagents and solvents were purchased from commercial sources and used directly as received. Solvents were dried by distillation over the appropriate drying agents. In the reaction mixtures that require heating, the indicated temperatures refer to those of the used oil bath. All the reactions were monitored by analytical thin-layer chromatography (TLC) using silica gel 60 F254 pre-coated aluminum plates (0.25 mm thickness). TLC spots were detected under UV light and/or by charring with a KMnO_4_/KOH aqueous solution or Vanillin solution. Flash column chromatography was performed using silica gel (230–400 mesh). ^1^H NMR spectra were recorded using 400 MHz and were referenced to the residual proton signals of CDCl_3_, 7.26 ppm, and MeOH-*d*_4_, 3.31 ppm. ^13^C[^1^H] NMR spectra were recorded at 100 MHz and were referenced to the residual ^13^C signal of CDCl_3_, 77.16 ppm, and MeOH-*d*_4_, 49.00 ppm. Structural assignments were made with additional information from gCOSY, gHSQC, and gHMBC experiments. Melting points were determined on a hot stage and are uncorrected. Optical rotations were measured at 20 ± 2 °C at the sodium D line (589 nm) in a microcell (0.1 dm). Infrared spectra were recorded on a spectrophotometer equipped with a Golden Gate Single Refraction Diamond ATR (Attenuated Total Reflectance) accessory. High-resolution mass spectra were recorded using electrospray ionization (ESI).

MW reactions were conducted on a CEM Discover Microwave synthesizer. The machine includes a continuous focused MW-power delivery system with operator-selectable power output from 0 to 300 W. The temperature inside the vessel was monitored by a calibrated infrared temperature control attached under the reaction vessel. All experiments were carried out under stirring by means of a rotating magnetic plate located below the floor of the MW cavity and a Teflon-coated magnetic stir bar in the vessel. All the experiments were carried out in a sealed reaction vessel.

#### Evaluation of Antiviral Activity

##### Antiviral Activity Assays

The antiviral screening of compounds **1a**, **1b**, **2**, **19**, and **3a**–**e** was performed against herpes simplex virus-1 (KOS) [HSV-1 KOS], herpes simplex virus-2 (G) [HSV-2 G], Vaccinia virus [VV], adenovirus 2, and human coronavirus (229E) in HEL cell cultures; vesicular stomatitis virus [VSV], coxsackie virus B4, and respiratory syncytial virus in Hep-2 cell cultures; Reovirus-1, Sindbis virus, coxsackie virus B4, Punta Toro virus, yellow fever virus, and Zika virus in Vero cell cultures; Influenza A virus A/Ned/378/05A (H1N1), Influenza A virus A/HK/7/87 (H3N2), and Influenza B virus B/Ned/537/05 in MDCK cell cultures; and human immunodeficiency virus type 1 (HIV-1) (IIIB) and HIV-2 (ROD) in MT-4 cell cultures. The results were expressed as the 50% effective concentration (EC50) or drug concentration required to inhibit virus-induced cytopathicity by 50%. Read-out was through microscopical inspection or the MTS viability staining method.

Confluent cell cultures in microtiter 96-well plates were inoculated with 100 CCID50 of the virus (one CCID50 being the dose of the virus sufficient to infect 50% of the cell cultures) in the presence of varying concentrations of the test compounds. Viral cytopathicity was recorded as soon as it reached completion in the control virus-infected cell cultures that had not been treated with the test compounds.

##### Cytotoxic Assays

The cytotoxicity of the compounds was evaluated in parallel with their antiviral activity in uninfected cell cultures and is expressed as the 50% cytotoxic concentration (CC50) as determined by measuring the cell viability of normal cell morphology (HEL, Hep-2, MDCK, and Vero cells) with the colorimetric formazan-based MTS assay.

### (1*R*,4′*R*,5*R*,5′*R*,6*S*)-5-(Benzyloxy)-4′,5′-diphenylspiro[bicyclo[4.1.0]-heptane-2,2′-[1,3]dioxolane] (**7**)

To an ice-cooled solution of alcohol **4**(26) (3.02 g, 9.37 mmol) in anhydrous THF (90 mL), NaH (1.27 g, 31.8 mmol) was added in one portion and the mixture was stirred for 1 h at 0 °C. Then, benzyl bromide (1.3 mL, 11.24 mmol) and sodium iodide (1.73 g, 11.5 mmol) were added at 0 °C and the mixture was stirred at the reflux temperature for 3 h. After that time, water (60 mL) was carefully added at RT and stirred for 15 min. Then, EtOAc (30 mL) was added, and the aqueous layer was extracted with more EtOAc (2 × 30 mL). The organic layers were dried (Na_2_SO_4_), concentrated under reduced pressure, and purified by column chromatography (SiO_2_, hexanes:EtOAc, 6:1) to furnish **7** (3.29 g, 7.96 mmol, 85% yield) as a pale oil.

### 4

^1^H NMR (400 MHz, CDCl_3_) δ 7.37–7.28 (m, 8H, H-Ar), 7.23–7.19 (m, 2H, H-Ar), 4.83 (d, *J*_5′,4′_ = 9.0 Hz, 1H, H-5′), 4.70 (d, *J*_4′,5′_ = 9.0 Hz, 1H, H-4′), 4.35 (q, *J*_5,4ax_ = *J*_5,4eq_ = *J*_5,6_ = 5.5 Hz, 1H, H-5), 1.94–1.81 (m, 3H, 2H-3, H-4 eq), 1.70–1.55 (m, 2H, H-1, H-6), 1.56–1.41 (m, 1H, H-4ax), 0.96 (q, *J*_7endo,1_ = *J*_7endo,6_ = *J*_gem_ = 5.7 Hz, 1H, H-7endo), 0.83 (td, *J*_7exo,1_ = *J*_7exo,6_ = 9.4 Hz, *J*_gem_ = 5.7 Hz, 1H, H-7exo).

### 7

*R*_f_ = 0.68 (hexanes:EtOAc, 1:1); [α]_D_^20^ + 45.6 (*c* 1.03, CHCl_3_); ^1^H NMR (400 MHz, CDCl_3_) δ 7.46 (m, 3H, H-Ar), 7.43–7.34 (m, 8H, H-Ar), 7.32–7.28 (m, 4H, H-Ar), 4.94 (d, *J*_5′,4′_ = 8.4 Hz, 1H, H-5′), 4.86–4.75 (m, 2H, H-4′, C*H*_2_-Ph), 4.59 (d, *J*_gem_ = 11.8 Hz, 1H, C*H*_2_-Ph), 4.15 (q, *J*_5,6_ = *J*_5,4_ = 5.0 Hz, 1H, H-5), 1.99–1.80 (m, 4H, H-4, H-3), 1.72–1.58 (m, 2H, H-6, H-1), 1.12 (q, *J*_gem_ = *J*_7endo,1_ = *J*_7endo,6_ = 5.7 Hz, 1H, H-7endo), 1.03 (td, *J*_7exo,6_ = *J*_7exo,1_ = 9.1 Hz, *J*_gem_ = 5.7 Hz, 1H, H-7exo); ^13^C{^1^H} NMR (100 MHz, CDCl_3_) δ 139.1 (C*Ar*), 137.1/136.9 (C*Ar*), 128.5/128.5/128.4/128.3/127.8/127.5/126.9/126.7 (C*Ar*), 109.8 (C2), 85.6/85.3 (C4′,C5′), 70.7 (C5), 69.6 (*C*H_2_Ph), 30.2 (C3), 26.6 (C4), 22.4 (C1), 16.5 (C6), 5.5 (C7); IR (ATR) 3063, 2850, 2524, 2108, 1729, 1495, 1453, 1060, 697 cm^–1^; HRMS (ESI+) *m/z*: [M + H]^+^ calcd for C_28_H_29_O_3_ 413.2117; found 413.2119.

### (1*R*,5*R*,6*S*)-5-(Benzyloxy)bicyclo[4.1.0]heptan-2-one (**8**)

To a stirred solution of ketal **7** (5.03 g, 12.19 mmol) in CH_2_Cl_2_ (120 mL), a 4:1 mixture of TFA (2.8 mL, 36.57 mmol)/H_2_O (700 μL) was added at RT. The reaction mixture was allowed to stir for 2 h. Then, additional CH_2_Cl_2_ (30 mL) was added, and the resulting solution was washed with saturated aqueous NaHCO_3_ solution (100 mL). The organic layers were dried (Na_2_SO_4_), concentrated under reduced pressure, and purified by column chromatography (CHCl_3_ 100%) to provide **8** (2.401 g, 11.10 mmol, 91% yield) as a yellowish oil. *R_f_* = 0.22 (CHCl_3_); [α]_D_^20^ + 62.6 (*c* 1.45, CHCl_3_); ^1^H NMR (400 MHz, CDCl_3_) δ 7.39–7.34 (m, 4H, H-Ar), 7.32–7.28 (m, 1H, H-Ar), 4.73 (d, *J*_gem_ = 11.9 Hz, 1H, C*H*_2_-Ph), 4.64 (d, *J*_gem_ = 11.9 Hz, 1H, C*H*_2_-Ph), 4.15 (dt, *J*_5,4ax_ = 9.3 Hz, *J*_5,4eq_ = *J*_5,6_ = 4.9 Hz, 1H, H-5), 2.42 (dt, *J*_gem_ = 17.7 Hz, *J*_3eq,4_ = 5.5 Hz, 1H, H-3 eq), 2.12 (ddd, *J*_gem_ = 17.7 Hz, *J*_3ax,4x_ = 10.7 Hz, *J*_3ax,4eq_ = 6.5 Hz, 1H, H-3ax), 2.05–1.93 (m, 2H, H-4), 1.88–1.71 (m, 2H, H-1, H-6), 1.50 (q, *J*_gem_ = *J*_7endo,6_ = *J*_7endo,1_ = 5.4 Hz, 1H, H-7endo), 1.24 (td, *J*_7exo,6_ = *J*_7exo,1_ = 9.1 Hz, *J*_gem_ = 5.4 Hz, 1H, H-7exo); ^13^C{^1^H} NMR (100 MHz, CDCl_3_) δ 208.2 (C2), 138.5 (C*Ar*), 128.6/128.2/127.9/127.8 (C*Ar*), 71.3 (C5), 70.6 (*C*H_2_Ph), 34.4 (C3), 26.5 (C4), 25.5 (C1), 21.3 (C6), 9.5 (C7); IR (ATR) 3028, 2857, 1692, 1342, 1075, 1028, 881, 631 cm^–1^; HRMS (ESI+) *m/z*: [M + H]^+^ calcd for C_14_H_17_O_2_ 217.1229; found 217.1231.

### (1*S*,2*R*,6*R*)-2-(Benzyloxy)-5-methylidenebicyclo[4.1.0]heptane (**5**) and (1*R*,3*R*,5*R*,6*S*)-5-(benzyloxy)-2-methylidenebicyclo[4.1.0]heptan-3-ol (**9**)

To a stirring solution of Ph_3_PCH_3_I (11.82 g, 29.2 mmol) in anhydrous THF (30 mL) at 0 °C, *t-*BuOK (3.31 g, 29.5 mmol) was added, under a nitrogen atmosphere, and the resulting yellow mixture was allowed to react for 1 h. At this time, a solution of ketone **8** (1.27 g, 5.8 mmol) in anhydrous THF (10 mL) was added and the mixture was stirred for 3 h. Then, diethyl ether (30 mL) was added, and the crude filtered through a pad of silica and Celite, using more diethyl ether (100 mL) as eluent. The volatiles were removed under vacuum to obtain **5** as an orange oil, which is unstable was directly used for the next step without further purification. Accordingly, crude alkene **5** was rapidly dissolved in CH_2_Cl_2_ (75 mL) and SeO_2_ (128 mg, 1.16 mmol) and *t-*BuOOH (440 μL, 6.38 mmol, 70% in water) were sequentially added at RT. After stirring for 20 min, water (50 mL) was added, and the aqueous phase was extracted with more CH_2_Cl_2_ (2 × 30 mL). Finally, the organic layers were dried with anhydrous Na_2_SO_4_, concentrated under reduced pressure, and purified by column chromatography (hexanes:EtOAc, 5:1) to provide allylic alcohol **9** (1.05 g, 4.55 mmol, 79% overall yield from **8**) as brown oil.

An aliquot of compound **5** was purified by column chromatography (hexanes:EtOAc, 5:1); however, due to its instability, the obtained HRMS analysis was not within the 10 ppm acceptable error: *R_f_* = 0.75 (hexanes:EtOAc, 2:1); [α]_D_^20^ = +47.6 (*c* 0.98, CHCl_3_); ^1^H NMR (400 MHz, CDCl_3_) δ 7.46–7.25 (m, 5H, H-Ar), 4.90 (br s, 1H, H-1′), 4.79 (br s, 1H, H-1′), 4.74 (d, *J*_gem_ = 11.9 Hz, 1H, C*H*_2_-Ph), 4.56 (d, *J*_gem_ = 11.9 Hz, 1H, C*H*_2_-Ph), 4.05 (q, *J*_2,3ax_ = *J*_2,3eq_ = *J*_2,1_ = 5.9 Hz, 1H, H-2), 2.21 (dddt, *J*_gem_ = 14.9 Hz, *J*_4ax,3ax_ = 8.5 Hz, *J*_4ax,3eq_ = 4.4 Hz, *J*_4ax,1′_ = 1.4 Hz, 1H, H-4ax), 2.03 (dddt, *J*_gem_ = 14.9 Hz, *J*_4eq,3ax_ = 7.2 Hz, *J*_4eq,3eq_ = 4.3 Hz, *J*_4eq,1′_ = 1.4 Hz, 1H, H-4 eq), 1.88–1.75 (m, 1H, H-6), 1.71–1.50 (m, 3H, H-1, H-3), 0.94–0.84 (m, 2H, H-7); ^13^C{^1^H} NMR (100 MHz, CDCl_3_) δ 146.3 (C5), 139.1 (C*ipso*), 128.4/127.7/127.5 (C*Ar*), 108.1 (C1′), 72.0 (C2), 69.7 (*C*H_2_Ph), 28.8 (C4), 27.7 (C3), 19.9 (C1), 17.0 (C6), 8.7 (C7); IR (ATR) 3070, 2930, 1471, 1427, 1106, 806, 741 cm^–1^; HRMS (ESI+) *m/z*: [M + H]^+^ calcd for C_15_H_19_O 215.1436; found 215.1465.

### 9

*R_f_* = 0.35 (hexanes:EtOAc, 2:1); [α]_D_^20^ + 43 (*c* 1.1, CHCl_3_); ^1^H NMR (400 MHz, CDCl_3_) δ 7.39–7.32 (m, 4H, H-Ar), 7.30–7.27 (m, 1H, H-Ar), 5.06 (d, *J*_gem_ = 10.0 Hz, 2H, H-1′), 4.71 (d, *J*_gem_ = 11.8 Hz, 1H, C*H*_2_-Ph), 4.52 (d, *J*_gem_ = 11.8 Hz, 1H, C*H*_2_-Ph), 4.29 (q, *J*_5,6_ = *J*_5,4eq_ = *J*_5,4ax_ = 6.0 Hz, 1H, H-5), 4.22 (dd, *J*_3,4ax_ = 8.3 Hz, *J*_3,4eq_ = 3.2 Hz, 1H, H-3), 1.87 (ddd, *J*_1,7exo_ = 9.3 Hz, *J*_1,6_ = 7.8 Hz, *J*_1,7endo_ = 5.0 Hz, 1H, H-1), 1.77 (ddd, *J*_gem_ = 13.5 Hz, *J*_4ax,3_ = 8.3 Hz, *J*_4ax,5_ = 6.0 Hz, 1H, H-4ax), 1.70 (ddd, *J*_gem_ = 13.5 Hz, *J*_4eq,5_ = 6.0 Hz, *J*_4eq,3_ = 3.2 Hz, 1H, H-4 eq), 1.57 (m, 1H, H-6), 0.90 (td, *J*_7exo,6_ = *J*_7exo,1_ = 9.3 Hz, *J*_gem_ = 5.0 Hz, 1H, H-7exo), 0.76 (q, *J*_gem_ = *J*_7endo,6_ = *J*_7endo,1_ = 5.0 Hz, 1H, H-7endo); ^13^C{^1^H} NMR (100 MHz, CDCl_3_) δ 148.6 (C2), 138.8 (C*ipso*), 128.4 (C*Ar*), 127.7 (C*Ar*), 127.5 (CAr), 109.2 (C1′), 70.8 (C5), 69.8 (*C*H_2_Ph), 67.6 (C3), 37.0 (C4), 19.9 (C1), 16.4 (C6), 9.0 (C7); IR (ATR) 3399, 3065, 2857, 1640, 1454, 1200, 1066, 824, 698 cm^–1^; HRMS (ESI+) *m/z*: [M + H]^+^ calcd for C_15_H_19_O_2_ 231.1385; found 231.1375.

### (1*R*,2*R*,3*R*,5*R*,6*S*)-5-(Benzyloxy)-2-(hydroxymethyl)bicyclo[4.1.0]heptan-3-ol (10) and Its (2*S*)-Diastereoisomer (**11**)

To a stirred solution of allylic alcohol **9** (1.750 g, 7.60 mmol) in anhydrous THF (80 mL), 9-borabicyclo[3.3.1]nonane solution (9-BBN, 38 mL, 37.99 mmol, 0.5 M in THF) was added at −10 °C. The mixture was allowed to slowly warm to RT and stirred overnight. Then, water (18 mL), NaOH (35 mL, 3 M in water), and H_2_O_2_ (35 mL, 30% in water) were added at 0 °C. After stirring for 15 min at RT, the mixture was diluted with brine (150 mL) and CH_2_Cl_2_ (150 mL) and the aqueous phase was extracted with CH_2_Cl_2_ (2 × 100 mL). The organic layers were dried (Na_2_SO_4_), concentrated under reduced pressure, and purified by column chromatography (CH_2_Cl_2_ 100% to CH_2_Cl_2_:MeOH 20:1 to 10:1) to provide a mixture of alcohols **10** and **11** (1.724 g, 6.94 mmol, 92% overall yield, 2 steps) in a ca. 20:1 diastereomeric ratio as a white solid. After repeated purification by column chromatography, it was possible to obtain a pure fraction of **10**: *R_f_* = 0.1 (hexanes:EtOAc, 1:1); Mp 73–75 °C (from Et_2_O); [α]_D_^20^ + 104 (*c* 1.0, CHCl_3_); ^1^H NMR (400 MHz, CDCl_3_) δ 7.39–7.30 (m, 4H, H-Ar), 7.29–7.24 (m, 1H, H-Ar), 4.73 (d, *J*_gem_ = 11.8 Hz, 1H, C*H*_2_-Ph), 4.55 (d, *J*_gem_ = 11.8 Hz, 1H, C*H*_2_-Ph), 4.30 (dt, *J*_5,4ax_ = 9.5 Hz, *J*_5,4eq_ = *J*_5,6_ = 6.1 Hz, 1H, H-5), 4.08 (br m, 1H, H-3), 3.91–3.80 (m, 2H, H-1′), 2.60 (t, *J* = 5.2 Hz, 1H, OH), 2.47 (d, *J* = 3.8 Hz, 1H, OH), 2.06 (dt, *J*_gem_ = 13.8 Hz, *J*_4eq,5_ = *J*_4eq,3_ = 6.1 Hz, 1H, H-4 eq), 1.76 (tt, *J*_2,1′_ = 6.2 Hz, *J*_2,1_ = J_2,3_ = 2.8 Hz, 1H, H-2), 1.39 (tt, *J*_6,7exo_ = *J*_6,1_ = 8.8 Hz, *J*_6,7endo_ = *J*_6,5_ = 5.9 Hz, 1H, H-6), 1.18 (ddd, *J*_gem_ = 13.8 Hz, *J*_4ax,5_ = 9.5 Hz, *J*_4x,3_ = 1.8 Hz, 1H, H-4ax), 0.93 (tdd, *J*_1,7exo_ = *J*_1,6_ = 8.8 Hz, *J*_1,7endo_ = 5.5 Hz, *J*_1,2_ = 2.8 Hz, 1H, H-1), 0.81 (td, *J*_7exo,6_ = *J*_7exo,1_ = 8.8 Hz, *J*_gem_ = 4.9 Hz, 1H, H-7exo), 0.39 (q, *J*_gem_ = *J*_7endo,6_ = *J*_7endo,1_ = 5.5 Hz, 1H, H-7endo); ^13^C{^1^H} NMR (100 MHz, CDCl_3_) δ 139.0 (C*ipso*), 128.5/127.9/127.6 (C*Ar*), 70.7 (C5), 69.9 (CH_2_Ph), 69.0 (C3), 65.2 (C1′), 41.2 (C2), 33.0 (C4), 13.8 (C6), 13.0 (C1), 8.6 (C7); IR (ATR) 3350, 2872, 2365, 1497, 1454, 1068, 739 cm^–1^; HRMS (ESI+) *m/z*: [M + H]^+^ calcd for C_15_H_21_O_3_ 249.1491; found 249.1466.

### (1*S*,2*R*,4*R*,5*R*,6*R*)-5-(Hydroxymethyl)bicyclo[4.1.0]heptane-2,4-diol (**12**)

A stirred solution of a 20:1 mixture of diols **10** and **11** (101 mg, 0.40 mmol) in EtOH (4 mL) at RT was hydrogenated in the presence of 10% Pd/C (10 mg, 10 wt %) at 2 atm for 24 h. Then, the mixture was filtered through a short pad of Celite and rinsed with more EtOH. The solvent was evaporated under reduced pressure to give alcohol **12** (63 mg, 0.39 mmol, 95% yield) as a white solid, which was re-crystallized by vapor diffusion using MeOH/Et_2_O at RT Mp 166–167 °C (from MeOH–Et_2_O); [α]_D_^20^–52 (*c* 1.3, CHCl_3_); ^1^H NMR (400 MHz, CDCl_3_) δ 4.47 (dt, *J*_2,3ax_ = 10.8 Hz, *J*_2,1_ = *J*_2,3eq_ = 5.8 Hz, 1H, H-2), 3.93 (br t, *J*_4,3eq_ = *J*_4,3eq_ = 4.3 Hz, 1H, H-4), 3.72 (dd, *J*_gem_ = 10.6 Hz, *J*_1′a,5_ = 7.0 Hz, 1H, H-1′a), 3.65 (dd, *J*_gem_ = 10.6 Hz, *J*_1′b,5_ = 7.0 Hz, 1H, H-1′b), 1.95 (dt, *J*_gem_ = 13.3 Hz, *J*_3eq,2_ = *J*_3eq,4_ = 5.8 Hz, 1H, H-3 eq), 1.58 (tt, *J*_5,1′a_ = *J*_5,1′b_ = 7.0 Hz, *J*_5,6_ = *J*_5,4_ = 3.0 Hz, 1H, H-5), 1.28 (tt, *J*_1,7exo_ = *J*_1,6_ = 8.8 Hz, *J*_1,7endo_ = *J*_1,4_ = 5.8 Hz, 1H, H-1), 1.00 (ddd, *J*_gem_ = 13.3 Hz, *J*_3ax,2_ = 10.8 Hz, *J*_3ax,4_ = 1.6 Hz, 1H, H-3ax), 0.83 (tdd, *J*_6,7exo_ = *J*_6,1_ = 8.8 Hz, *J*_6,7endo_ = 5.3 Hz, *J*_6,5_ = 2.4 Hz, 1H, H-6), 0.73 (td, *J*_7exo,1_ = *J*_7exo,6_ = 8.8 Hz, *J*_gem_ = 4.9 Hz, 1H, H-7exo), 0.27 (q, *J*_gem_ = *J*_7endo,6_ = *J*_7endo,1_ = 5.3 Hz, 1H, H-7endo); ^13^C{^1^H} NMR (100 MHz, CDCl_3_) δ 68.4 (C4), 65.0 (C1′), 64.7 (C2), 43.9 (C5), 36.0 (C3), 17.9 (C1), 14.0 (C6), 9.6 (C7); IR (ATR) 3324, 3239, 3019, 2925, 1405, 1250, 1040, 906 cm^–1^; HRMS (ESI-) *m/z*: [M + HCOO]^−^ calcd for C_9_H_15_O_5_ 203.0919; found 203.0919.

### [(1′*R*,2′*R*,3′*R*,5′*R*,6′*S*)-3′-(Benzoyloxy)-5′-(benzyloxy)bicyclo[4.1.0]hept-2′-yl]methyl benzoate (**13**) and Its (2′*S*)-diastereoisomer (**14**)

To a 20:1 mixture of diols **10** and **11** (1.079 g, 4.35 mmol) in dry pyridine (70 mL), benzoyl chloride (1.76 mL, 15.15 mmol) was added dropwise at RT. The solution was heated to reflux in an oil bath for 2 h. Then, the solution was cooled to 70 °C and quenched with MeOH (6 mL). After 30 min, the solvent was removed under reduced pressure and the residue was partitioned between EtOAc (100 mL) and water (100 mL). The organic layer was washed with 1 M HCl (2 × 70 mL) and aqueous saturated NaHCO_3_ solution (2 × 70 mL) and dried with Na_2_SO_4_. The filtrate was concentrated under reduced pressure, and the residue was purified by column chromatography (hexanes:EtOAc, 10:1 → 8:1 → 5:1) to afford alcohols **13** (1.826 g, 4.00 mmol, 92% yield) as a clear oil and **14** (98 mg, 0.218 mmol, 5% yield) also as a clear oil.

### 13

*R_f_* = 0.25 (hexanes:EtOAc, 5:1); [α]_D_^20^ + 16.9 (*c* 2.13, CHCl_3_); ^1^H NMR (400 MHz, CDCl_3_) δ 8.06–7.98 (m, 4H, H-Ar), 7.63–7.53 (m, 2H, H-Ar), 7.45 (m, 4H, H-Ar), 7.40–7.24 (m, 5H, H-Ar), 5.50–5.44 (m, 1H, H-3′), 4.74 (d, *J*_gem_ = 11.5 Hz, 1H, C*H*_2_-Ph), 4.63–4.52 (m, 2H, C*H*_2_-Ph, H-1), 4.37 (dd, *J*_gem_ = 10.8 Hz, *J*_1,2′_ = 8.3 Hz, 1H, H-1), 4.29 (dt, *J*_5′,4′ax_ = 10.0 Hz, *J*_5′,4′eq_ = *J*_5′,6′_ = 6.0 Hz, 1H, H-5′), 2.46 (dt, *J*_gem_ = 14.1 Hz, *J*_4′eq,5′_ = *J*_4′eq,3′_ = 6.0 Hz, 1H, H-4′eq), 2.34 (ddd, *J*_2′,1_ = 8.3 Hz, *J*_2′,1_ = 6.3 Hz, *J*_2′,3′_ = 3.3 Hz, 1H, H-2′), 1.56 (tt, *J*_6′,1′_ = *J*_6′,7′exo_ = 8.7 Hz, *J*_6′,7′endo_ = *J*_6′,5′_ = 5.8 Hz, 1H, H-6′), 1.35 (ddd, *J*_gem_ = 14.1 Hz, *J*_4′ax,5′_ = 10.1 Hz, *J*_4′ax,3′_ = 1.7 Hz, 1H, H-4′ax), 1.11 (tdd, *J*_1′,7′exo_ = *J*_1′,6′_ = 8.7 Hz, *J*_1′,7′endo_ = 5.5 Hz, *J*_1′,2′_ = 2.4 Hz, 1H, H-1′), 0.97 (td, *J*_7′exo,1′_ = *J*_7′exo,6′_ = 8.7 Hz, *J*_gem_ = 5.1 Hz, 1H, H-7′exo), 0.59 (q, *J*_gem_ = *J*_7′endo,1′_ = *J*_7′endo,6′_ = 5.5 Hz, 1H, H-7′endo); ^13^C{^1^H} NMR (100 MHz, CDCl_3_) δ 166.6 (*C*O), 165.8 (*C*O), 138.8/133.1/130.3/130.1/129.74/129.67/128.6/128.50/128.48/127.9/127.6 (C*Ar*), 70.7 (C5′), 70.22 (C3′), 70.17 (*C*H_2_Ph), 65.2 (C1), 38.7 (C2′), 30.0 (C4′), 14.3 (C6′), 13.5 (C1′), 9.1 (C7′); IR (ATR) 3066, 3017, 1714, 1602, 1585, 1451, 1268, 1215, 747 cm^–1^; HRMS (ESI+) *m/z*: [M + H]^+^ calcd for C_29_H_29_O_5_ 457.2015; found 457.2015.

### 14

*R_f_* = 0.31 (hexanes:EtOAc 5:1); [α]_D_^20^ + 5.42 (*c* 1.55, CHCl_3_); ^1^H NMR (400 MHz, CDCl_3_) δ 8.10–8.00 (m, 4H, H-Ar), 7.61–7.52 (m, 2H, H-Ar), 7.47–7.36 (m, 8H, H-Ar), 7.35–7.29 (m, 1H, H-Ar), 5.24 (ddd, *J*_3′,2′_ = 10.2 Hz, *J*_3′,4′eq_ = 7.7 Hz, *J*_3′,4′ax_ = 3.1 Hz, 1H, H-3′), 4.76 (d, *J*_gem_ = 11.9 Hz, 1H, C*H*_2_-Ph), 4.58 (d, *J*_gem_ = 11.9 Hz, 1H, C*H*_2_-Ph), 4.50 (dd, *J*_gem_ = 11.1 Hz, *J*_1,2′_ = 6.3 Hz, 1H, H-1), 4.45 (dd, *J*_gem_ = 11.1 Hz, *J*_1,2′_ = 6.5 Hz, 1H, H-1), 4.33 (dt, *J*_5′,4′ax_ = 7.3 Hz, *J*_5′,4′eq_ = *J*_5′,6′_ = 5.3 Hz, 1H, H-5′), 2.73–2.66 (m, 1H, H-2′), 1.98 (ddd, *J*_gem_ = 13.6 Hz, *J*_4′ax,5′_ = 7.3 Hz, *J*_4′ax,3′_ = 3.1 Hz, 1H, H-4′ax), 1.88 (ddd, *J*_gem_ = 13.6 Hz, J_4′eq,3′_ = 7.7 Hz, *J*_4′eq,5′_ = 5.3 Hz, 1H, H-4′eq), 1.53–1.46 (m, 2H, H-1′, H-6′), 0.79 (q, *J*_gem_ = *J*_7′endo,1′_ = *J*_7′endo,6′_ = 5.6 Hz, 1H, H-7′endo), 0.72 (td, *J*_7′exo,6′_ = *J*_7′exo,1′_ = 9.0 Hz, *J*_gem_ = 5.4 Hz, 1H, H-7′exo); ^13^C{^1^H} NMR (100 MHz, CDCl_3_) δ 166.6 (*C*O), 165.8 (*C*O), 138.7/133.0/132.9/130.4/130.2/129.7/128.4/128.4/127.7/127.5 (C*Ar*), 70.7 (C5′), 69.7 (C3′), 69.6 (*C*H_2_Ph), 66.1 (C1), 37.7 (C2′), 33.4 (C4′), 15.5 (C6′), 13.4 (C1′), 3.5 (C7′); IR (ATR) 3065, 3017, 1716, 1605, 1585, 1453, 1272, 1211, 747 cm^–1^. HRMS (ESI+) *m/z*: [M + Na]^+^ calcd for C_29_H_28_O_5_Na 479.1834; found 479.1832.

### [(1′*R*,2′*R*,3′*R*,5′*R*,6′*S*)-3′-(Benzoyloxy)-5′-hydroxybicyclo[4.1.0]hept-2′-yl]methyl Benzoate (15)

A stirred solution of **13** (1.872 g, 4.10 mmol) in EtOH (41 mL) at RT was hydrogenated in the presence of 10% Pd/C (190 mg, 10 wt %) at 2 atm for 24 h. Then, the mixture was filtered through a short pad of Celite and rinsed with EtOH. The solvent was evaporated under reduced pressure to give alcohol **15** (1.501 g, 4.08 mmol, 100% yield) as a colorless syrup. *R_f_* = 0.28 (hexanes:EtOAc, 1:1); [α]_D_^20^–25.0 (*c* 1.45, CHCl_3_); ^1^H NMR (400 MHz, CDCl_3_) δ 8.03–7.98 (m, 4H, H-*orto*), 7.60–7.49 (m, 2H, H-*para*), 7.47–7.37 (m, 4H, H-*meta*), 5.43 (br t, *J*_3′,4′ax_ = *J*_3′,4′eq_ = *J*_3′,2′_ = 4.3 Hz, 1H, H-3′), 4.57 (dd, *J*_gem_ = 10.8 Hz, *J*_1,2′_ = 6.7 Hz, 1H, H-1), 4.50 (dt, *J*_5′,4′ax_ = 11.2 Hz, *J*_5′,4′eq_ = *J*_5′,6′_ = 5.8 Hz, 1H, H-5′), 4.33 (dd, *J*_gem_ = 10.8 Hz, *J*_1,2′_ = 8.8 Hz, 1H, H-1), 2.39 (dt, *J*_gem_ = 12.4 Hz, *J*_4′eq,5′_ = *J*_4′eq,3′_ = 5.8 Hz, 1H, H-4′eq), 2.26 (ddt, *J*_2′,1_ = 8.9 Hz, *J*_2′,1_ = 6.7 Hz, *J*_2′,3′_ = *J*_2′1′_ = 4.5 Hz, 1H, H-2′), 1.63 (s, 1H, OH), 1.58–1.52 (m, 1H, H-6′), 1.28–1.14 (m, 1H, H-4′ax), 1.08 (tt, *J*_1′,7′exo_ = *J*_1′,6′_ = 8.5 Hz, *J*_1′,7′endo_ = *J*_1′,2′_ = 4.5 Hz, 1H, H-1′), 0.89 (td, *J*_7′exo,1′_ = *J*_7′exo,6′_ = 8.5 Hz, *J*_gem_ = 5.4 Hz, 1H, H-7′exo), 0.45 (q, *J*_gem_ = *J*_7′endo,6′_ = *J*_7′endo,1′_ = 5.4 Hz, 1H, H-7′endo); ^13^C{^1^H} NMR (100 MHz, CDCl_3_) δ 166.5 (*C*O), 165.8 (*C*O), 133.2 (C*para*), 133.1 (C*para*), 130.2 (C*ipso*), 130.0 (C*ipso*), 129.7 (C*orto*), 129.6 (C*orto*), 128.6 (C*meta*), 128.5 (C*meta*), 70.4 (C3′), 65.2 (C1), 63.9 (C5′), 38.5 (C2′), 32.4 (C4′), 17.4 (C6′), 13.5 (C1′), 8.6 (C7′); IR (ATR) 3489, 3005, 2953, 2338, 1713, 1601, 1450, 1265, 1108, 708 cm^–1^; HRMS (ESI+) *m/z*: [M + Na]^+^ calcd for C_22_H_22_O_5_Na 389.1365; found 389.1363.

### [(1′*R*,2′*R*,3′*R*,5′*S*,6′*S*)-5′-Azido-3′-(benzoyloxy)bicyclo[4.1.0]hept-2-yl]methyl Benzoate (**6**) and [(1′*R*,2′*R*,3′*R*,6′*R*)-3′-(Benzoyloxy)bicyclo[4.1.0]hept-4′-en-2′-yl]methyl Benzoate (**16**)

To a stirred solution of Ph_3_P (1.25 g, 4.76 mmol) in dry toluene (35 mL), DBAD (1.10 g, 4.76 mmol) was slowly added under an argon atmosphere and the mixture was stirred for 45 min at 0 °C (after 15 min, a white suspension appeared). Then, DPPA (720 μL, 3.33 mmol) and a solution of **15** (1.161 g, 3.17 mmol) in dry toluene (15 mL) were sequentially added at −10 °C. The mixture was allowed to slowly warm to RT and stirred overnight. Then, the solvent was removed, and the crude was purified by column chromatography (hexanes:EtOAc, 30:1 → 20:1 → 15:1) to provide azide **6** (794 mg, 2.03 mmol, 64% yield) as a white solid, which was re-crystallized by vapor diffusion using MeOH/Et_2_O at RT, and the elimination product **16** (98 mg, 0.28 mmol, 9% yield) as a colorless syrup.

### 6

*R_f_* = 0.31 (hexanes:EtOAc, 3:1); Mp 109–108 °C (from MeOH–Et_2_O); [α]_D_^20^–73.9 (*c* 0.98, CHCl_3_); ^1^H NMR (400 MHz, CDCl_3_) δ 8.08 (d, *J_orto,meta_* = 7.5 Hz, 2H, H-*orto*), 8.00 (d, *J_orto,meta_* = 7.5 Hz, 2H, H-*orto*), 7.58–7.52 (m, 2H, H-*para*), 7.42 (m, 4H, H-*meta*), 5.25 (ddd, J_3′,4′eq_ = 6.5 Hz, *J*_3′,2′_ = 4.1 Hz, *J*_3′,4ax_ = 2.8 Hz, 1H, H-3′), 4.62 (dd, *J*_gem_ = 10.9 Hz, *J*_1,2′_ = 7.2 Hz, 1H, H-1), 4.48 (dd, *J*_gem_ = 10.9 Hz, *J*_1,2′_ = 7.2 Hz, 1H, H-1), 4.02 (td, *J*_5′,4ax_ = *J*_5′,4eq_ = 5.4 Hz, *J*_5′,6′_ = 1.5 Hz, 1H, H-5′), 2.33 (tt, *J*_2′,1_ = 7.2 Hz, *J*_2′,3′_ = *J*_2′1′_ = 3.4 Hz, 1H, H-2′), 2.23 (dt, *J*_gem_ = 15.0, *J*_4′eq,5′_ = *J*_4′eq,3′_ = 5.4 Hz, 1H, H-4′eq), 1.71 (ddd, *J*_gem_ = 15.0 Hz, *J*_4′ax,5′_ = 5.4 Hz, *J*_4′ax,3′_ = 2.8 Hz, 1H, H-4′ax), 1.30 (td, *J*_6′,7′exo_ = *J*_6′,1′_ = 9.0 Hz, *J*_6′,7′endo_ = 5.3 Hz, 1H, H-6′), 1.12 (td, *J*_1′,7′exo_ = *J*_1′,6′_ = 9.0 Hz, *J*_1′,7′endo_ = 5.3 Hz, 1H, H-1′), 1.04 (td, *J*_7′endo,6_ = *J*_7′endo,1_ = 9.0 Hz, *J*_gem_ = 5.1 Hz, 1H, H-7′exo), 0.23 (q, *J*_gem_ = *J*_7′endo,6′_ = *J*_7′endo,1′_ = 5.3 Hz, 1H, H-7′endo); ^13^C{^1^H} NMR (100 MHz, CDCl_3_) δ 166.6 (*C*O), 166.2 (*C*O), 133.2 (2C, C*para*), 130.3 (C*ipso*), 130.0 (C*ipso*), 129.8 (C*orto*), 129.7 (C*orto*), 128.52 (C*meta*), 128.50 (C*meta*), 66.6 (C3′), 65.2 (C1), 55.2 (C5′), 38.3 (C2′), 29.2 (C4′), 14.1 (C6′), 11.3 (C1′), 11.0 (C7′); IR (ATR) 2922, 2109, 1709, 1599, 1449, 1267, 1248 cm^–1^; HRMS (ESI+) *m/z*: [M + H]^+^ calcd for C_22_H_22_N_3_O_4_ 392.1610; found 392.1607.

### 16

*R_f_* = 0.44 (hexanes:EtOAc, 3:1); [α]_D_^20^–35.6 (*c* 1.01, CHCl_3_); ^1^H NMR (400 MHz, CDCl_3_) δ 8.06–8.01 (m, 2H, H-*orto*), 7.94–7.87 (m, 2H, H-*orto*), 7.53–7.44 (m, 2H, H-*para*), 7.40–7.29 (m, 4H, H-*meta*), 6.27 (ddd, *J*_5′,4′_ = 10.1 Hz, *J*_5′,6′_ = 4.7 Hz, *J*_5′,3′_ = 2.4 Hz, 1H, H-5′), 5.51 (dt, *J*_3′,2′_ = 6.8 Hz, *J*_3′,4′_ = *J*_3′,5′_ = 2.4 Hz, 1H, H-3′), 5.47 (dd, *J*_4′,5′_ = 10.2 Hz, *J*_4′,3′_ = 2.0 Hz, 1H, H-4′), 4.57 (dd, *J*_gem_ = 11.0 Hz, *J*_1a,2′_ = 5.7 Hz, 1H, H-1a), 4.31 (dd, *J*_gem_ = 11.0 Hz, *J*_1b,2′_ = 7.0 Hz, 1H, H-1b), 3.10 (qd, *J*_2′,3′_ = *J*_2′1a_ = *J*_2′,1b_ = 6.7 Hz, *J*_2′,1′_ = 2.5 Hz, 1H, H-2′), 1.49 (tdd, *J*_1′,7′exo_ = *J*_1′,6′_ = 8.5 Hz, *J*_1′,7′endo_ = 5.7 Hz, *J*_1′,2′_ = 2.5 Hz, 1H, H-1′), 1.42 (tt, *J*_6′,7′exo_ = *J*_6′,1′_ = 8.5 Hz, *J*_6′,7′endo_ = *J*_6′,5′_ = 4.7 Hz, 1H, H-6′), 1.03 (td, *J*_7′exo,6′_ = *J*_7′exo,1′_ = 8.5 Hz, *J*_gem_ = 4.7 Hz, 1H, H-7′exo), 0.68 (dt, *J*_7′endo,1′_ = 5.7 Hz, *J*_gem_ = *J*_7′endo,6′_ = 4.7 Hz, 1H, H-7′endo); ^13^C{^1^H} NMR (100 MHz, CDCl_3_) δ 166.8 (*C*O), 166.0 (*C*O), 133.2 (C*para*), 132.9 (C*para*), 132.3 (C5′), 130.2 (C*ipso*), 130.2 (C*ipso*), 129.8 (C*orto*), 129.6 (C*orto*), 128.5 (C*meta*), 128.3 (C*meta*), 122.1 (C4′), 68.3 (C3′), 65.7 (C1), 33.0 (C2′), 13.7 (C1′), 13.2 (C7′), 9.7 (C6′); IR (ATR) 3063, 2922, 1712, 1601, 1265, 1109, 1025, 707 cm^–1^; HRMS (ESI+) *m/z*: [M + Na]^+^ calcd for C_22_H_20_O_4_Na 371.1254; found 371.1247.

### [(1′*R*,2′*R*,3′*R*,5′*S*,6′*S*)-5′-Amino-3′-(benzoyloxy)bicyclo[4.1.0]hept-2′-yl]methyl Benzoate Hydrochloride (**17**)

A stirred solution of azide **6** (615 mg, 1.57 mmol) in EtOAc (16 mL) was hydrogenated in the presence of Pd/C (61.5 mg, 10 wt %) at 2 atm for 24 h at RT. Then, the mixture was filtered through a short pad of Celite and rinsed with more EtOAc. The solvent was evaporated under reduced pressure, and the crude was treated with 2 M HCl·Et_2_O (1 mL, 2 mmol) at 0 °C and stirred for 15 min. The suspension was filtered to furnish a white solid identified as the ammonium salt **17** (618 mg, 1.54 mmol, 98% yield). Mp >210–215 °C (decomposes) (from Et_2_O); [α]_D_^20^-33.5 (*c* 0.82, CHCl_3_); ^1^H NMR (400 MHz, MeOH-*d*_4_) δ 8.05 (d, *J*_orto,meta_ = 7.4 Hz, 2H, H-*orto*), 7.92 (d, *J*_orto,meta_ = 7.8 Hz, 2H, H-*orto*), 7.65–7.52 (m, 2H, H-*para*), 7.50–7.36 (m, 4H, H-*meta*), 5.43 (dt, *J*_3′,4′ax_ = 6.3 Hz, *J*_3′,4′eq_ = *J*_3′,2′_ = 4.3 Hz, 1H, H-3′), 4.63–4.56 (m, 2H, H-1), 3.61 (br t, *J*_5′,4′eq_ = *J*_5′4′ax_ = 6.4 Hz, 1H, H-5′), 2.49 (tt, *J*_2′,1_ = 7.7 Hz, *J*_2′,3′_ = *J*_2′,1′_ = 4.0 Hz, 1H, H-2′), 2.19 (dt, *J*_gem_ = 14.7 Hz, *J*_4′eq,3′_ = *J*_4′eq,5_ = 4.9 Hz, 1H, H-4′eq), 1.99 (dt, *J*_gem_ = 14.7 Hz, *J*_4′ax,3′_ = *J*_4′ax,5′_ = 6.3 Hz, 1H, H-4′ax), 1.33–1.19 (m, 2H, H-1′,H-6′), 1.11 (td, *J*_7′endo,6_ = *J*_7′endo,1_ = 8.9 Hz, *J*_gem_ = 5.4 Hz, 1H, H-7′exo), 0.52 (q, *J*_gem_ = *J*_7′endo,6′_ = *J*_7′endo,1′_ = 5.4 Hz, 1H, H-7′endo); ^13^C{^1^H} NMR (100 MHz, MeOH-*d*_4_) δ 167.7 (*C*O), 167.2 (*C*O), 134.6 (C*para*), 134.3 (C*para*), 131.1 (C*ipso*), 131.0 (C*ipso*), 130.7 (C*orto*), 130.5 (C*orto*), 129.7 (C*meta*), 129.5 (C*meta*), 69.0 (C3′), 65.9 (C1), 47.6 (C5′), 38.3 (C2′), 30.6 (C4′), 13.9 (C6′), 12.3 (C1′), 11.3 (C7′); IR (ATR) 3048, 2922, 1714, 1615, 1514, 1269, 1099, 708 cm^–1^; HRMS (ESI+) *m/z*: [M]^+^ calcd for C_22_H_24_NO_4_ 366.1705; found 366.1703.

### 1-((1′*S*,2′*S*,4′*R*,5′*R*,6′*R*)-4′-Hydroxy-5′-(hydroxymethyl) bicyclo[4.1.0]heptan-2′-yl)-5-methylpyrimidine-2,4(1*H*,3*H*)-dione (**1a**)

To a suspension of silver cyanate (235 mg, 1.57 mmol), previously dried over phosphorus pentoxide at 80 °C in a laboratory oven for 3 h, in dry toluene (3 mL), a solution of (*E*)-3-ethoxy-2-methylacryloyl chloride (196 mg, 1.32 mmol) was added dropwise in dry toluene (0.8 mL). The heterogeneous mixture was refluxed in an oil bath under an argon atmosphere for 1.5 h before allowing it to cool to RT. The precipitate was allowed to settle, and the supernatant was transferred via a cannula to a dry Schlenk flask. The precipitate was further washed with a small quantity of dry CH_2_Cl_2_ (1 mL) and transferred to the same flask to deliver a solution of the isocyanate **18**. This solution was cooled to −78 °C, and a solution of the ammonium salt **17** (175 mg, 0.44 mmol) with Et_3_N (65 μL, 0.47 mmol) in dry CH_2_Cl_2_ (1.5 mL) was added dropwise over 3 min. The solution was allowed to warm slowly to RT and stirred overnight (16 h). EtOH (3 mL) was added, and the reaction mixture was concentrated in vacuo. To the crude residue, EtOH (4 mL) and 2 M HCl (1.25 mL, 2.5 mmol) were added. The reaction mixture was refluxed overnight in an oil bath (20 h) then cooled to RT, and the solution was concentrated in vacuo. The residue was dissolved in a 33% solution of methylamine in EtOH (50 mL) in a sealed flask and stirred for 48 h at RT. Then, the mixture was concentrated under reduced pressure and purified by column chromatography (CH_2_Cl_2_:MeOH, 20:1 → 15:1) to provide the NA **1a** (92 mg, 0.36 mmol, 79% overall yield) as a white solid, which was crystallized by vapor diffusion using the MeOH–Et_2_O mixture at low temperature. *R_f_* = 0.26 (CH_2_Cl_2_:MeOH 1:1); Mp 189–191 °C (from MeOH/Et_2_O); [α]_D_^20^ + 63.8 (*c* 0.47, MeOH); ^1^H NMR (400 MHz, MeOH-*d*_4_) δ 7.82 (q, *J*_6,CH3_ = 1.1 Hz, 1H, H-6), 4.77 (td, *J*_2′,3′ax_ = *J*_2′,3′eq_ = 6.3, *J*_2′,1′_ = 1.5 Hz, 1H, H-2′), 3.89 (dd, *J*_gem_ = 10.7 Hz, *J*_1″a,5′_ = 6.4 Hz, 1H, H-1″), 3.86–3.80 (m, 2H, H-4′, H-1″), 1.89 (d, *J*_CH3,6_ = 1.1 Hz, 3H, C*H*_3_), 1.87–1.82 (m, 2H, H-3′eq, H-5′), 1.63 (ddd, *J*_gem_ = 14.2 Hz, *J*_3′ax,2′_ = 6.3 Hz, *J*_3′ax,4′_ = 2.9 Hz, 1H, H-3′ax), 1.17 (tdd, *J*_6′,7′exo_ = *J*_6′,1′_ = 8.5 Hz, *J*_6′,7′endo_ = 5.5 Hz, *J*_6′5′_ = 2.6 Hz, 1H, H-6′), 1.06 (tdd, *J*_1′,7′exo_ = *J*_1′6′_ = 8.5 Hz, *J*_1′7′endo_ = 6.2 Hz, *J*_1′2′_ = 1.5 Hz, 1H, H-1′), 0.91 (td, *J*_7′exo,6′_ = *J*_7′exo,1′_ = 8.5 Hz, *J*_gem_ = 5.3 Hz, 1H, H-7′exo), 0.23 (q, *J*_gem_ = *J*_7′endo,1′_ = *J*_7′endo,6′_ = 5.3 Hz, 1H, H-7′endo); ^13^C{^1^H} NMR (100 MHz, MeOH-*d*_4_) δ 166.7 (C2), 153.0 (C4), 141.9 (C6), 110.2 (C5), 65.7 (C4′), 64.4 (C1″), 52.1 (C2′), 42.4 (C5′), 33.6 (C3′), 14.5 (C1′), 13.6 (C6′), 12.5 (*C*H_3_), 11.1 (C-7′); IR (ATR) 3483, 3270, 3019, 2885, 2360, 1700, 1660, 1269, 1002, 847 cm^–1^; HRMS (ESI+) *m/z*: [M + H]^+^: calcd for C_13_H_19_N_2_O_4_ 267.1345; found 267.1344.

### 1-[(1′*S*,2′*S*,4′*S*,5′*R*,6′*R*)-4′-Hydroxy-5′-(hydroxymethyl)bicyclo[4.1.0]hept-2′-yl]-5-methylpyrimidine-2,4(1*H*,3*H*)-dione (**2**) and 1-[(1′*S*,2′*S*,6′*R*)-5′-(hydroxymethyl)-bicyclo[4.1.0]hept-4′-en-2′-yl]-5-methylpyrimidine-2,4(1*H*,3*H*)-dione (**19**)

A solution of compound **1a** (30 mg, 0.11 mmol), triphenylphosphine (118 mg, 0.45 mmol), and benzoic acid (55 mg, 0.45 mmol) in a mixture of dry benzene/acetonitrile (5:1, 2.5 mL) was stirred at 0 °C under an argon atmosphere. Diethyl azodicarboxylate (DEAD, 71 μL, 0.45 mmol) was added dropwise during 1 min, the ice bath was removed, and the mixture was stirred at RT for 12 h. The solvent was evaporated to dryness, and the residue was purified by column chromatography (CH_2_Cl_2_:MeOH, 20:1) to provide a white solid still contaminated with Ph_3_P. The solid was dissolved in a solution of NH_3_ in MeOH (7 M, 2 mL) in a sealed flask and stirred for 48 h at RT. Then, the mixture was concentrated under reduced pressure and purified by column chromatography (slow gradient of CH_2_Cl_2_:MeOH, 40:1 → 30:1 → 20:1 → 15:1 → 10:1) to provide the substitution product **2** (11 mg, 4.1 μmol, 37% overall yield) as a white solid and the elimination product **19** (5 mg, 2.0 μmol, 18% overall yield) as a white solid**.**

### 2

*R_f_* = 0.15 (CH_2_Cl_2_:MeOH, 15:1); [α]_D_^20^ + 39.8 (*c* 0.32, MeOH); ^1^H NMR (400 MHz, MeOH-*d*_4_) δ 7.98 (q, *J*_6,CH3_ = 1.2 Hz, 1H, H-6), 5.00 (td, *J*_2′,3′ax_ = *J*_2′3′eq_ = 3.8 Hz, *J*_2′1′_ = 1.2 Hz, 1H, H-2′), 3.88 (dd, *J*_gem_ = 10.6 Hz, *J*_1″a,5′_ = 3.5 Hz, 1H, H-1″a), 3.75 (dd, *J*_gem_ = 10.6 Hz, *J*_1″b,5′_ = 3.5 Hz, 1H, H-1″b), 3.61 (ddd, *J*_4′,3′ax_ = 12.2 Hz, *J*_4′,5′_ = 9.1 Hz, *J*_4′,3eq_ = 3.8 Hz, 1H, H-4′), 1.89 (d, *J*_CH3,6_ = 1.2 Hz, 3H, C*H*_3_), 1.85 (dt, *J*_gem_ = 14.2 Hz, *J*_3′eq,2′_ = *J*_3′eq,4′_ = 3.8 Hz, 1H, H-3′eq), 1.64 (dtd, *J*_5′,4′_ = 9.1 Hz, *J*_5′,1″a_ = *J*_5′,1″b_ = 3.5 Hz, *J*_5′,6′_ = 1.3 Hz, 1H, H-5′), 1.49 (ddd, *J*_gem_ = 14.2 Hz, *J*_3′ax,4′_ = 12.2 Hz, *J*_3′ax,2′_ = 3.8 Hz, 1H, H-3′ax), 1.27 (dddd, *J*_6′,7′exo_ = 9.3 Hz, *J*_6′,1′_ = 7.7 Hz, *J*_6′,7′endo_ = 5.3 Hz, *J*_6′,5′_ = 1.3 Hz, 1H, H-6′), 1.08 (dddd, *J*_1′,7′exo_ = 9.3 Hz, *J*_1′,6′_ = 7.7 Hz, *J*_1′,7′endo_ = 5.3 Hz, *J*_1′,2′_ = 1.2 Hz, 1H, H-1′), 0.91 (td, *J*_7′exo,6′_ = *J*_7′exo,1′_ = 9.3 Hz, *J*_gem_ = 5.3 Hz, 1H, H-7′exo), 0.39 (q, *J*_gem_ = *J*_7′endo,1′_ = *J*_7′endo,6′_ = 5.3 Hz, 1H, H-7′endo); ^13^C{^1^H} NMR (100 MHz, MeOH-*d*_4_) δ 166.6 (C2), 153.0 (C4), 141.2 (C6), 110.1 (C5), 64.1 (C4′), 63.0 (C1″), 53.7 (C2′), 46.1 (C5′), 33.3 (C3′), 15.3 (C1′), 14.9 (C6′), 12.4 (*C*H_3_), 10.4 (C7′); HRMS (ESI+) *m/z*: [M + Na]^+^ calcd for C_13_H_18_N_2_O_4_Na 289.1164; found 289.1164.

### 19

*R_f_* = 0.36 (CH_2_Cl_2_:MeOH, 15:1); ^1^H NMR (400 MHz, MeOH-*d*_4_) δ 7.54 (q, *J*_6,CH3_ = 1.1 Hz, 1H, H-6), 5.37 (m, 1H, H-4′), 5.12 (dt, *J*_2′,3′ax_ = 6.6 Hz, *J*_2′,3′eq_ = *J*_2′,1′_ = 2.1 Hz, 1H, H-2′), 4.17 (m, 1H, H-1″a), 4.16–4.07 (m, 1H, H-1″b), 2.28 (ddd, *J*_gem_ = 18.2 Hz, J_3′ax,2′_ = 6.9 Hz, *J*_3′ax,4′_ = 2.4 Hz, 1H, H-3′ax), 2.18 (ddd, *J*_gem_ = 18.2 Hz, *J*_3′eq,4′_ = 5.5 Hz, *J*_3′eq,2′_ = 2.1 Hz, 1H, H-3′eq), 1.84 (d, *J*_CH3,6_ = 1.2 Hz, 3H, C*H*_3_), 1.72 (td, *J*_6′,7exo_ = *J*_6′,1′_ = 8.7 Hz, *J*_6′,7′endo_ = 4.8 Hz, 1H, H-6′), 1.50 (tdd, *J*_1′,7exo_ = *J*_1′,6′_ = 8.7 Hz, *J*_1′,7′endo_ = 4.8 Hz, *J*_1′,2′_ = 2.1 Hz, 1H, H-1′), 1.17 (td, *J*_7′exo,6′_ = *J*_7′exo,1′_ = 8.7 Hz, *J*_gem_ = 4.8 Hz, 1H, H-7′exo), 0.86 (q, *J*_gem_ = *J*_7′endo,6′_ = *J*_7′endo,1′_ = 4.8 Hz, 1H, H-7′endo); ^13^C{^1^H} NMR (100 MHz, MeOH-*d*_4_) δ 166.4 (C4), 153.0 (C2), 143.0 (C5′), 140.6 (C6), 114.2 (C4′), 110.4 (C5), 66.5 (C1″), 47.6 (C2′), 27.3 (C3′), 18.5 (C1′), 12.9 (C6′), 12.6 (*C*H_3_), 11.3 (C7′); HRMS (ESI+) *m/z*: [M + Na]^+^ calcd for C_13_H_16_N_2_O_3_Na 271.1059; found 271.1035.

### ((1′*R*,2′*R*,3′*R*,5′*S*,6′*S*)-3′-(Benzoyloxy)-5′-[6″-chloro-2″-(formylamino)-9″*H*-purin-9″-yl]bicyclo[4.1.0]hept-2′-yl)methyl benzoate (**22**)

A solution of the ammonium salt **17** (100 mg, 0.25 mmol), 4,6-dichloro-2,5-diformamidopyrimidine, **20** (64 mg, 0.25 mmol), and *N,N*-diisopropylethylamine (DIPEA, 175 μL, 1.0 mmol) in dry 1,4-dioxane (5 mL) was stirred under argon at RT for 12 h and then refluxed in an oil bath for 30 min. The solvent was evaporated to dryness under reduced pressure, and the residue was dissolved in EtOAc (7 mL), washed with water (5 mL) and brine (5 mL), dried (Na_2_SO_4_), filtered, and evaporated in vacuo. The crude was then dissolved in diethoxymethyl acetate, **21** (5 mL), and the solution was stirred at 140 °C in an oil bath under argon for 24 h. The mixture was cooled to RT and treated with MeOH (4 mL) and concentrated aqueous ammonia (0.5 mL) while stirring was continued. The solvent was evaporated to dryness, and the residue was dissolved in EtOAc (30 mL) and extracted with water (3 × 15 mL). The organic layer was dried (Na_2_SO_4_), filtered, and evaporated to dryness. The resultant yellow solid was purified by column chromatography (hexanes:EtOAc, 5:1 → 1:1) to give compound **22** (103 mg, 0.19 mmol, 76% overall yield) as a yellowish solid. *R_f_* = 0.11 (hexanes:EtOAc, 1:1); [α]_D_^20^ + 12.7 (*c* 0.79, CHCl_3_); ^1^H NMR (400 MHz, MeOH-*d*_4_) δ 9.55 (d, *J*_CHO,NH_ = 10.3 Hz, 1H, C*H*O), 8.94 (d, *J*_NH,CHO_ = 10.3 Hz, 1H, N*H*), 8.12 (s, 1H, H-8″), 8.10–8.05 (m, 2H, H-*orto*), 7.90–7.84 (m, 2H, H-*orto*), 7.60–7.52 (m, 2H, H-*para*), 7.48–7.34 (m, 4H, H-*meta*), 5.35–5.25 (m, 2H, H-1a, H-3′), 4.94 (ddd, *J*_5′,4′ax_ = 8.2 Hz, *J*_5′,4′eq_ = 6.7 Hz, *J*_5′,6′_ = 1.6 Hz, 1H, H-5′), 4.62 (dd, *J*_gem_ = 10.7 Hz, *J*_1b,2′_ = 5.5 Hz, 1H, H-1b), 2.80 (dtd, *J*_2′,3′_ = 8.3 Hz, *J*_2′,1b_ = *J*_2′,1a_ = 5.5 Hz, *J*_2′,1′_ = 2.4 Hz, 1H, H-2′), 2.66 (dt, *J*_gem_ = 13.9 Hz, *J*_4′ax,5′_ = *J*_4′ax,3′_ = 8.2 Hz, 1H, H-4′ax), 2.11 (ddd, *J*_gem_ = 13.9 Hz, *J*_4′eq,5′_ = 6.7 Hz, *J*_4′eq,3′_ = 3.5 Hz, 1H, H-4′eq), 1.53 (tdd, *J*_1′,7′exo_ = *J*_1′,6′_ = 8.5 Hz, *J*_1′,7′endo_ = 5.7 Hz, *J*_1′,2′_ = 2.4 Hz, 1H, H-1′), 1.33 (tdd, *J*_6′,7′exo_ = *J*_6′,1′_ = 8.5 Hz, *J*_6′,7′endo_ = 5.7 Hz, *J*_6′,5′_ = 1.6 Hz, 1H, H-6′), 1.10 (td, *J*_7′exo,6′_ = *J*_7′exo,1′_ = 8.5 Hz, *J*_gem_ = 5.8 Hz, 1H, H-7′exo), 0.59 (q, *J*_gem_ = *J*_7′endo,6′_ = *J*_7′endo,1′_ = 5.7 Hz, 1H, H-7′endo); ^13^C{^1^H} NMR (100 MHz, MeOH-*d*_4_) δ 167.2 (*C*O), 165.7 (*C*O), 163.0 (*C*HO), 152.2/152.0/151.8 (C-4″, C-2″, C-6″), 143.7 (C-8), 133.7 (C*para*), 133.5 (C*para*), 129.9 (C*orto*), 129.7 (C*ipso*), 129.5 (C*orto*), 129.4 (C*ipso*), 129.3 (C5″), 128.6 (C*meta*), 128.6 (C*meta*), 67.4 (C3′), 65.0 (C1), 51.6 (C5′), 36.3 (C2′), 29.5 (C4′), 14.3 (C1′), 14.2 (C6′), 10.3 (C7′); IR (ATR) 3276, 2922, 2360, 1700, 1601, 1574, 1266, 1110, 749 cm^–1^; HRMS (ESI+) *m/z*: [M + H]^+^ calcd for C_28_H_25_ClN_5_O_5_ 546.1544; found 546.1546.

### 2-Amino-9-[(1′*S*,2′*S*,4′*R*,5′*S*,6′*S*)-4′-hydroxy-5′-(hydroxymethyl) Bicyclo[4.1.0]hept-2′-yl]-1,9-dihydro-6*H*-purin-6-one (**1b**)

A solution of **22** (100 mg, 0.18 mmol) in 80% HCO_2_H (4.5 mL) was stirred at reflux in an oil bath for 2 h. The solution was cooled at RT, and the solvent was evaporated under reduced pressure. The residue was dissolved in a NH_3_ solution in MeOH (7 M, 20 mL) and stirred at RT for 3 days. Then, the mixture was concentrated under reduced pressure and purified by column chromatography (CH_2_Cl_2_:MeOH, 10:1 → 5:1 → 1:1) to provide guanine compound **1b** (30 mg, 0.10 mmol, 57% yield) as a pale white solid. *R_f_* = 0.09 (CH_2_Cl_2_:MeOH, 15:1); [α]_D_^20^ + 26.5 (*c* 0.48, MeOH); ^1^H NMR (400 MHz, MeOH-*d*_4_) δ 7.99 (s, 1H, H-8), 4.73 (td, *J*_2′,3′ax_ = *J*_2′,3′eq_ = 6.5 Hz, *J*_2′,1′_ = 1.6 Hz, 1H, H-2′), 3.94 (dd, *J*_gem_ = 10.7 Hz, *J*_1″a,5′_ = 6.5 Hz, 1H, H-1″a), 3.91–3.83 (m, 2H, H-1″b, H-4′), 2.06 (dt, *J*_gem_ = 14.1 Hz, *J*_3′ax,2′_ = *J*_3′ax,4′_ = 6.5 Hz, 1H, H-3′ax), 1.87 (tdd, *J*_5′,1″a_ = *J*_5′,1″b_ = 6.7 Hz, *J*_5′,3′ax_ = 6.5 Hz, *J*_5′,3′eq_ = 3.0 Hz, 1H, H-5′), 1.76 (ddd, *J*_gem_ = 14.1 Hz, *J*_3′eq,2′_ = 6.5 Hz, *J*_3′eq,4′_ = 3.0 Hz, 1H, H-3′eq), 1.31–1.13 (m, 2H, H-1′, H-6′), 0.96 (td, *J*_7′exo,1′_ = *J*_7′exo,6′_ = 9.2 Hz, *J*_gem_ = 5.0 Hz, 1H, H-7′exo), 0.28 (q, *J*_gem_ = *J*_7′endo,1′_ = *J*_7′endo,6′_ = 5.4 Hz, 1H, H-7′endo); ^13^C{^1^H} NMR (100 MHz, MeOH-*d*_4_) δ 158.0 (C6), 153.6 (C2), 151.3 (C4), 138.1 (C8), 115.9 (C5), 64.4 (C4′), 63.0 (C1″), 49.2 (C2′), 41.4 (C5′), 33.3 (C3′), 14.0 (C1′), 12.2 (C6′), 10.1 (C7′); HRMS (ESI+) *m/z*: [M + H]^+^ calcd for C_13_H_18_N_5_O_3_ 292.1410; found 292.1410.

### Methyl 1-{(1′*S*,2′*S*,4′*R*,5′*R*,6′*R*)-4′-(Benzoyloxy)-5′-[(benzoyloxy)methyl]-bicyclo[4.1.0]hept-2′-yl}-1*H*-1,2,3-triazole-4-carboxylate (**24**)

The benzyl protected azido alcohol **6** (200 mg, 0.511 mmol) was suspended in a 2:1 mixture of acetonitrile and water (8 mL), and methyl propiolate, **23** (70 μL, 0.767 mmol), was added. Then, a 1 M solution of sodium ascorbate in water (205 μL, 0.205 mmol, 40 mol %) and a 1 M of CuSO_4_·5H_2_O in water (102 μL, 0.10 mmol, 20 mol %) were sequentially added. The round-bottom flask was wrapped with aluminum foil, and the reaction mixture was stirred 16 h at RT. Then, the mixture was filtered through a short pad of Celite and washed with EtOAc. The solvent was evaporated under reduced pressure and purified by column chromatography (hexanes:EtOAc, 4:1 → 1:1) to afford triazole compound **24** (192 mg, 0.40 mmol, 79% yield) as a white solid. *R_f_* = 0.27 (hexanes:EtOAc, 1:1); [α]_D_^20^–115 (*c* 0.40, CHCl_3_); ^1^H NMR (400 MHz, CDCl_3_) δ 8.28 (s, 1H, H-5), 7.98 (dd, *J_orto,meta_* = 8.4 Hz, *J_orto,para_* = 1.3 Hz, 2H, H-*orto*), 7.78 (dd, *J_orto,meta_* = 8.4 Hz, *J_orto,para_* = 1.3 Hz, 2H, H-*orto*), 7.58–7.48 (m, 2H, H-*para*), 7.46–7.33 (m, 4H, H-*meta*), 5.30 (ddd, *J*_4′,3′ax_ = 5.8 Hz, *J*_4′,5′_ = 4.4 Hz, *J*_4′,3′eq_ = 2.9 Hz, 1H, H-4′), 5.14 (ddd, *J*_2′,3′eq_ = 6.2 Hz, *J*_2′,3′ax_ = 4.8, *J*_2′,1′_ = 1.6 Hz, 1H, H-2′), 4.72 (dd, *J*_gem_ = 10.9 Hz, *J*_1″a,5′_ = 7.3 Hz, 1H, H-1″a), 4.54 (dd, *J*_gem_ = 10.9 Hz, *J*_1″b,5′_ = 8.1 Hz, 1H, H-1″b), 3.77 (s, 3H, OC*H*_3_), 2.78 (ddd, *J*_gem_ = 15.3 Hz, *J*_3′ax,4′_ = 5.8 Hz, *J*_3′ax,2′_ = 4.8 Hz, 1H, H-3′ax), 2.49 (tdd, *J*_5′,1″a_ = *J*_5′,1″b_ = 7.3 Hz, *J*_5′,4′_ = 4.4 Hz, *J*_5′,6′_ = 2.8 Hz, 1H, H-5′), 2.08 (ddd, *J*_gem_ = 15.3 Hz, *J*_3′eq,2′_ = 6.2 Hz, *J*_3′eq,4′_ = 2.9 Hz, 1H, H-3′eq), 1.67–1.60 (m, 1H, H-1′), 1.35 (tdd, *J*_6′,7′exo_ = *J*_6′,1′_ = 9.3 Hz, *J*_6′,7′endo_ = 5.5 Hz, *J*_6′,5′_ = 2.8 Hz, 1H, H-6′), 1.23 (td, *J*_7′exo,6′_ = *J*_7′exo,1′_ = 9.3 Hz, *J*_gem_ = 5.5 Hz, 1H, H-7′exo), 0.50 (q, *J*_gem_ = *J*_7′endo,6′_ = *J*_7′endo,1′_ = 5.5 Hz, 1H, H-7′endo); ^13^C{^1^H} NMR (100 MHz, CDCl_3_) δ 166.5 (*C*O), 165.5 (*C*O), 160.8 (*C*(*O*)OCH_3_), 139.3 (C4), 133.3 (C*para*), 133.1 (C*para*), 129.8 (C*ipso*), 129.7 (C*orto*), 129.5 (C*orto*), 129.4 (C*ipso*), 128.6 (C*meta*), 128.5 (C*meta*), 126.8 (C5), 66.5 (C4′), 64.8 (C1″), 54.8 (C2′), 52.0 (O*C*H_3_), 38.0 (C5′), 30.7 (C3′), 14.1 (C1′), 11.4 (C7′), 11.2 (C6′); IR (ATR) 3007, 2164, 2111, 1712, 1601, 1265, 1175, 752, 710 cm-1; HRMS (ESI+) *m/z*: [M + H]^+^ calcd for C_26_H_26_N_3_O_6_ 476.1822; found 476.1821.

### 1-[(1′*S*,2′*S*,4′*R*,5′*R*,6′*R*)-4′-Hydroxy-5′-(hydroxymethyl)bicyclo[4.1.0]hept-2′-yl]-1*H*-1,2,3-triazole-4-carboxamide (**3a**)

The ester-triazole **24** (130 mg, 0.27 mmol) was dissolved in a solution of NH_3_ in MeOH (7 M, 6 mL) in a sealed flask and was stirred for 2 days at RT. Then, the mixture was concentrated under reduced pressure and purified by column chromatography (CH_2_Cl_2_:MeOH, 20:1 → 15:1 → 10:1) to provide carboxamide **3a** (61 mg, 0.24 mmol, 92% yield) as a white solid. *R_f_* = 0.15 (hexanes:EtOAc, 1:1); [α]_D_^20^–78.6 (*c* 0.79, CHCl_3_); ^1^H NMR (400 MHz, MeOH-*d*_4_) δ 8.63 (s, 1H, H-5), 5.07 (ddd, *J*_2′,3′eq_ = 6.1 Hz, *J*_2′,3′ax_ = 5.5 Hz, *J*_2′,1′_ = 1.6 Hz, 1H, H-2′), 3.96–3.86 (m, 2H, H-4′, H-1″a), 3.81 (dd, *J*_gem_ = 10.7 Hz, *J*_1″b,5′_ = 6.7 Hz, 1H, H-1″b), 2.12 (ddd, *J*_gem_ = 14.5 Hz, *J*_3′ax,4′_ = 6.3 Hz, *J*_3′ax,2′_ = 5.5 Hz, 1H, H-3′ax), 1.93 (ddd, *J*_gem_ = 14.5 Hz, *J*_3′eq,2′_ = 6.1 Hz, *J*_3′eq,4′_ = 3.0 Hz, 1H, H-3′eq), 1.83 (tt, *J*_5′,1″a_ = *J*_5′,1″b_ = 6.7 Hz, *J*_5′,4′_ = *J*_5′,6′_ = 3.5 Hz, 1H, H-5′), 1.36–1.26 (m, 1H, H-1′), 1.14 (tdd, *J*_6′,7′exo_ = *J*_6′,1′_ = 8.9 Hz, *J*_6′,7′endo_ = 5.4 Hz, *J*_6′,5′_ = 3.5 Hz, 1H, H-6′), 1.01 (td, *J*_7′exo,6′_ = *J*_7′exo,1′_ = 8.9 Hz, *J*_gem_ = 5.4 Hz, 1H, H-7′exo), 0.33 (q, *J*_gem_ = *J*_7′endo,6′_ = *J*_7′endo,1′_ = 5.4 Hz, 1H, H-7′endo); ^13^C{^1^H} NMR (100 MHz, MeOH-*d*_4_) δ 165.0 (*C*(*O*)NH_2_), 143.1 (C4), 127.4 (C5), 65.1 (C4′), 64.4 (C1″), 57.2 (C2′), 43.1 (C5′), 35.1 (C3′), 15.4 (C1′), 12.8 (C6′), 11.5 (C7′); IR (ATR) 3339, 3195, 2886, 1661, 1603, 1289, 1046, 855, 721 cm^–1^; HRMS (ESI+) *m/z*: [M + Na]^+^ calcd for C_11_H_16_N_4_O_3_Na 275.1120; found 275.1121.

### 5-Amino-1-[(1′*S*,2′*S*,4′*R*,5′*R*,6′*R*)-4′-hydroxy-5′-(hydroxymethyl)bicyclo-[4.1.0]hept-2′-yl]-1*H*-1,2,3-triazole-4-carboxamide (**3b**)

To a solution of 2-cyanoacetamide, **25** (35 mg, 0.42 mmol), in dry DMSO (500 μL) at RT, K_2_CO_3_ (54 mg, 0.39 mmol) under an argon atmosphere was added. The mixture was stirred at the same temperature for 1 h. After this time, a DMSO solution (500 μL) of **6** (50 mg, 0.13 mmol) was added and the stirring was continued for 2 d at 50 °C in an oil bath. After evaporation of the solvent, the solid residue was purified by column chromatography (CH_2_Cl_2_:MeOH, 15:1 → 10:1) to give **3b** (19 mg, 0.07 mmol, 55% yield) as whitish solid. *R_f_* = 0.1 (CH_2_Cl_2_:MeOH, 10:1); Mp 210–213 °C (from MeOH-CH_2_Cl_2_); [α]_D_^20^–35.6 (*c* 0.64, MeOH); ^1^H NMR (400 MHz, MeOH-*d*_4_) δ 4.72 (td, *J*_2′,3′ax_ = *J*_2′,3′eq_ = 7.5 Hz, *J*_2′,1′_ = 1.9 Hz, 1H, H-2′), 3.95 (dd, *J*_gem_ = 10.6 Hz, *J*_1_″_a,5′_ = 6.4 Hz, 1H, H-1″a), 3.91–3.80 (m, 2H, H-4′, H-1″b), 2.12 (dt, *J*_gem_ = 13.7 Hz, *J*_3′ax,2′_ = *J*_3′ax,4′_ = 8.2 Hz, 1H, H-3′ax), 1.97–1.87 (m, 2H, H-3′eq, H-5′), 1.29–1.17 (m, 2H, H-1′, H-6′), 0.95 (td, *J*_7′exo,1′_ = J_7′exo,6′_ = 9.2, *J*_gem_ = 5.4 Hz, 1H, H-7′exo), 0.32 (q, *J*_gem_ = *J*_7′endo,1′_ = J_7′endo,6′_ = 5.4 Hz, 1H, H-7′endo); ^13^C{^1^H} NMR (100 MHz, MeOH-*d*_4_) δ 167.1 (*C*O), 146.0 (C5), 123.3 (C4), 65.7 (C4′), 63.8 (C1″), 54.2 (C2′), 42.3 (C5′), 34.4 (C3′), 14.4/14.2 (C1′/C6′), 11.2 (C7′); IR (ATR) 3308, 3174, 1662, 1633, 1561, 1517, 1226, 1096, 1021, 877, 784 cm^–1^; HRMS (ESI+) *m/z*: [M + Na]^+^ calcd for C_11_H_17_N_5_O_3_Na 290.1229; found 290.1229.

### (1*R*,2*R*,3*R*,5*S*,6*S*)-5-Azido-2-(hydroxymethyl)bicyclo[4.1.0]heptan-3-ol (**26**)

Compound **6** (320 mg, 0.82 mmol) was dissolved in a solution of NH_3_ solution in MeOH (7 M, 50 mL) in a sealed flask, and the solution was stirred for 3 days at RT. Then, the mixture was concentrated under reduced pressure and purified by column chromatography (CH_2_Cl_2_:MeOH, 30:1 → 20:1 → 10:1) to provide the deprotected azide compound **26** (128 mg, 0.70 mmol, 85% yield) as a white solid. *R_f_* = 0.32 (CH_2_Cl_2_:MeOH, 20:1); [α]_D_^20^–46.3 (*c* 1.0, MeOH); ^1^H NMR (400 MHz, MeOH-*d*_4_) δ 3.90–3.76 (m, 3H, H-3, H-5, H-1′a), 3.68 (dd, *J*_gem_ = 10.6 Hz, *J*_1′b,2_ = 7.1 Hz, 1H, H-1′b), 1.82–1.67 (m, 2H, H-4ax, H-2), 1.62 (ddd, *J*_gem_ = 14.3 Hz, *J*_4eq,5_ = 5.8 Hz, *J*_4eq,3_ = 3.0 Hz, 1H, H-4 eq), 1.13–1.03 (m, 1H, H-6), 0.95 (tdd, *J*_1,6_ = *J*_1,7exo_ = 9.1 Hz, *J*_1,7endo_ = 5.2 Hz, *J*_1,2_ = 2.9 Hz, 1H, H-1), 0.87 (td, *J*_7exo,1_ = *J*_7exo,6_ = 9.1 Hz, *J*_gem_ = 4.7 Hz, 1H, H-7exo), 0.11 (q, *J*_gem_ = *J*_7endo,1_ = *J*_7endo,6_ = 5.2 Hz, 1H, H-7endo); ^13^C{^1^H} (101 MHz, MeOH-*d*_4_) δ 65.5 (C3), 64.5 (C1′), 57.5 (C5), 43.2 (C2), 33.4 (C4), 14.9 (C6), 12.7 (C1), 11.0 (C7); HRMS (ESI+) *m/z*: [M + Na]^+^ calcd for C_8_H_13_N_3_O_2_Na 206.0905; found 206.0904.

### General Procedure for the CuAAC under MW Irradiation

To a solution of azide **26** in a 1:1 mixture of water and *t-*BuOH (10 mL/mmol) in a glass vial equipped with a magnetic stirring bar, copper powder (80 mol %), a 1 M solution of copper sulfate in water (20 mol %), and finally the alkyne (1.05 eq) were added. The vial was sealed with a Teflon crimp top, and the reaction mixture was irradiated under MW at 125 °C. Upon completion of the reaction, the vial was cooled to 50 °C by air jet cooling before opening. The mixture was filtered over a plug of Celite (rinsed with MeOH), and the filtrate was evaporated under reduced pressure.

### (1*R*,2*R*,3*R*,5*S*,6*S*)-2-(Hydroxymethyl)-5-(4″-phenyl-1*H*-1″,2″,3″-triazol-1-yl)bicyclo[4.1.0]heptan-3-ol (**3c**)

By following the general procedure, the title compound was prepared from azide **26** (19 mg, 0.11 mmol) and phenylacetylene, **27c** (12 μL, 0.110 mmol), after 2 min of MW irradiation. Purification by column chromatography (EtOAc) delivered compound **3c** (29 mg, 0.102 mmol, 97% yield) as a white solid. *R_f_* = 0.21 (EtOAc); [α]_D_^20^–52 (*c* 0.8, CHCl_3_); ^1^H NMR (400 MHz, MeOH-*d*_4_) δ 8.52 (s, 1H, H-5″), 7.86–7.79 (m, 2H, H-*orto*), 7.46–7.41 (m, 2H, H-*meta*), 7.37–7.31 (m, 1H, H-*para*), 5.02 (td, *J*_5,4ax_ = *J*_5,4eq_ = 6.6 Hz, *J*_5,6_ = 1.8 Hz, 1H, H-5), 4.02–3.91 (m, 2H, H-3, H-1′a), 3.84 (dd, *J*_gem_ = 10.7 Hz, *J*_1′b,2_ = 6.8 Hz, 1H, H-1′b), 2.16 (dt, *J*_gem_ = 13.9 Hz, *J*_4ax,3_ = *J*_4ax,5_ = 7.0 Hz, 1H, H-4ax), 2.00 (ddd, *J*_gem_ = 14.2 Hz, *J*_4eq,5_ = 6.7 Hz, *J*_4eq,3_ = 3.3 Hz, 1H, H-4 eq), 1.90 (tt, *J*_2,1_′ = 6.8 Hz, *J*_2,3_ = *J*_2,1_ = 3.6 Hz, 1H, H-2), 1.39–1.31 (m, 1H, H-6), 1.20 (tdd, *J*_1,6_ = *J*_1,7exo_ = 8.7 Hz, *J*_1,7endo_ = 5.4 Hz, *J*_1,2_ = 3.2 Hz, 1H, H-1), 1.01 (td, *J*_7exo,6_ = *J*_7exo,1_ = 9.2 Hz, *J*_gem_ = 5.4 Hz, 1H, H-7exo), 0.37 (q, *J*_gem_ = *J*_7endo,6_ = *J*_7endo,1_ = 5.4 Hz, 1H, H-7endo), ^13^C{^1^H} NMR (100 MHz, MeOH-*d*_4_) δ 148.5 (C4″), 131.9 (C*ipso*), 130.0 (C*meta*), 129.2 (C*para*), 126.6 (C*orto*), 121.7 (C5″), 65.5 (C3), 64.3 (C1′), 57.6 (C5), 43.0 (C2), 35.6 (C4), 15.6 (C6), 13.5 (C1), 11.4 (C7); HRMS (ESI+) *m/z*: [M + H]^+^ calcd for C_16_H_20_N_3_O_2_ 286.1556, found 286,1555.

### (1*R*,2*R*,3*R*,5*S*,6*S*)-2-(Hydroxymethyl)-5-[4″-(4‴-propylphenyl)-1″*H*-1″,2″,3″-triazol-1″-yl]bicyclo[4.1.0]heptan-3-ol (**3d**)

By following the general procedure, the title compound was prepared from azide **26** (20 mg, 0.11 mmol) and 1-ethynyl-4-propylbenzene, **27d** (18 μL, 0.115 mmol), after 10 min of MW irradiation. Purification by column chromatography (EtOAc → EtOAc:MeOH, 95:5) afforded compound **3d** (33 mg, 0.10 mmol, 96% yield) as a white solid. *R_f_* = 0.20 (EtAOc); [α]_D_^20^–25 (*c* 0.45, CHCl_3_); ^1^H NMR (400 MHz, MeOH-*d*_4_) δ 8.47 (s, 1H, H-5″), 7.72 (d, *J*_2‴/6‴,3‴/5‴_ = 8.5 Hz, 2H, H-2‴/6‴), 7.25 (d, *J*_3‴/5‴,2‴/6‴_ = 8.5 Hz, 2H, H-3‴/5‴), 5.00 (td, *J*_5,4ax_ = *J*_5,4eq_ = 6.6 Hz, *J*_5,6_ = 1.8 Hz, 1H, H-5), 4.00–3.90 (m, 2H, H-3, H-1′a), 3.84 (dd, *J*_gem_ = 10.7 Hz, *J*_1′b,2_ = 6.8 Hz, 1H, H-1′b), 2.62 (t, *J*_1⁗,2⁗_= 7.4 Hz, 1H, H-1⁗), 2.21 – 2.10 (m, 1H, H-4ax), 1.99 (ddd, *J*_gem_ = 14.1 Hz, *J*_4eq,5_ = 6.7 Hz, *J*_4eq,3_ = 3.3 Hz, 1H, H-4eq), 1.94 – 1.86 (m, 1H, H-2), 1.67 (h, *J*_2⁗,1⁗_= *J*_2⁗,3⁗_= 7.4 Hz, 1H, H-2⁗), 1.32 (tdd, *J*_6,7exo_ = *J*_6,1_ = 9.2 Hz, *J*_6,7endo_ = 5.6 Hz, *J*_6,5_ = 1.9 Hz, 1H, H-6), 1.20 (tdd, *J*_1,7exo_ = *J*_1,6_ = 8.7 Hz, *J*_1,7endo_ = 5.4 Hz, *J*_1,2_ = 3.1 Hz, 1H, H-1), 1.01 (td, *J*_7exo,1_ = *J*_7exo,6_ = 9.2 Hz, *J*_gem_ = 5.4 Hz, 1H, H-7exo), 0.96 (t, *J*_3⁗,2⁗_= 7.4 Hz, 1H, H-3⁗), 0.36 (q, *J*_gem_= *J*_7endo,6_= *J*_7endo,1_= 5.4 Hz, 1H, H-7endo); ^13^C{^1^H} NMR (100 MHz, MeOH-*d*_4_) δ 148.6 (C4′), 144.1 (C4″), 130.0 (2C, C2″/C6″), 129.4 (C1″), 126.6 (2C, C3″/C5″), 121.4 (C5′), 65.5 (C3), 64.3 (C1⁗), 57.6 (C5), 42.9 (C2), 38.8 (C1‴), 35.6 (C4), 25.7 (C2‴), 15.6 (C6), 14.1 (C3‴), 13.5 (C1), 11.4 (C7); HRMS (ESI+) *m/z*: [M + H]^+^ calcd for C_19_H_26_N_3_O_2_ 328.2025; found 328.2027.

### (1*R*,2*R*,3*R*,5*S*,6*S*)-2-(Hydroxymethyl)-5-[4″-(hydroxymethyl)-1*H*-1″,2″,3″-triazol-1‴-yl]bicyclo[4.1.0]heptan-3-ol (**3e**)

By following the general procedure, the title compound was prepared from azide **26** (49 mg, 0.27 mmol) and propargyl alcohol, **27e** (16 μL, 0.278 mmol), after 1 min of MW irradiation. Purification by column chromatography (EtOAc:MeOH 95:5 → 90:10) furnished compound **3e** (51 mg, 0.21 mmol, 80% yield) as a white solid. *R_f_* = 0.1 (CH_2_Cl_2_:MeOH, 10:1); [α]_D_^20^ 62.9 (*c* 0.98, MeOH);^1^H NMR (400 MHz, MeOH-*d*_4_) δ 8.14 (s, 1H, H-5″), 4.96 (td, *J*_5,4ax_ = *J*_5,4eq_ = 6.8 Hz, *J*_5,6_ = 1.9 Hz, 1H, H-5), 4.68 (br s, 2H, H-1‴), 3.97–3.89 (m, 2H, H-3, H-1′a), 3.81 (dd, *J*_gem_ = 10.7 Hz, J_1′b,2_ = 6.8 Hz, 1H, H-1′b), 2.09 (dt, *J*_gem_ = 14.1 Hz, *J*_4ax,3_ = *J*_4ax,5_ = 6.8 Hz, 1H, H-4ax), 1.96 (ddd, *J*_gem_ = 14.1 Hz, *J*_4eq,5_ = 6.8 Hz, *J*_4eq,3_ = 3.4 Hz, 1H, H-4 eq), 1.88 (tt, *J*_2,1′_ = 6.8 Hz, *J*_2,3_ = *J*_2,1_ = 3.7 Hz, 1H, H-2), 1.28–1.21 (m, 1H, H-6), 1.17 (tdd, *J*_1,7exo_ = *J*_1,6_ = 8.7 Hz, *J*_1,7endo_ = 5.4, *J*_1,2_ = 3.7 Hz, 1H, H-1), 0.97 (td, *J*_7exo,1_ = *J*_7exo,6_ = 9.2 Hz, *J*_gem_ = 5.4 Hz, 1H, H-7exo), 0.34 (q, *J*_gem_ = *J*_7endo,1_ = *J*_7endo,6_ = 5.4 Hz, 1H, H-7endo); ^13^C{^1^H} NMR (100 MHz, MeOH-*d*_4_) δ 148.9 (C4″), 123.6 (C5″), 65.5 (C3), 64.3 (C1′), 57.5 (C5), 56.6 (C1‴), 42.9 (C4), 35.7 (C2), 15.6 (C6), 13.5 (C1), 11.3 (C7); HRMS (ESI+) *m/z*: [M + H]^+^ calcd for C_11_H_18_N_3_O_3_ 240.1348; found 240.1348.

## Supporting Information Available

^1^H and ^13^C{^1^H} NMR spectra of all new compounds and 2D NMR spectra for compounds **9**, **10**, **12**, **6**, **1a**, **2**, **19**, **22**, **24**, and **3a**–**e**; cytotoxicity and antiviral activity of compounds **1a**,**b**, **2**, **19**, and **3a**–**e**; and X-ray crystal structure and crystallographic table for compounds **1a**, **6**, and **12** (PDF)
