# Supplementary material for: Conformationally Locked Carbocyclic Nucleosides Built on a 4′-Hydroxymethyl-3′-hydroxybicyclo[4.1.0]heptane Template. Stereoselective Synthesis and Antiviral Activity
Source: J Org Chem. 2022 Oct 27;87(22):15166–77. doi: 10.1021/acs.joc.2c01661 (PMC9680032; doi:10.1021/acs.joc.2c01661)
Supplement: Supplementary file 1 — jo2c01661_si_001.pdf [file jo2c01661_si_001.pdf]

Supplementary Information for:

Conformationally Locked Carbocyclic Nucleosides built on a 4'-Hydroxymethyl-3'-Hydroxybicyclo[4.1.0]Heptane Template. Stereoselective Synthesis and Antiviral Activity

Sergio Jurado,† *Ona Illa,*† *Angel Álvarez-Larena,*‡ *Christophe Pannecouque,*§ *Félix Busqué**,† *and Ramon Alibés**,†

† Departament de Química, Universitat Autònoma de Barcelona, Bellaterra 08193, Barcelona, Spain,

‡ Servei de Difracció de Raigs X. Universitat Autònoma de Barcelona, Bellaterra 08193, Barcelona, Spain.

§ Department of Microbiology and Immunology, Laboratory of Virology and Chemotherapy, Rega Institute for Medical Research, KU Leuven, Herestraat 49, B-3000 Leuven, Belgium

ramon.alibes@uab.cat; felix.busque@uab.cat

Table of contents

^{1}H NMR spectra of compound **4** S4

^{1}H and $^{13}C\{^{1}H\}$ NMR spectra of compound **7** S5

^{1}H and $^{13}C\{^{1}H\}$ NMR spectra of compound **8** S6

^{1}H and $^{13}C\{^{1}H\}$ NMR spectra of compound **5** S7

^{1}H and $^{13}C\{^{1}H\}$ NMR spectra of compound **9** S8

NOESY experiment for compound **9** S9

^{1}H and $^{13}C\{^{1}H\}$ NMR spectra of compound **10** S10

NOESY experiment for compound **10** S11

^{1}H and $^{13}C\{^{1}H\}$ NMR spectra of compound **12** S12

NOESY and HSQC experiments for compound **12** S13

^{1}H and $^{13}C\{^{1}H\}$ NMR spectra of compound **13** S14

^{1}H and $^{13}C\{^{1}H\}$ NMR spectra of compound **14** S15

^{1}H and $^{13}C\{^{1}H\}$ NMR spectra of compound **15** S16

^{1}H and $^{13}C\{^{1}H\}$ NMR spectra of compound **6** S17

COSY and NOESY experiments for compound **6** S18

^{1}H and $^{13}C\{^{1}H\}$ NMR spectra of compound **16** S19

^{1}H and $^{13}C\{^{1}H\}$ NMR spectra of compound **17** S20

^{1}H and $^{13}C\{^{1}H\}$ NMR spectra of compound **1a** S21

COSY and HMBC experiments for compound **1a** S22

^{1}H and $^{13}C\{^{1}H\}$ NMR spectra of compound **2** S23

COSY and NOESY experiments for compound **2** S24

^{1}H and $^{13}C\{^{1}H\}$ NMR spectra of compound **19** S25

HSQC and HMBC experiments for compound **19** S26

^{1}H and $^{13}C\{^{1}H\}$ NMR spectra of compound **22** S27

COSY and NOESY experiments for compound **22** S28

^{1}H and $^{13}C\{^{1}H\}$ NMR spectra of compound **1b** S29

^{1}H and $^{13}C\{^{1}H\}$ NMR spectra of compound **24** S30

HSQC and HMBC experiments for compound **24** S31

^{1}H and $^{13}C\{^{1}H\}$ NMR spectra of compound **3a** S32

HSQC and HMBC experiments for compound **3a** S33

^{1}H and $^{13}C\{^{1}H\}$ NMR spectra of compound **3b** S34

HSQC and HMBC experiments for compound **3b** S35

^{1}H and $^{13}C\{^{1}H\}$ NMR spectra of compound **26** S36

^{1}H and $^{13}C\{^{1}H\}$ NMR spectra of compound **3c** S37

HSQC and HMBC experiments for compound **3c** S38

^{1}H and $^{13}C\{^{1}H\}$ NMR spectra of compound **3d** S39

HSQC and HMBC experiments for compound **3d** S40

^{1}H and $^{13}C\{^{1}H\}$ NMR spectra of compound **3e** S41

HSQC and HMBC experiments for compound **3e** S42

Cytotoxicity and antiviral activity (**Figures S1-S2**, **Tables S1-S5**) S43-S48

Figure S3. Thermal ellipsoid plot of compound **12** S49

Figure S4. Thermal ellipsoid plot of compound **6** S50

Figure S5. Thermal ellipsoid plot of compound **1a** S51

X-ray structure Determination S52

Table S6. Crystal data and structure refinement for compound **12** S53

Table S7. Crystal data and structure refinement for compound **6** S54

Table S8. Crystal data and structure refinement for compound **1a** S55

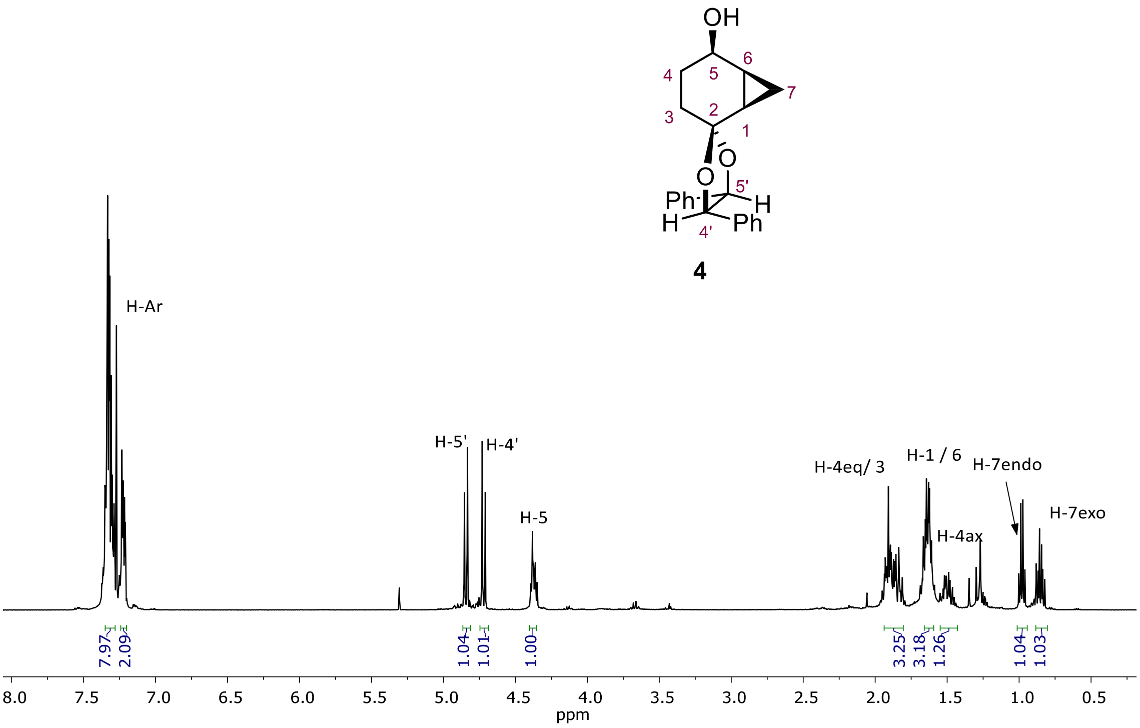

^{1}H NMR (400 MHz, $CDCl_3$)

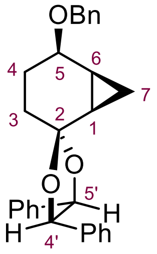

7

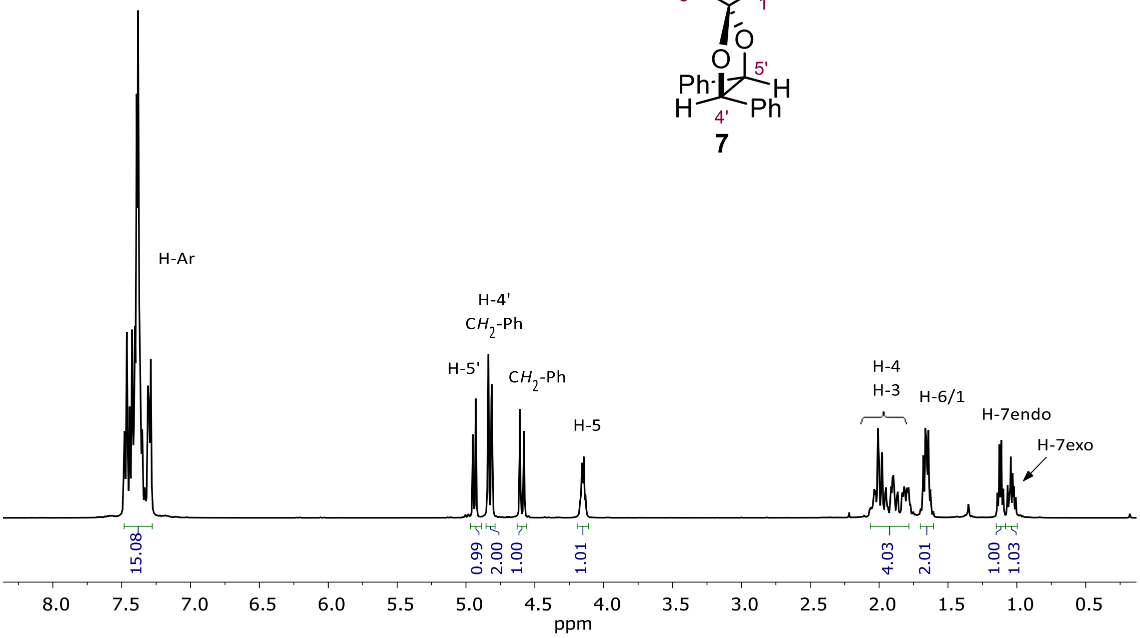

^{1}H NMR (400 MHz, $CDCl_3$)

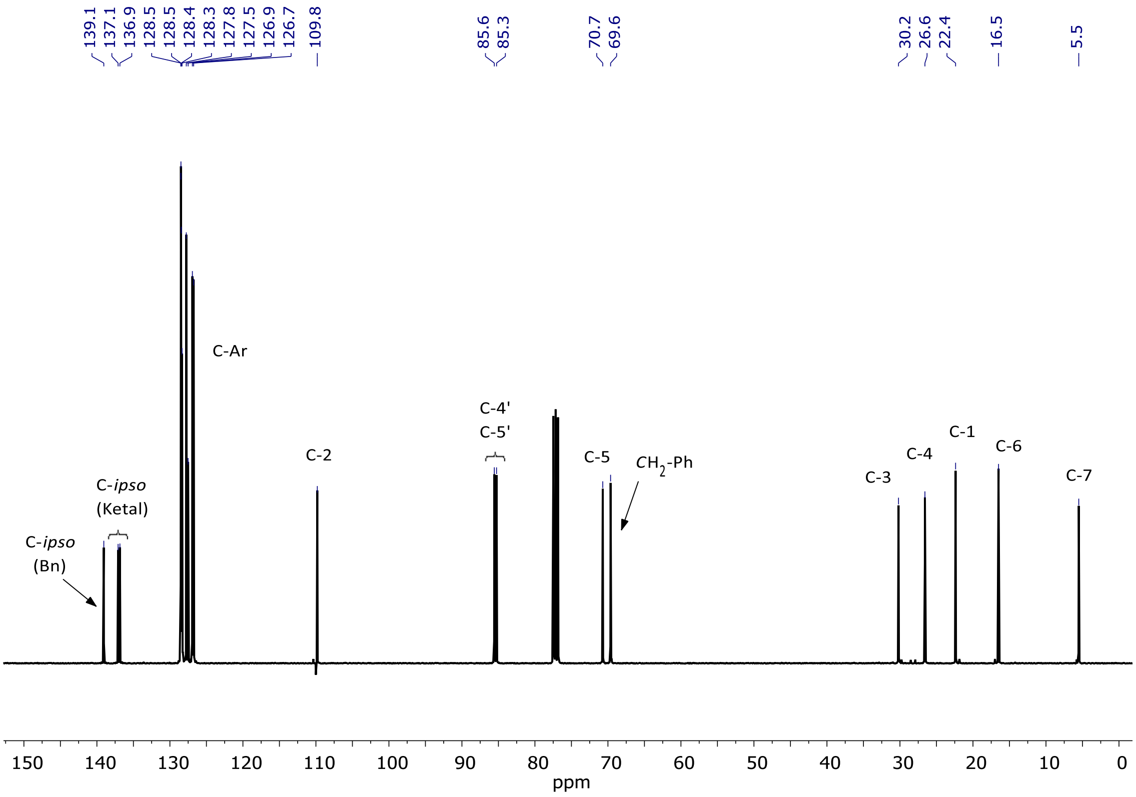

^{13}C{^{1}H} NMR (100 MHz, $CDCl_3$)

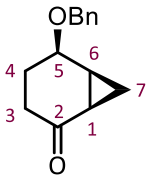

8

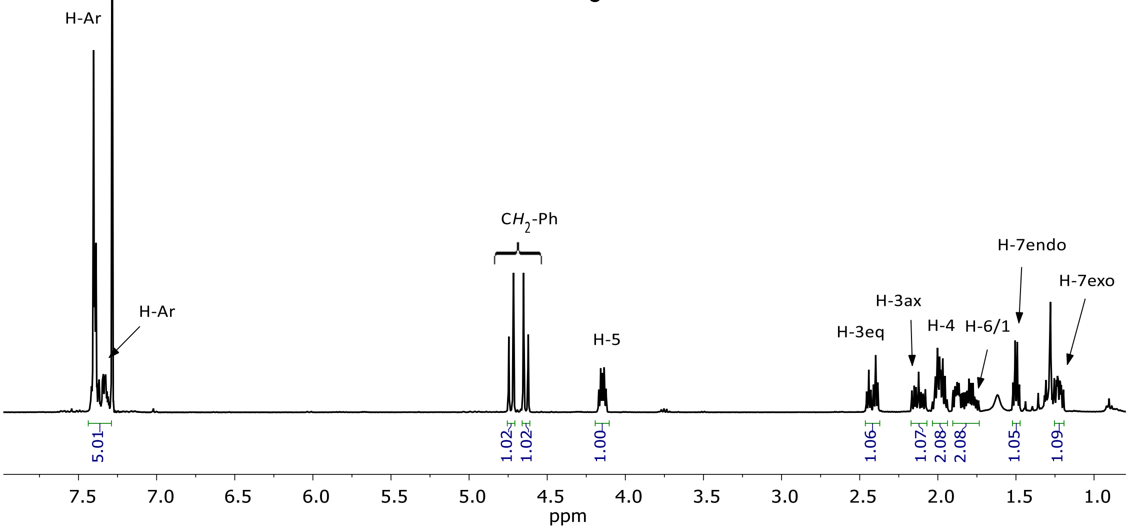

^{1}H NMR (400 MHz, $CDCl_3$)

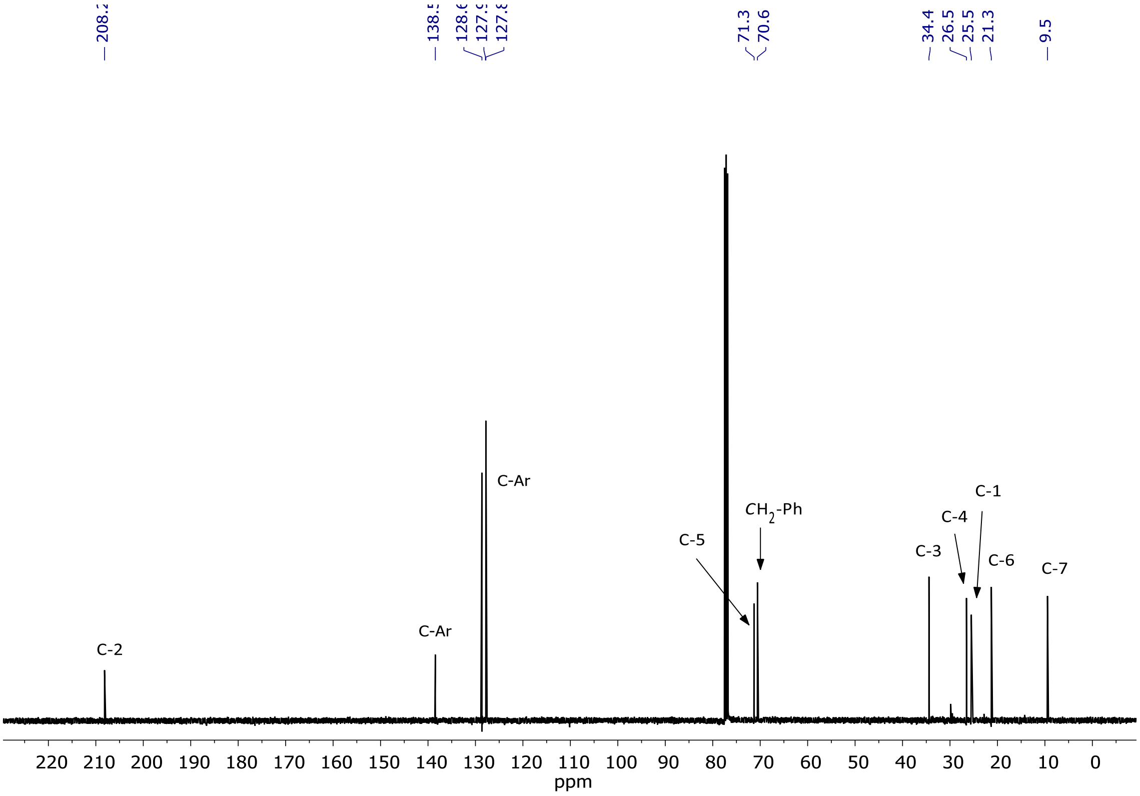

$^{13}C\{^{1}H\}$ NMR (100 MHz, $CDCl_3$)

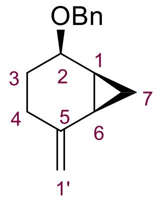

5

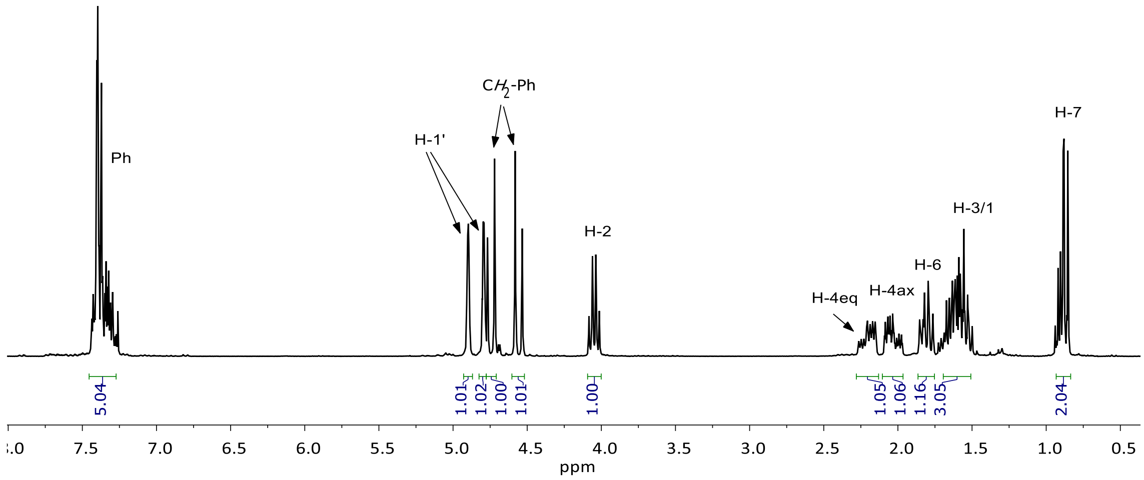

^{1}H NMR (400 MHz, $CDCl_3$)

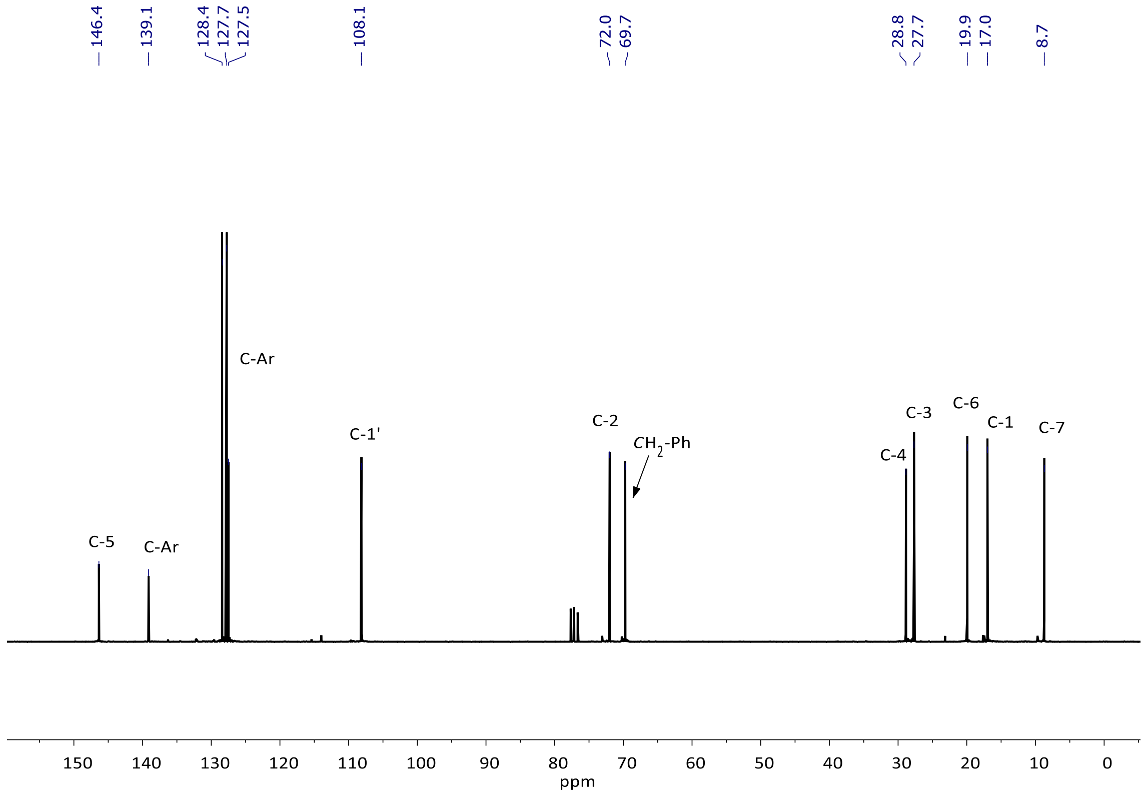

^{13}C{^{1}H} NMR (100 MHz, $CDCl_3$)

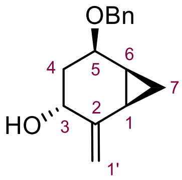

9

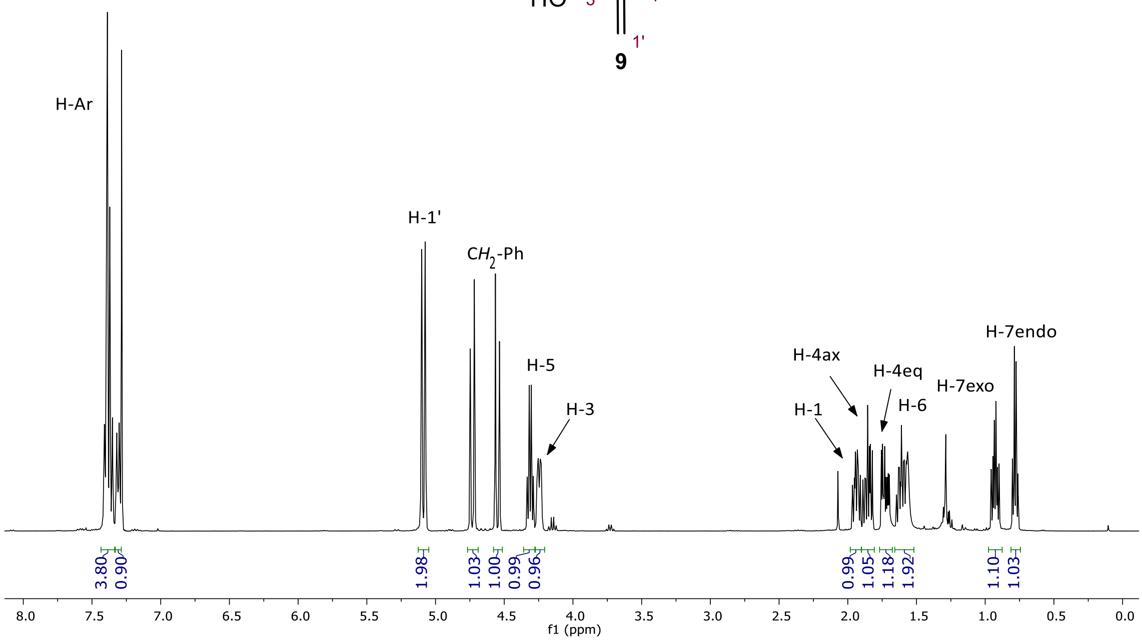

^{1}H NMR (400 MHz, $CDCl_3$)

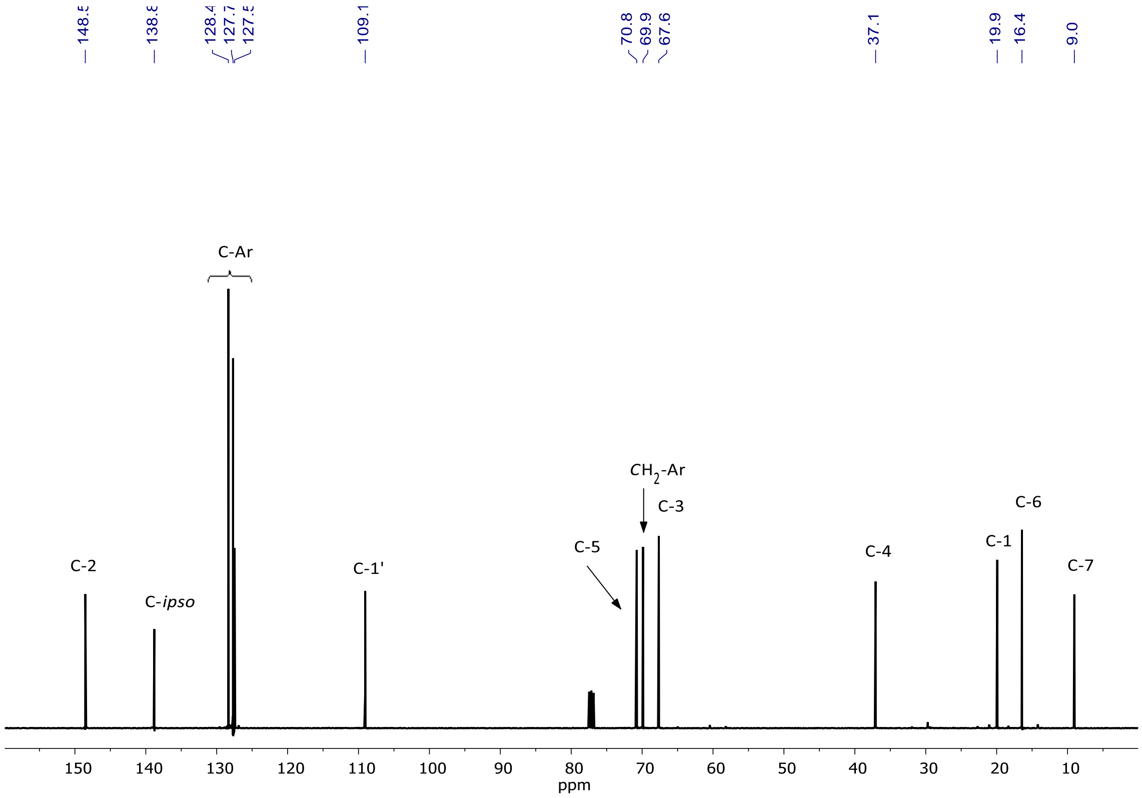

^{13}C{^{1}H} NMR (100 MHz, $CDCl_3$)

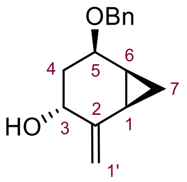

9

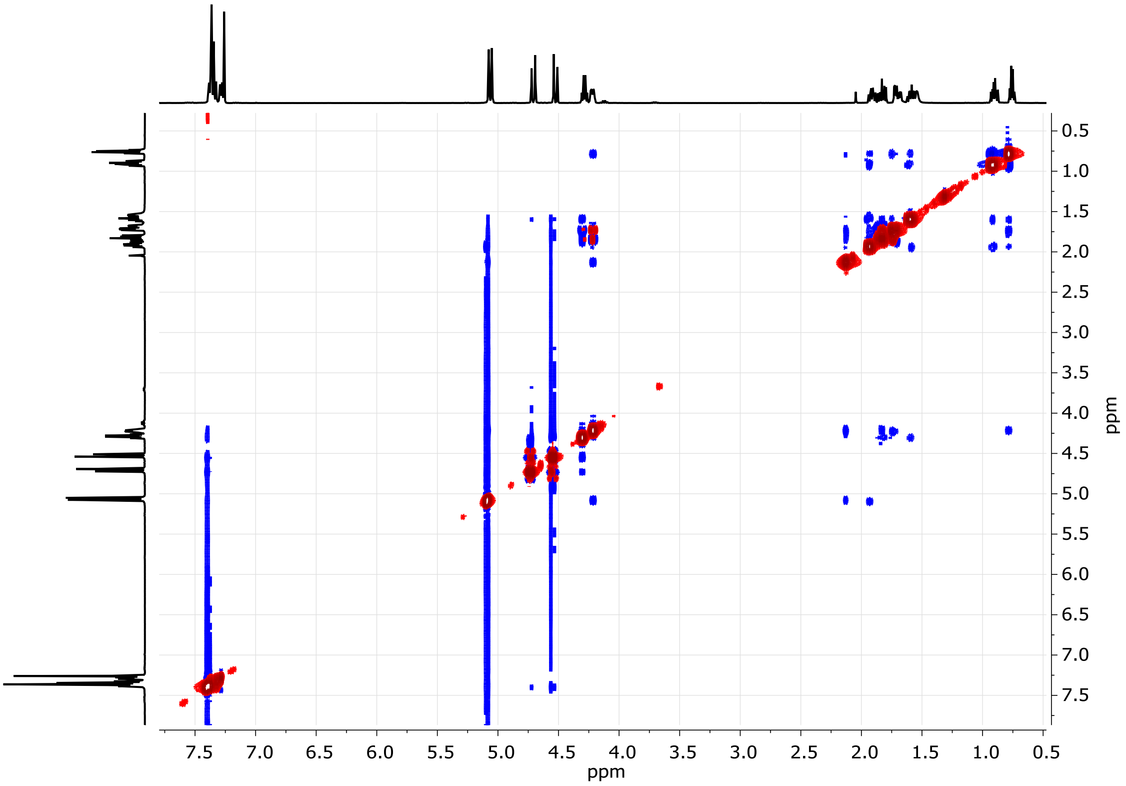

NOESY (400 MHz, $CDCl_3$)

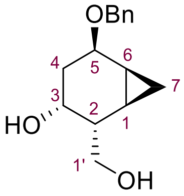

10

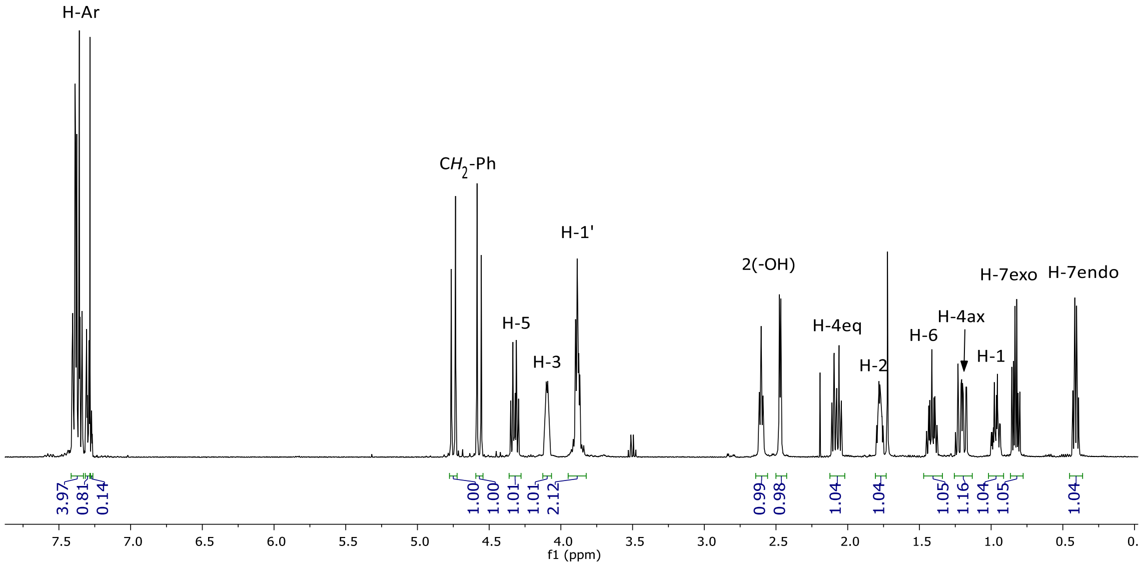

^{1}H NMR (400 MHz, $CDCl_3$)

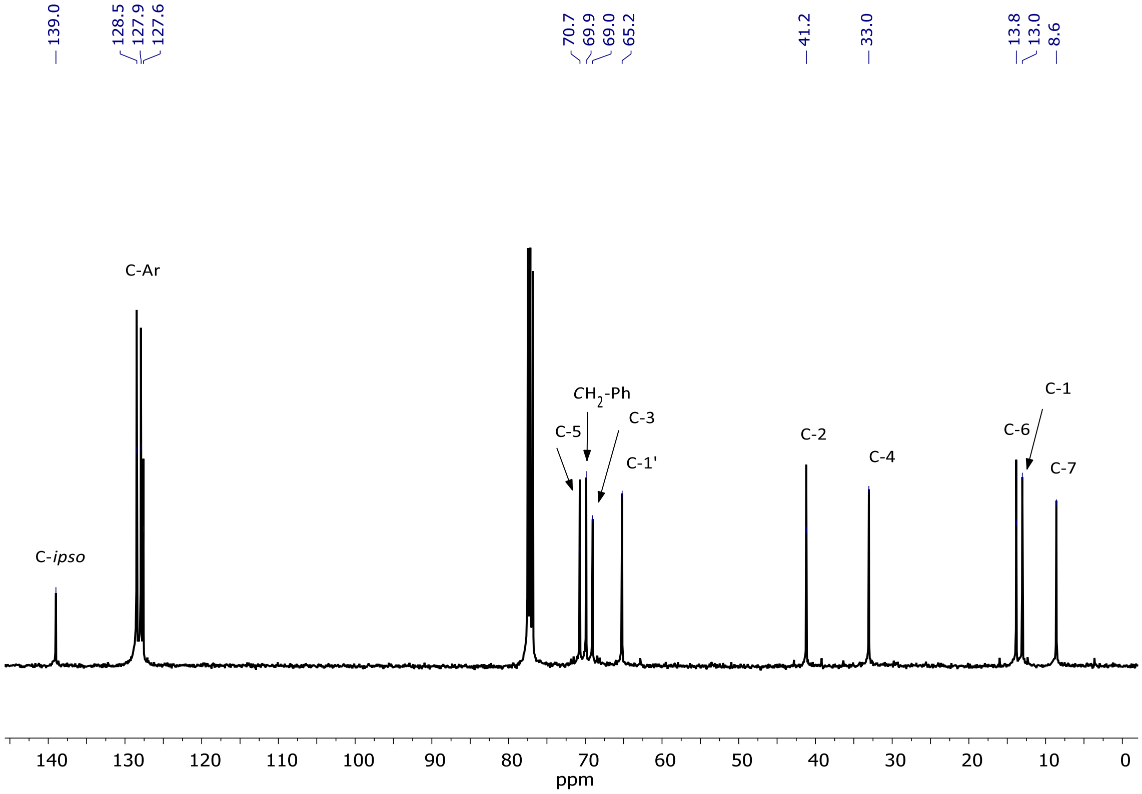

$^{13}C\{^{1}H\}$ NMR (100 MHz, $CDCl_3$)

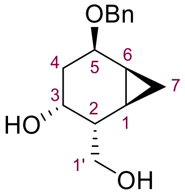

10

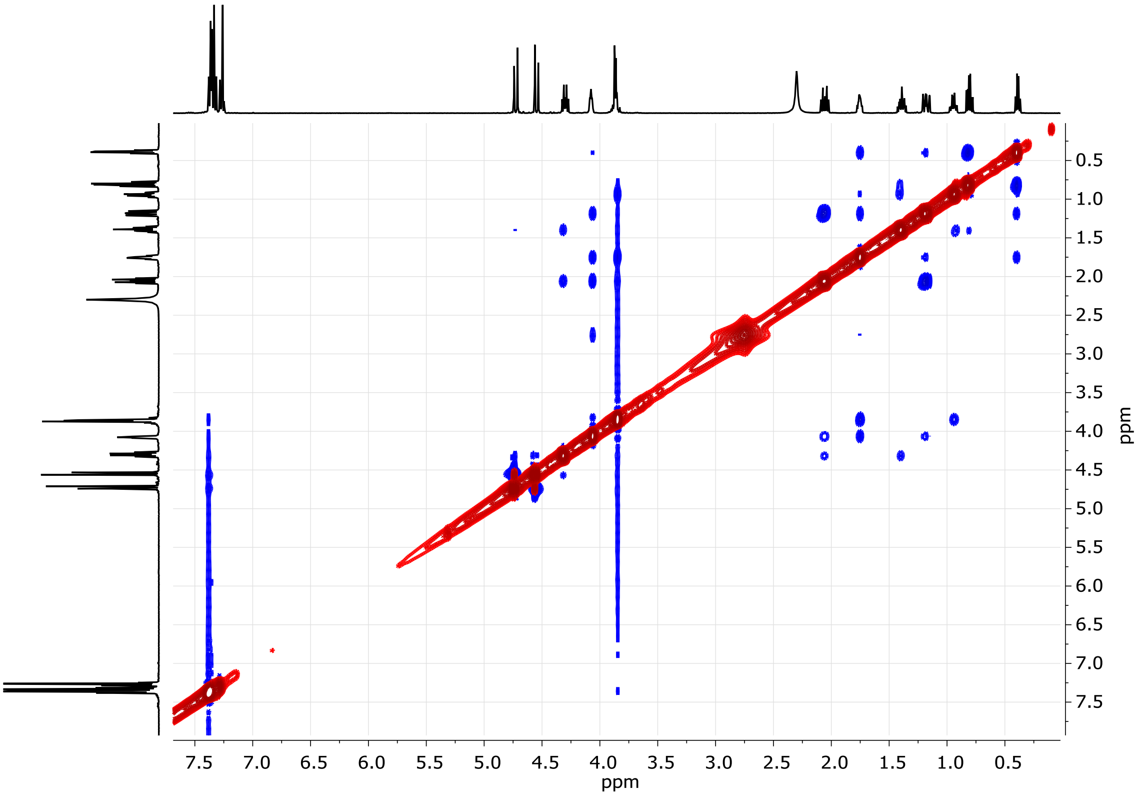

NOESY (400 MHz, $CDCl_3$)

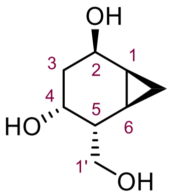

12

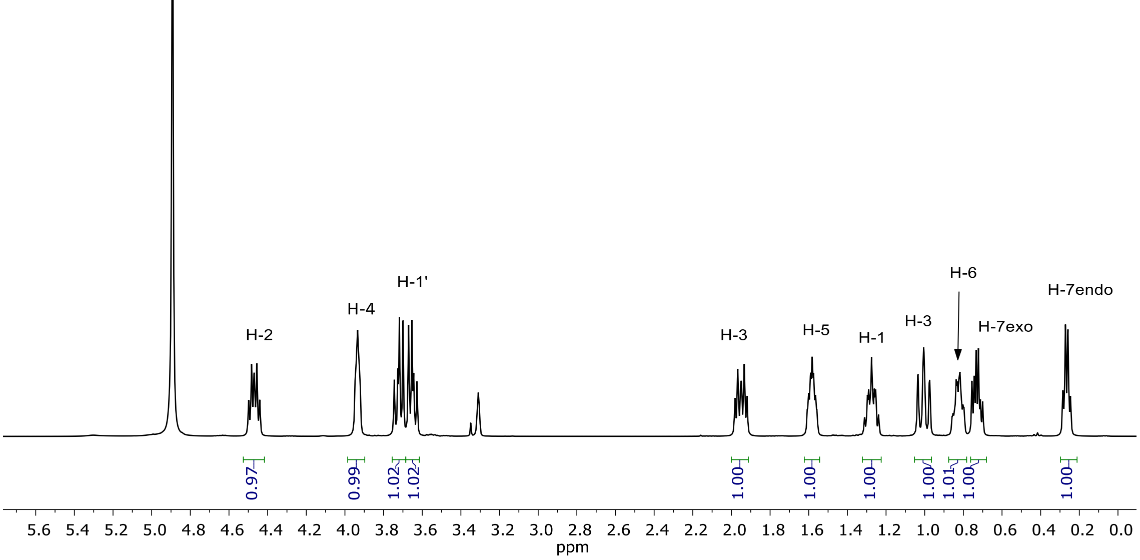

^{1}H NMR (400 MHz, MeOH-d_4)

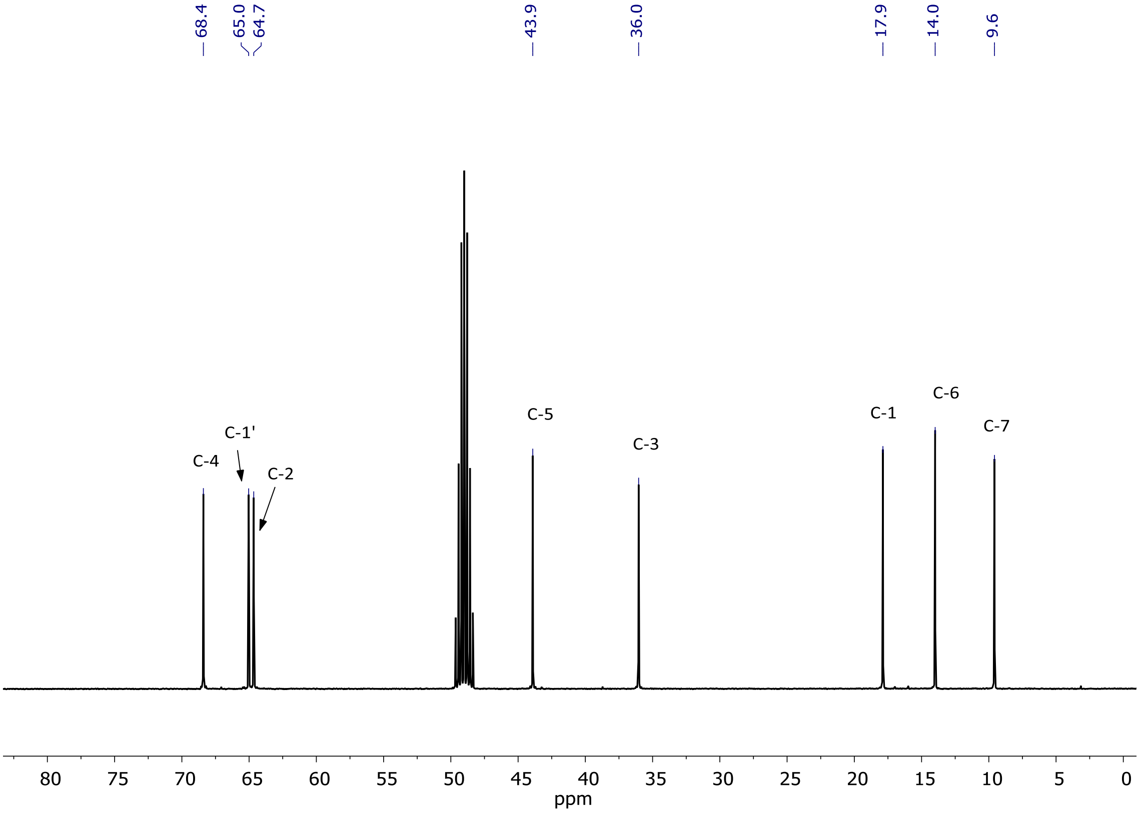

^{13}C{^{1}H} NMR (100 MHz, MeOH-d_4)

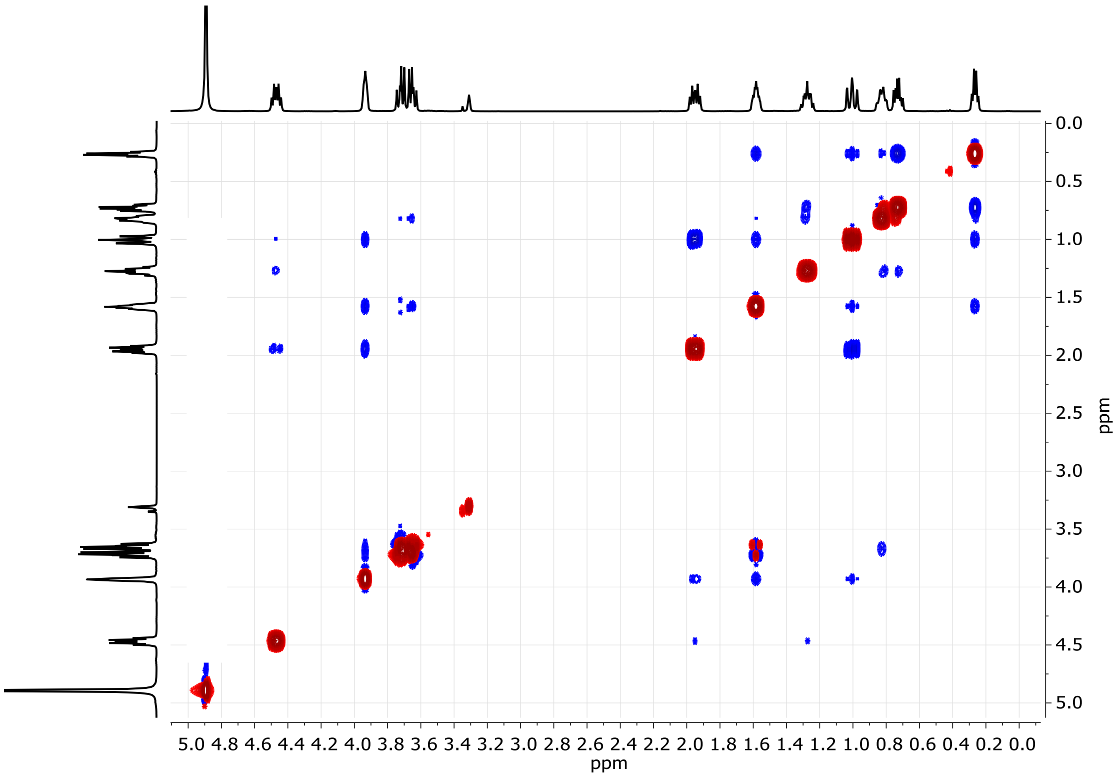

NOESY (400 MHz, MeOH-d_4)

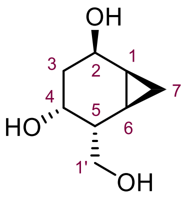

12

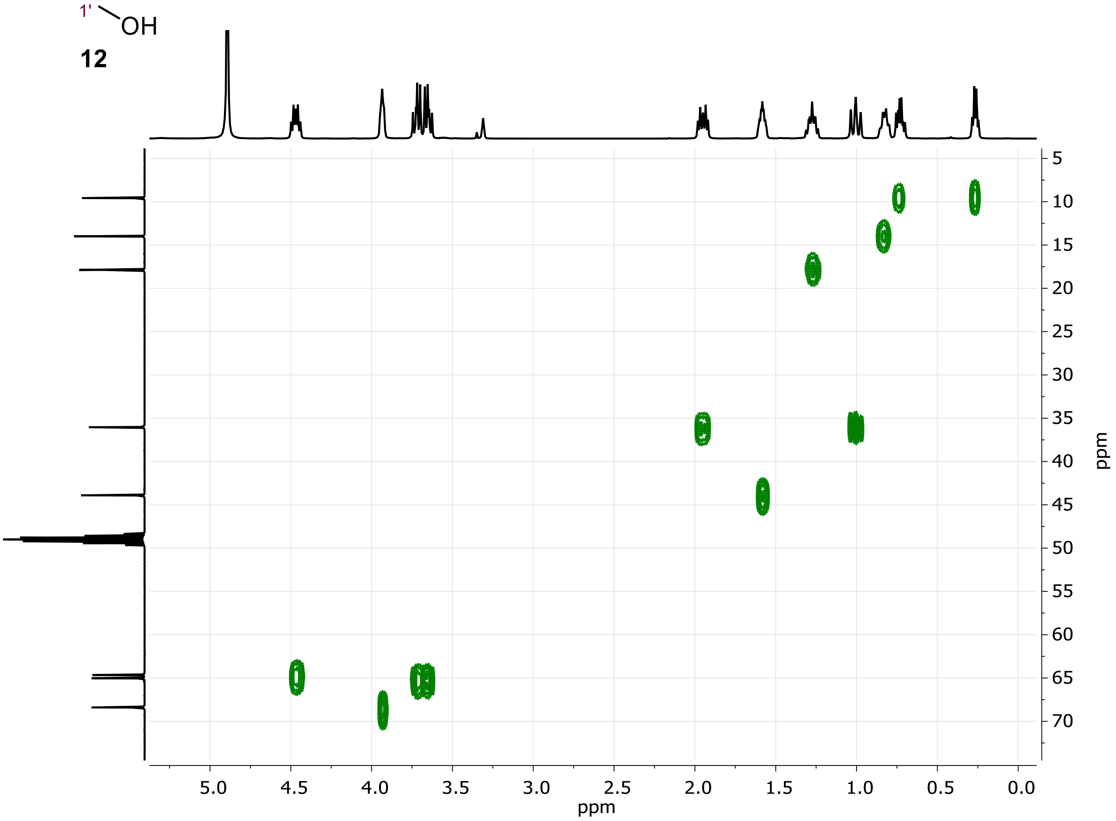

HSQC (400 MHz, MeOH-d_4)

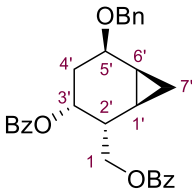

13

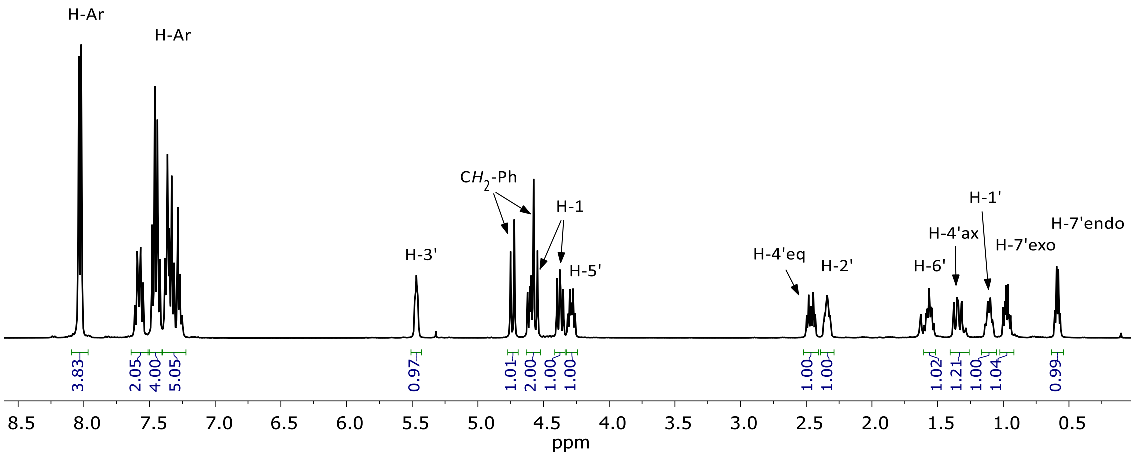

^{1}H NMR (400 MHz, $CDCl_3$)

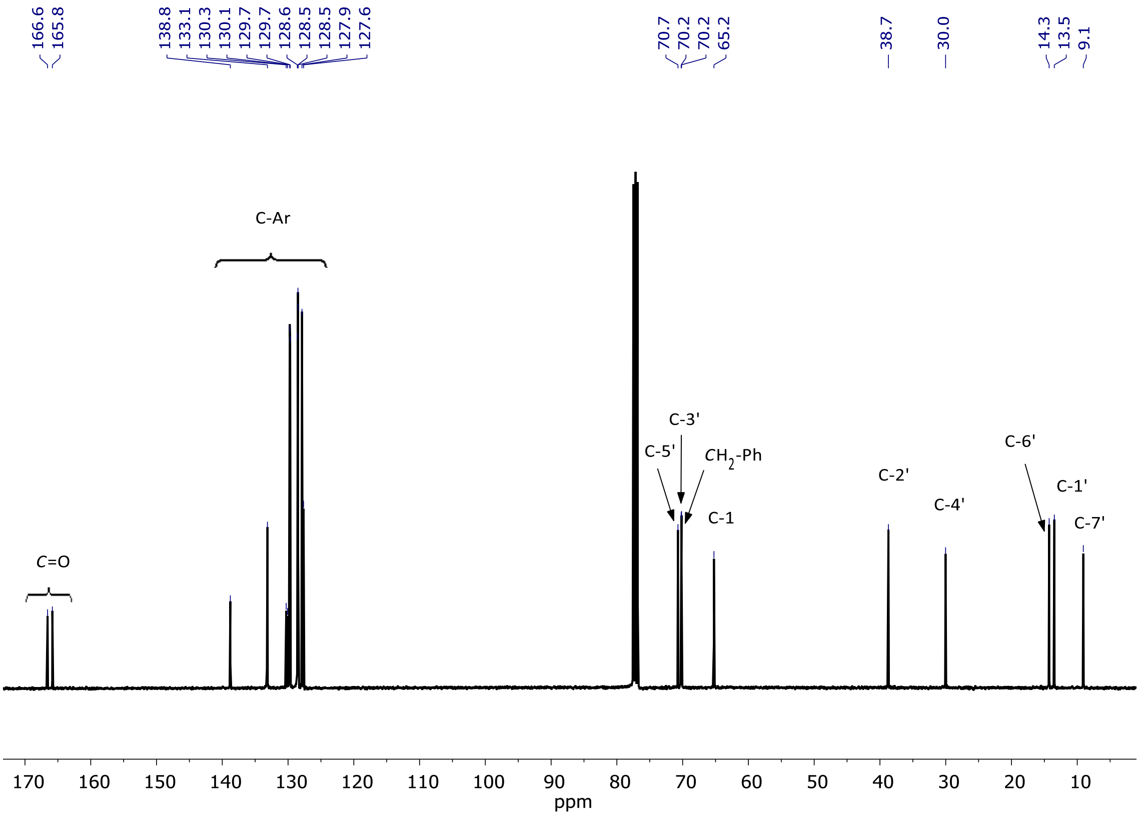

^{13}C{^{1}H} NMR (100 MHz, $CDCl_3$)

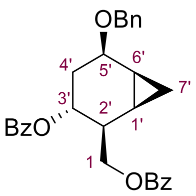

14

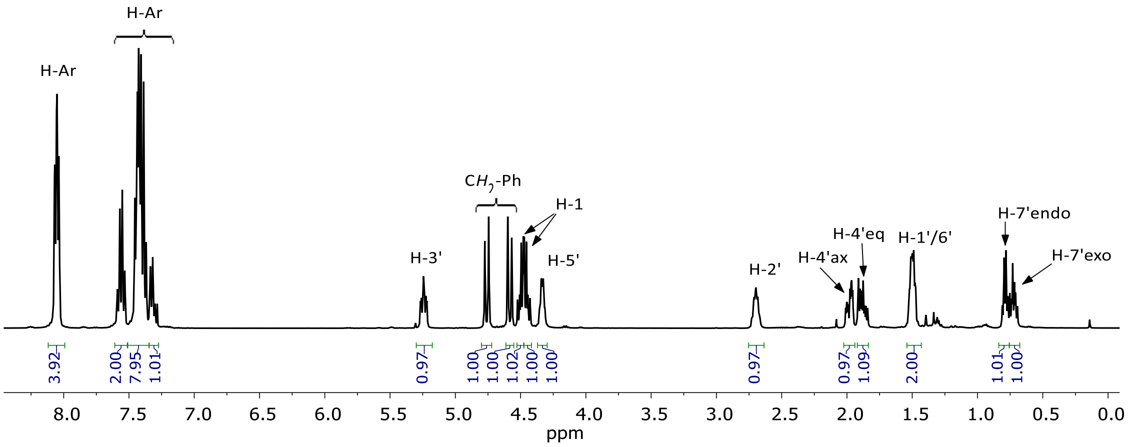

^{1}H NMR (400 MHz, $CDCl_3$)

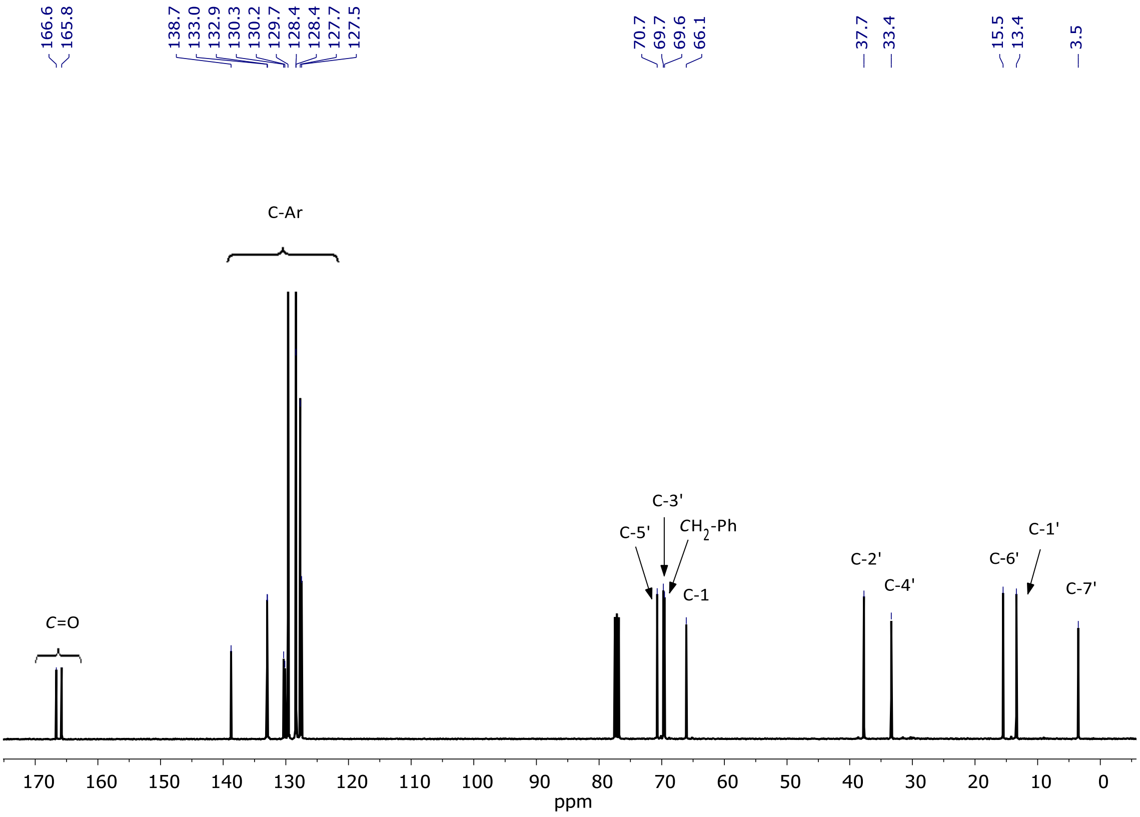

^{13}C{^{1}H} NMR (100 MHz, $CDCl_3$)

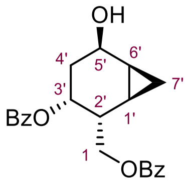

15

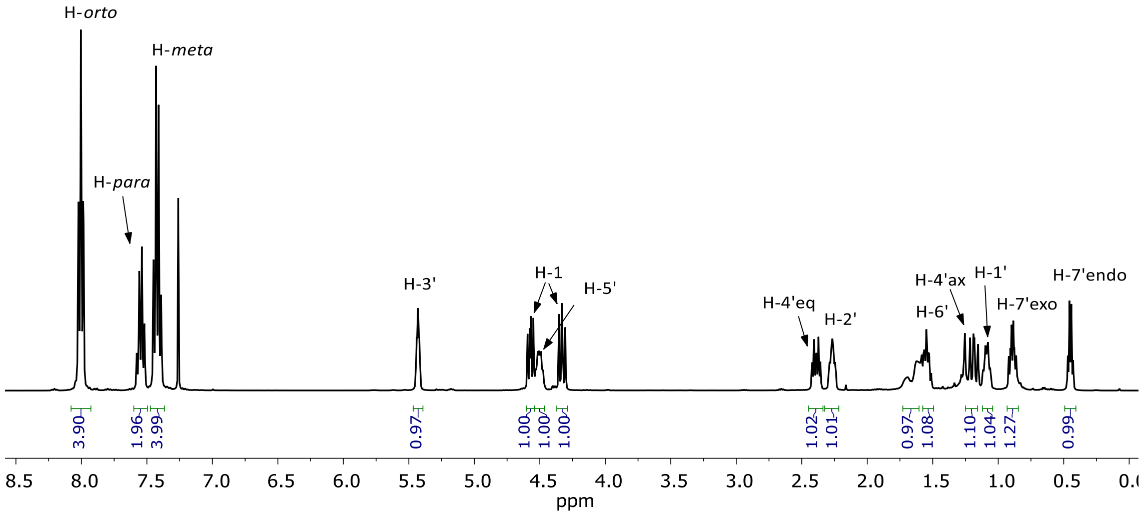

^{1}H NMR (400 MHz, $CDCl_3$)

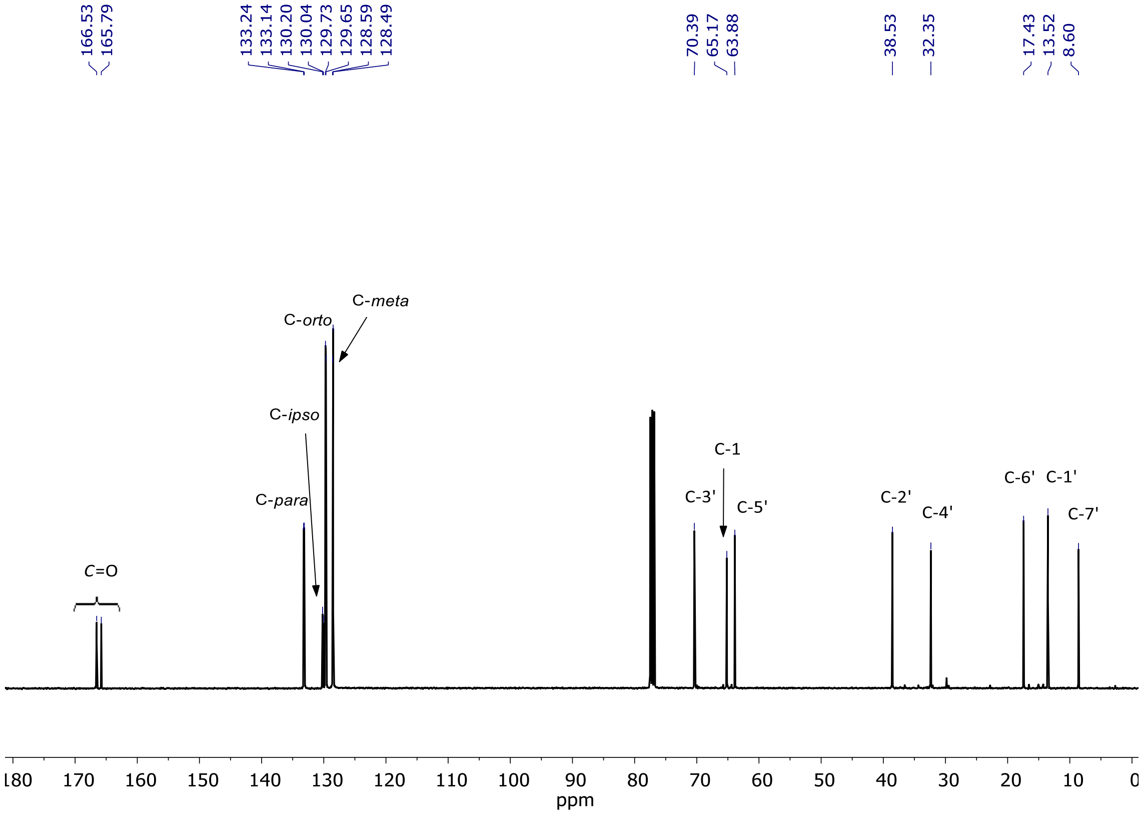

^{13}C{^{1}H} NMR (100 MHz, $CDCl_3$)

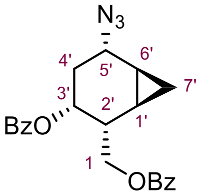

6

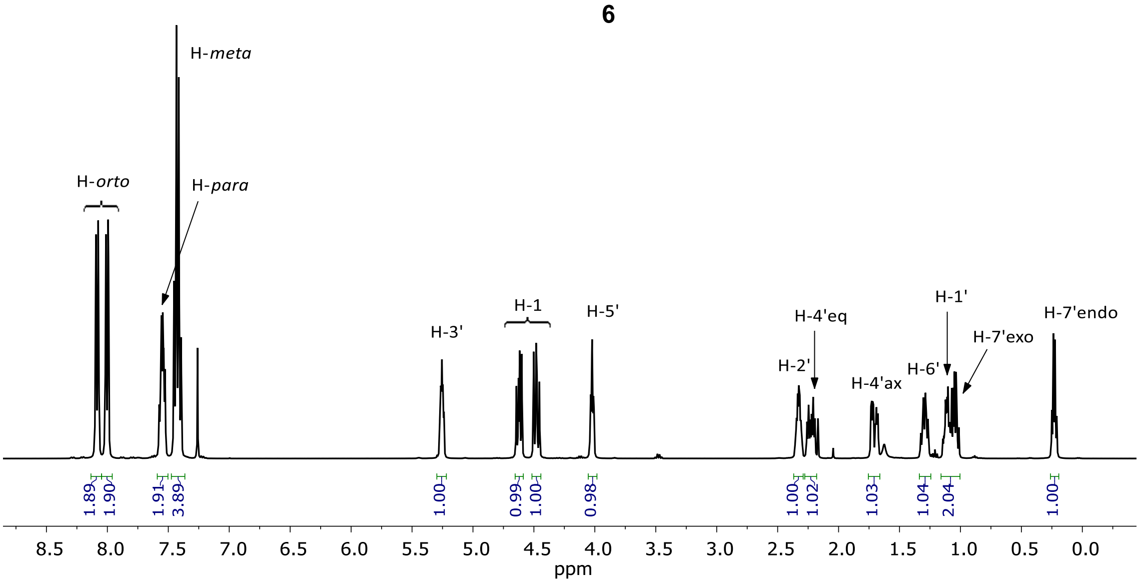

^{1}H NMR (400 MHz, $CDCl_3$)

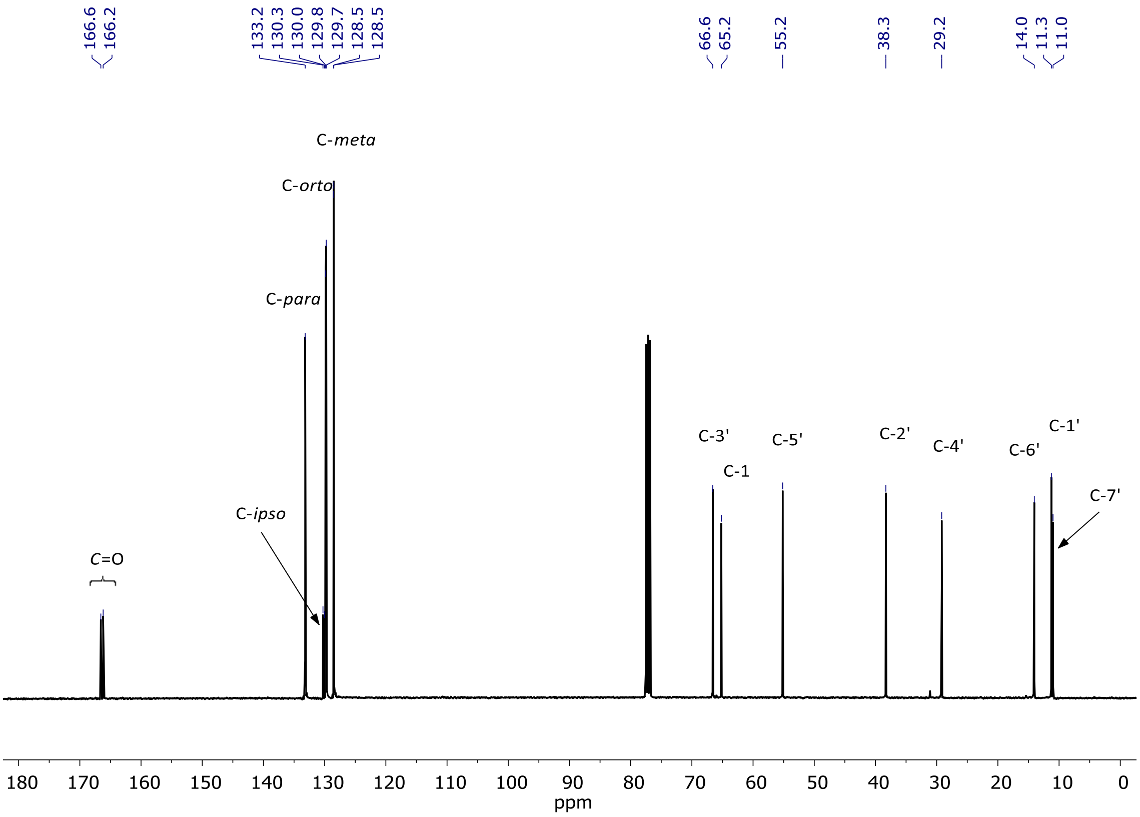

^{13}C{^{1}H} NMR (100 MHz, $CDCl_3$)

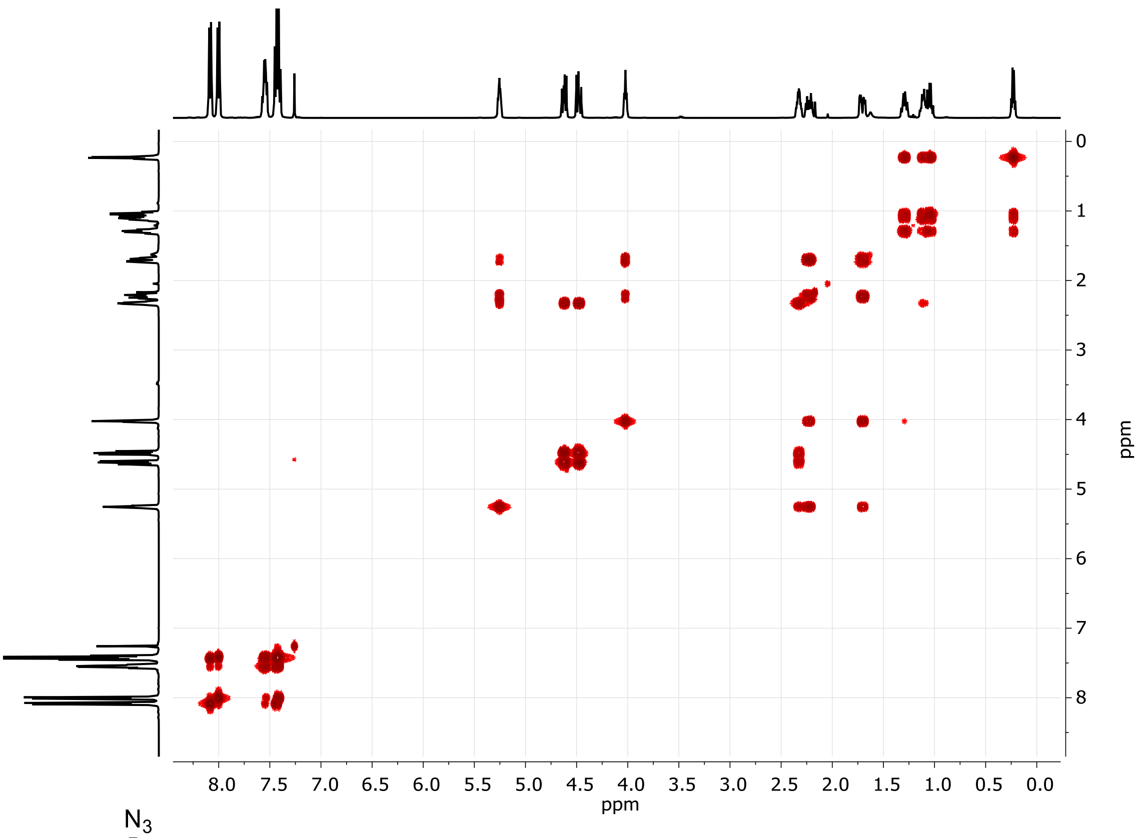

COSY (400 MHz, $CDCl_3$)

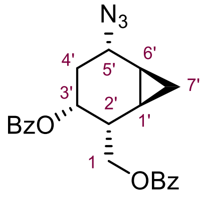

6

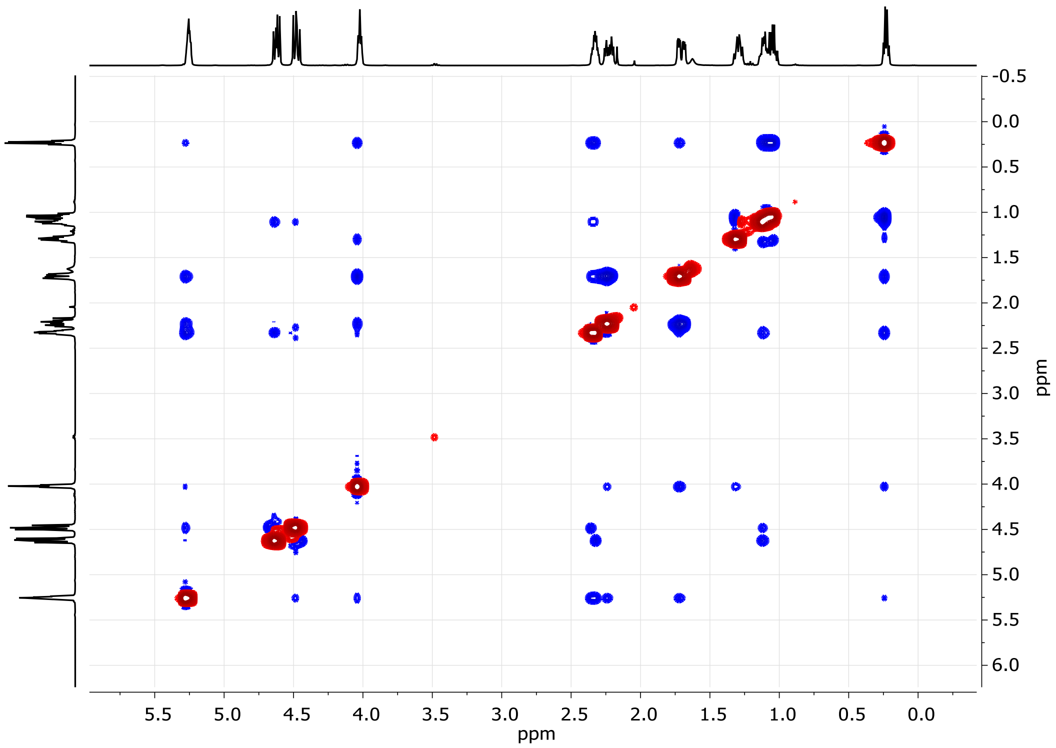

NOESY (400 MHz, $CDCl_3$)

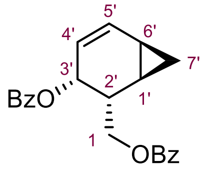

16

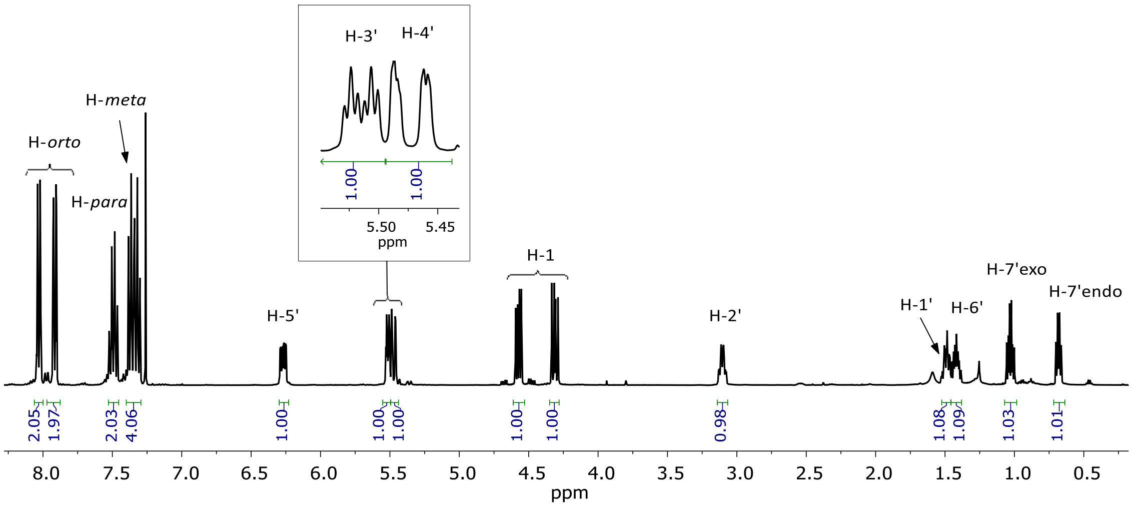

^{1}**H NMR** (400 MHz, $CDCl_3$)

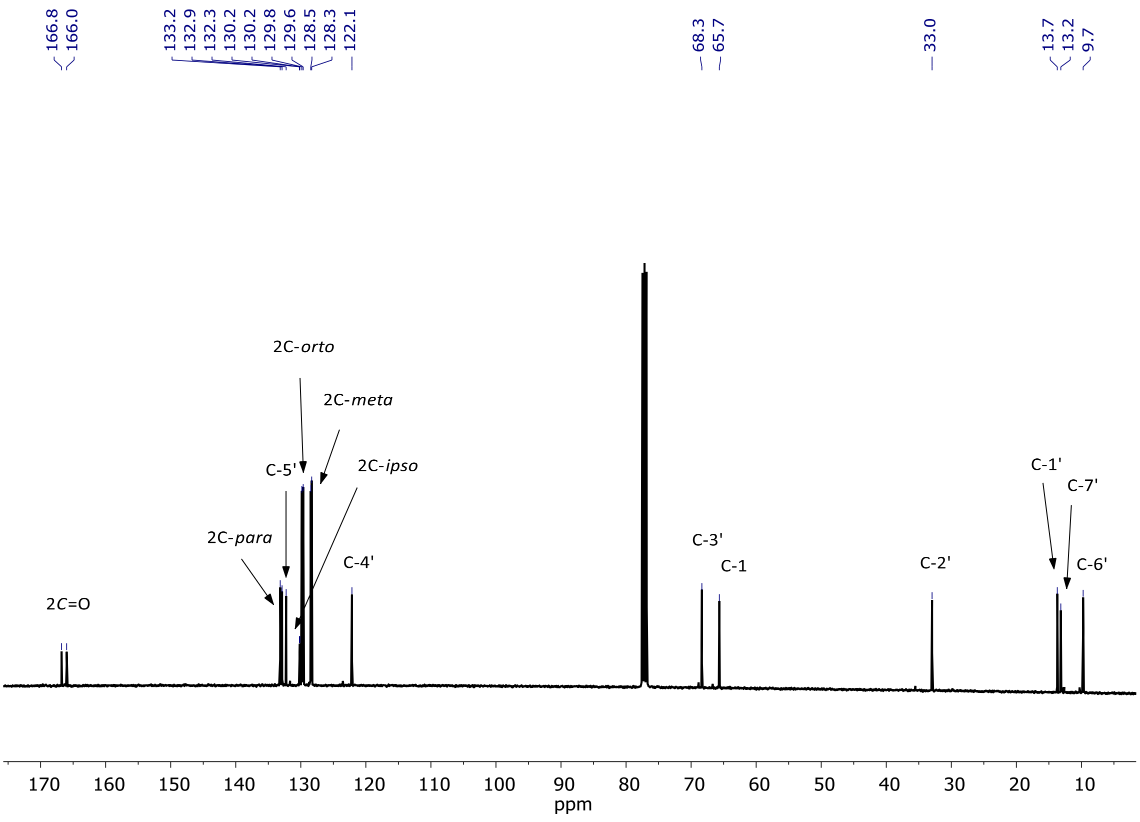

^{13}**C{^{1}H} NMR** (100 MHz, $CDCl_3$)

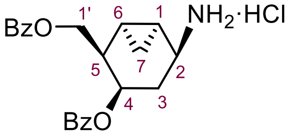

17

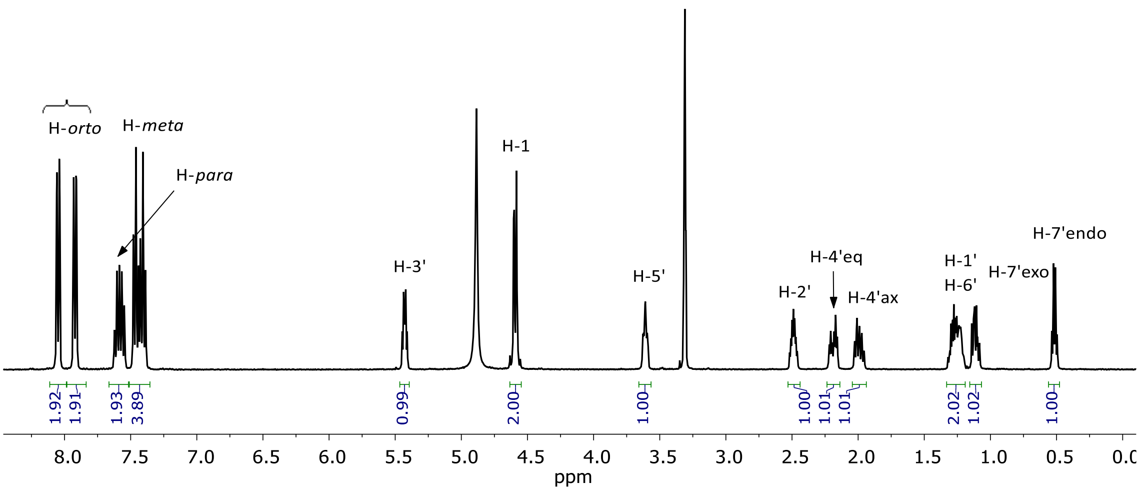

^{1}H NMR (400 MHz, MeOH-*d*$_4$)

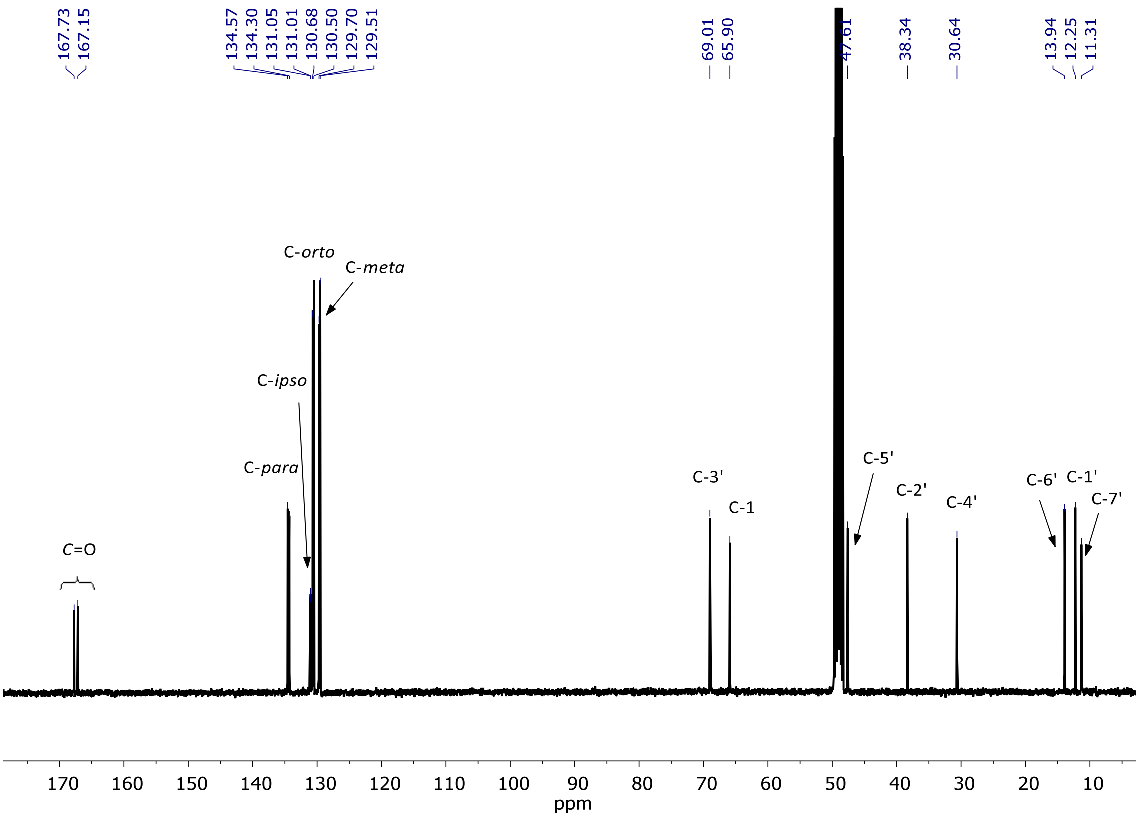

^{13}C{^{1}H} NMR (100 MHz, MeOH-*d*$_4$)

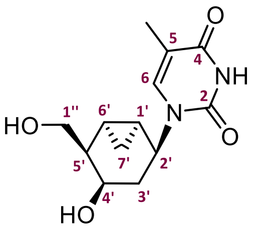

1a

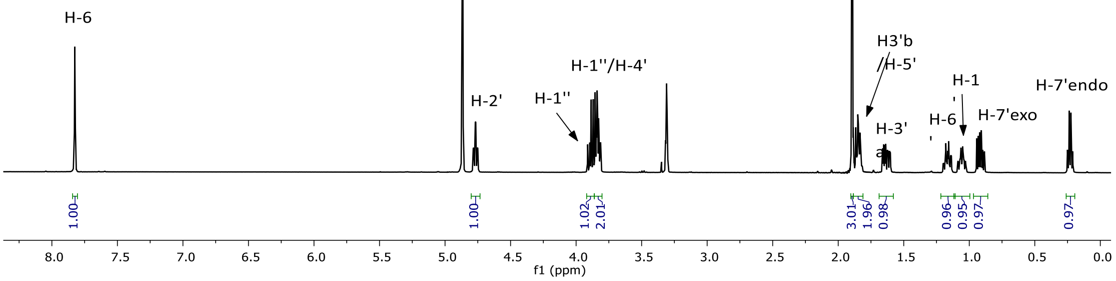

¹H NMR (400 MHz, MeOH-d_4)

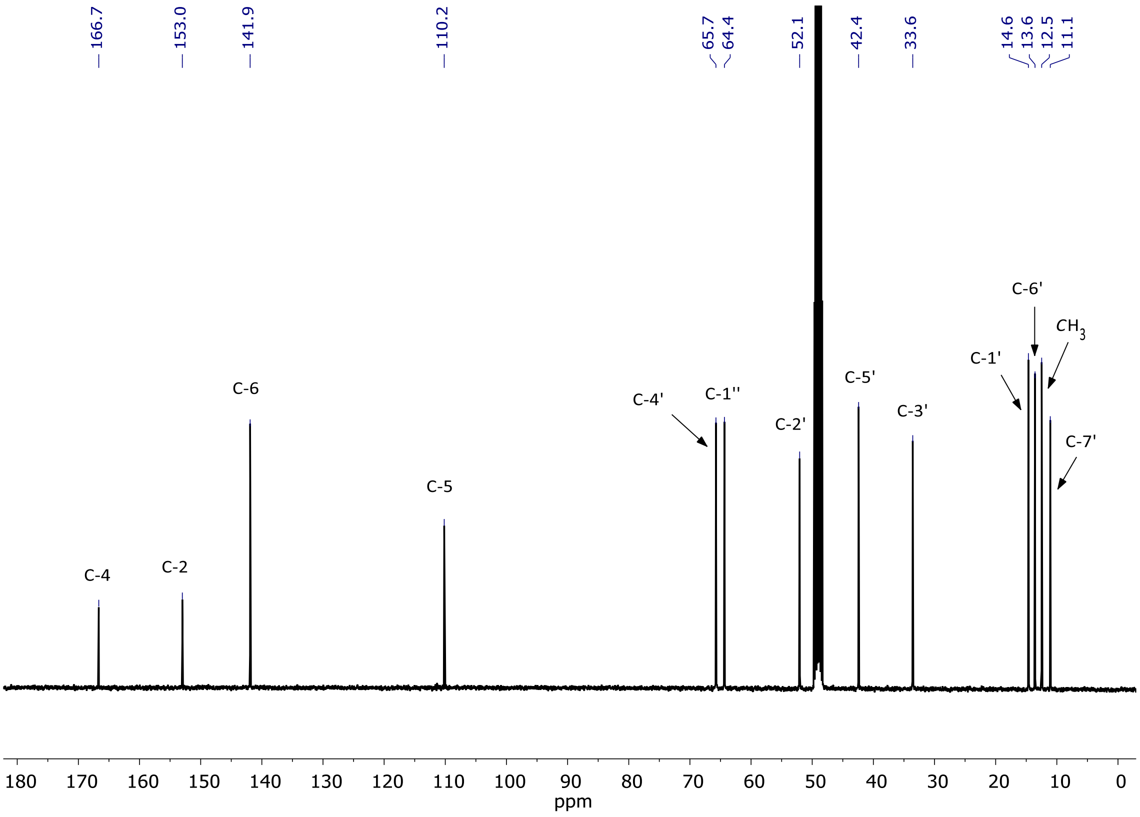

^{13}C{^{1}H} NMR (100 MHz MeOH-d_4)

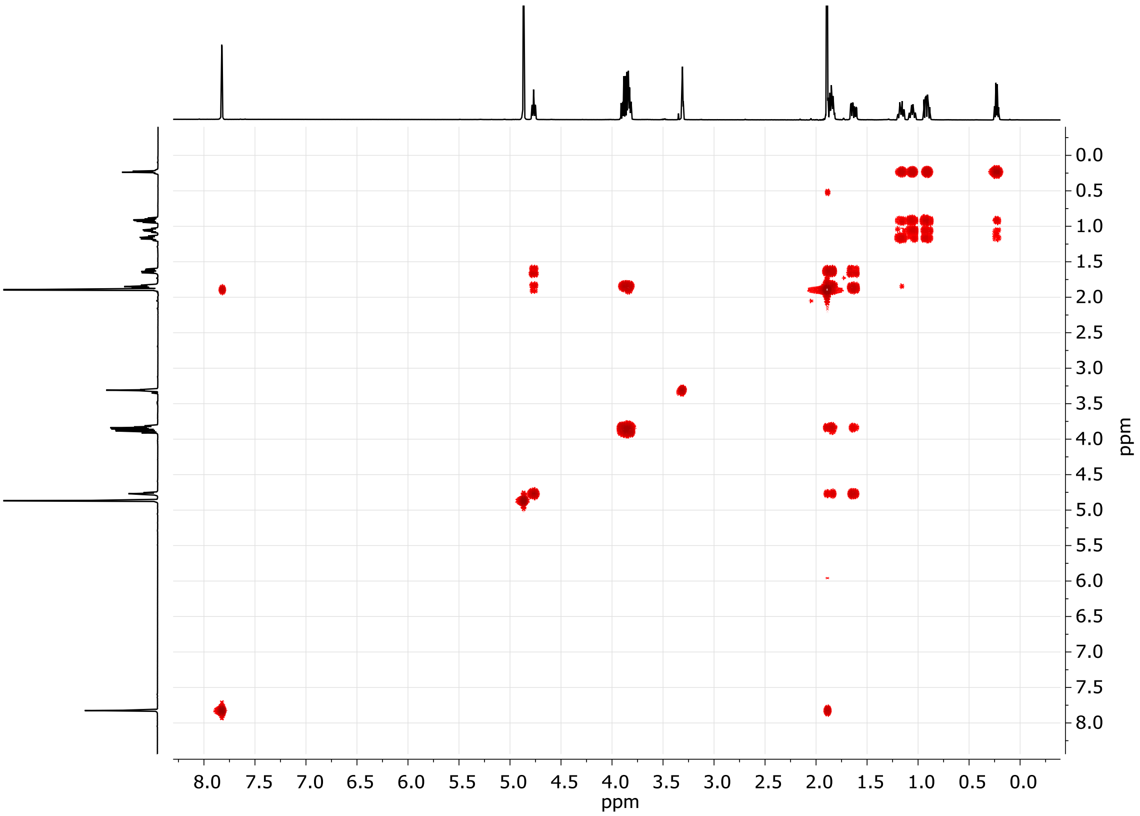

COSY (400 MHz MeOH-d_4)

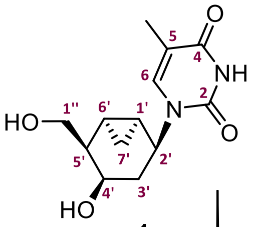

1a

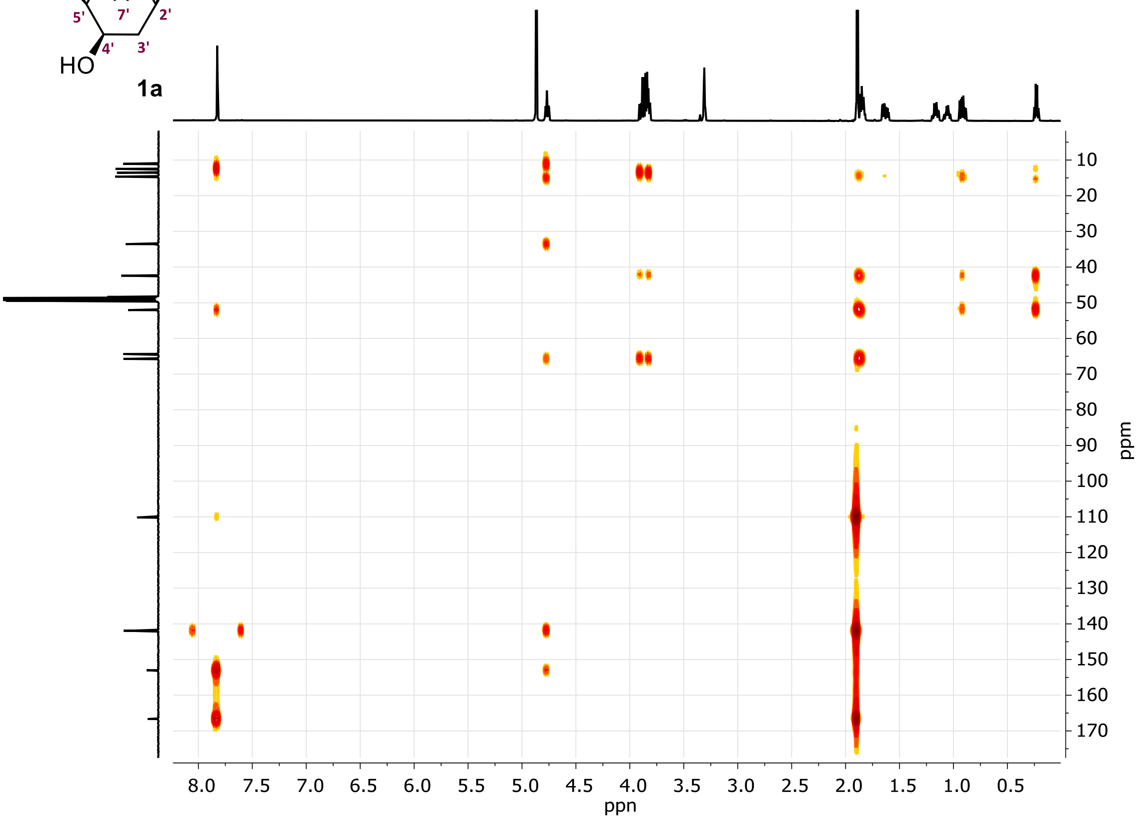

HMBC (400 MHz, MeOH-d_4)

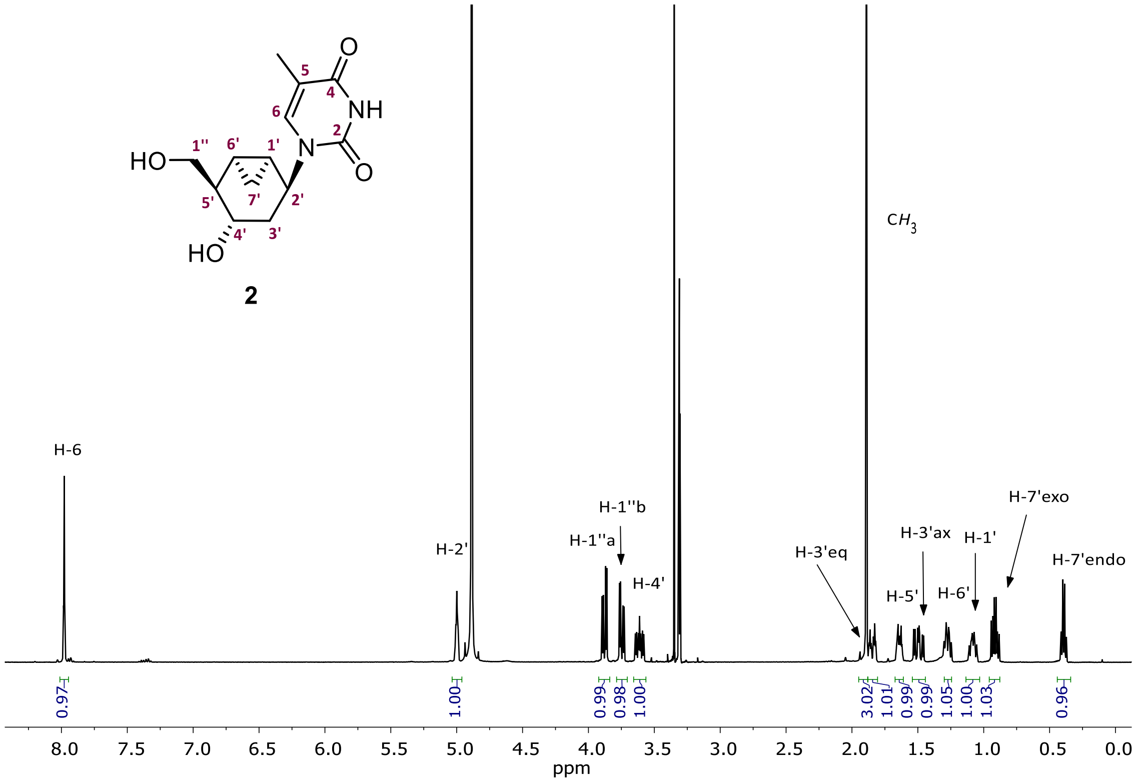

¹H NMR (400 MHz, MeOH-d_4)

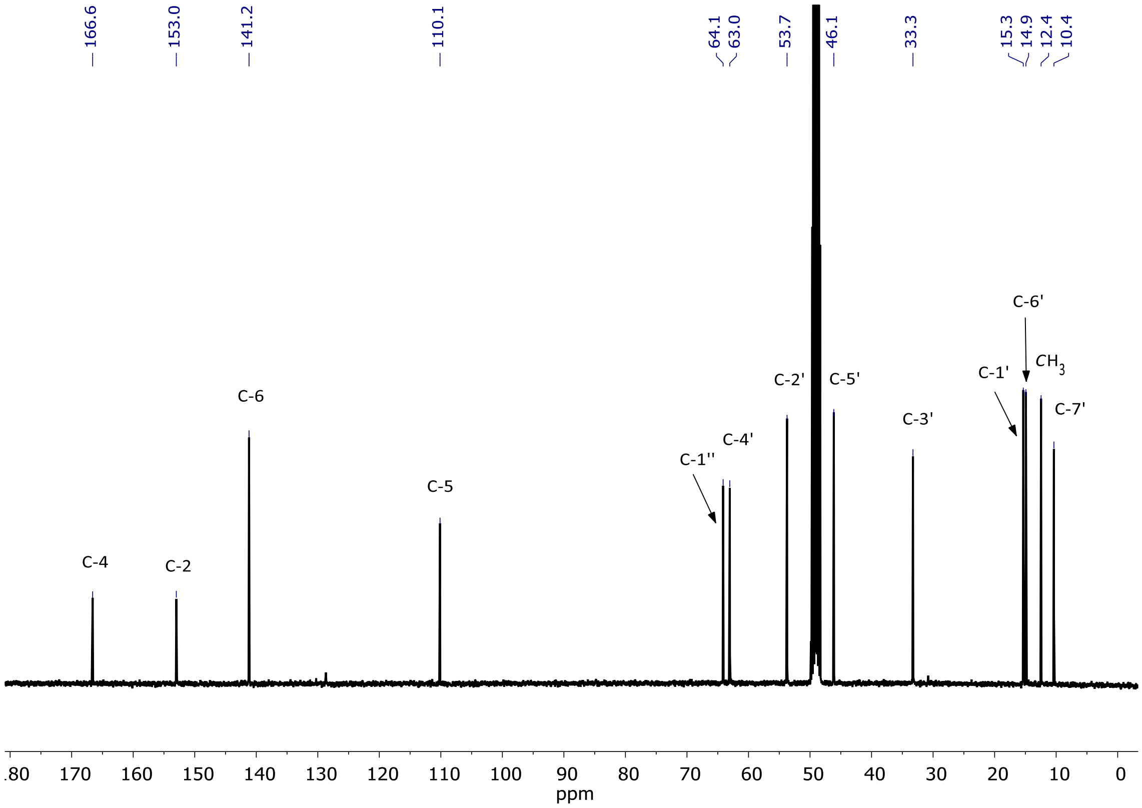

¹³C{¹H} NMR (100 MHz, MeOH-d_4)

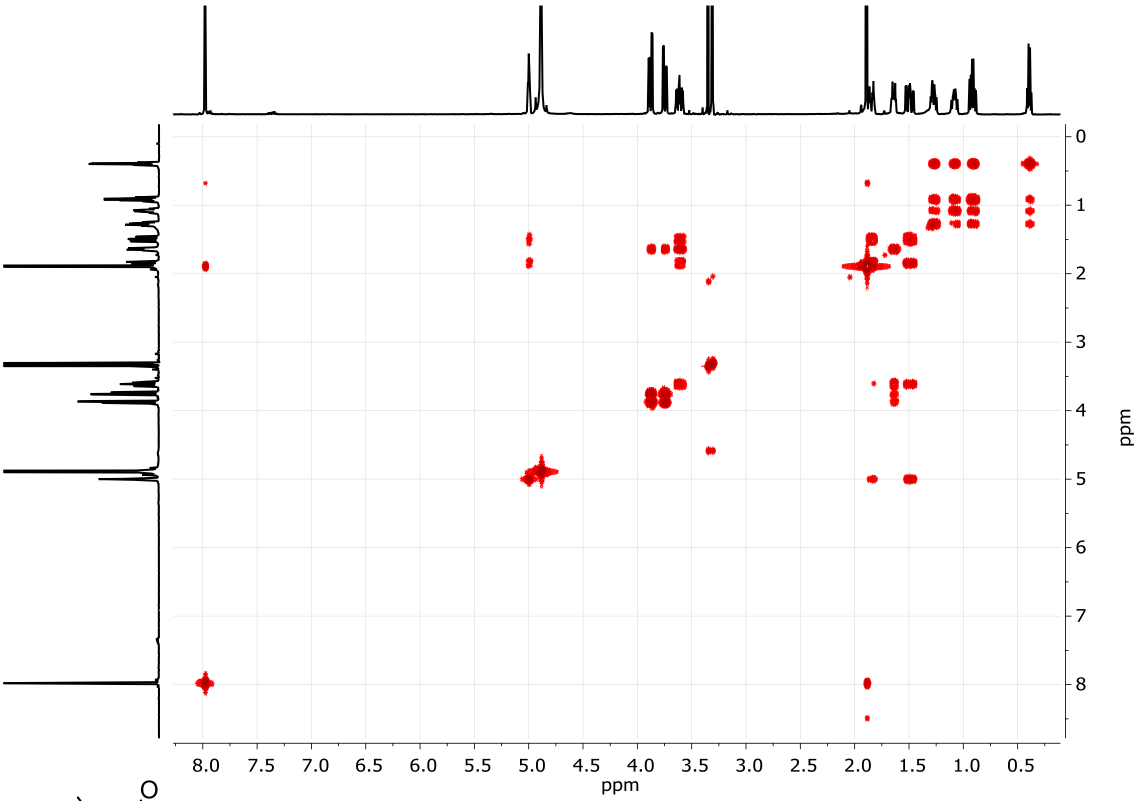

COSY (400 MHz, MeOH-d_4)

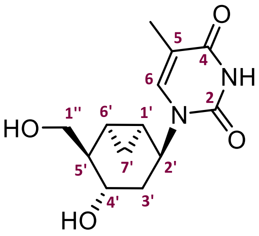

2

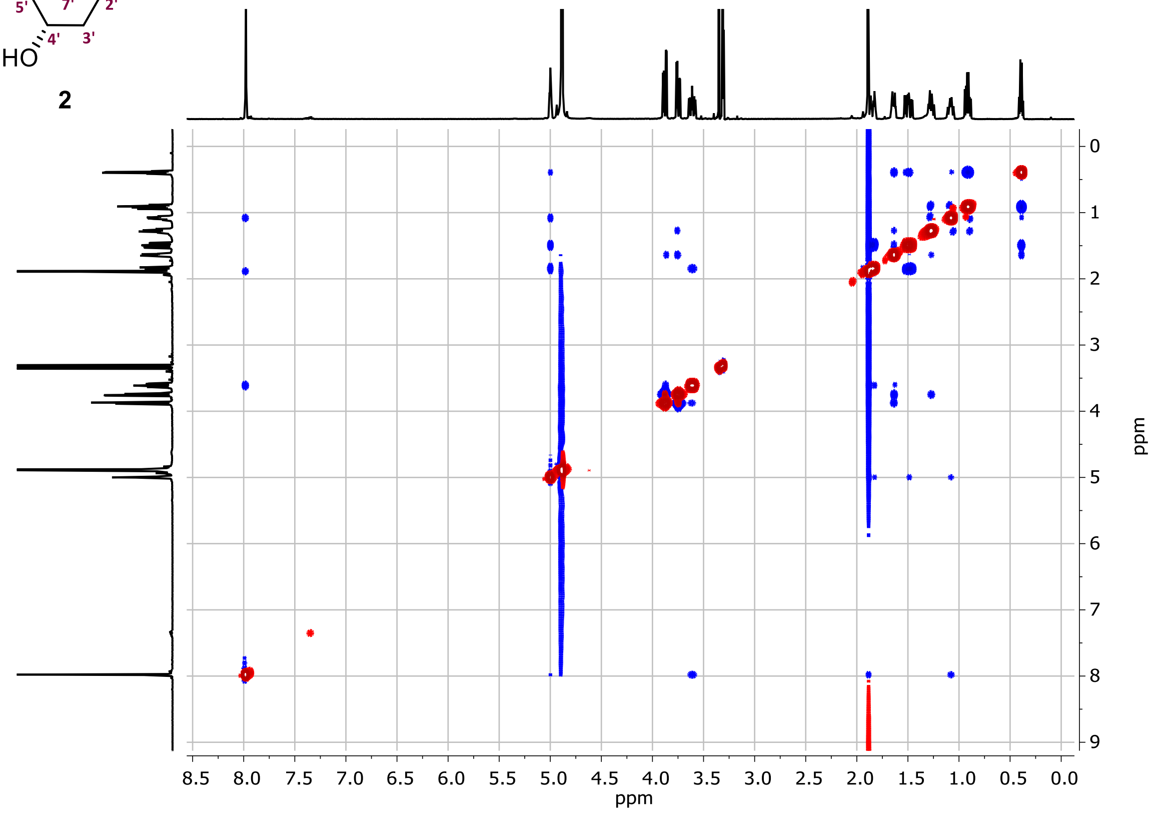

NOESY (400 MHz, MeOH-d_4)

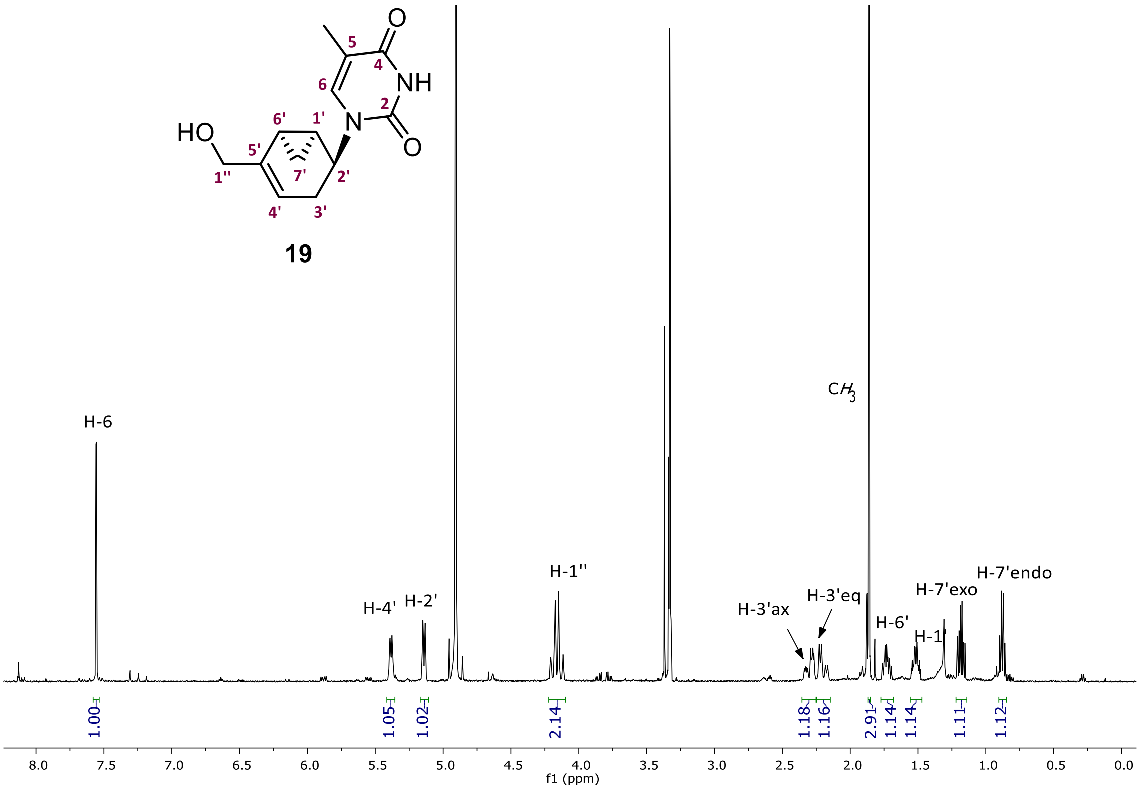

^{1}H NMR (400 MHz, MeOH-d_4)

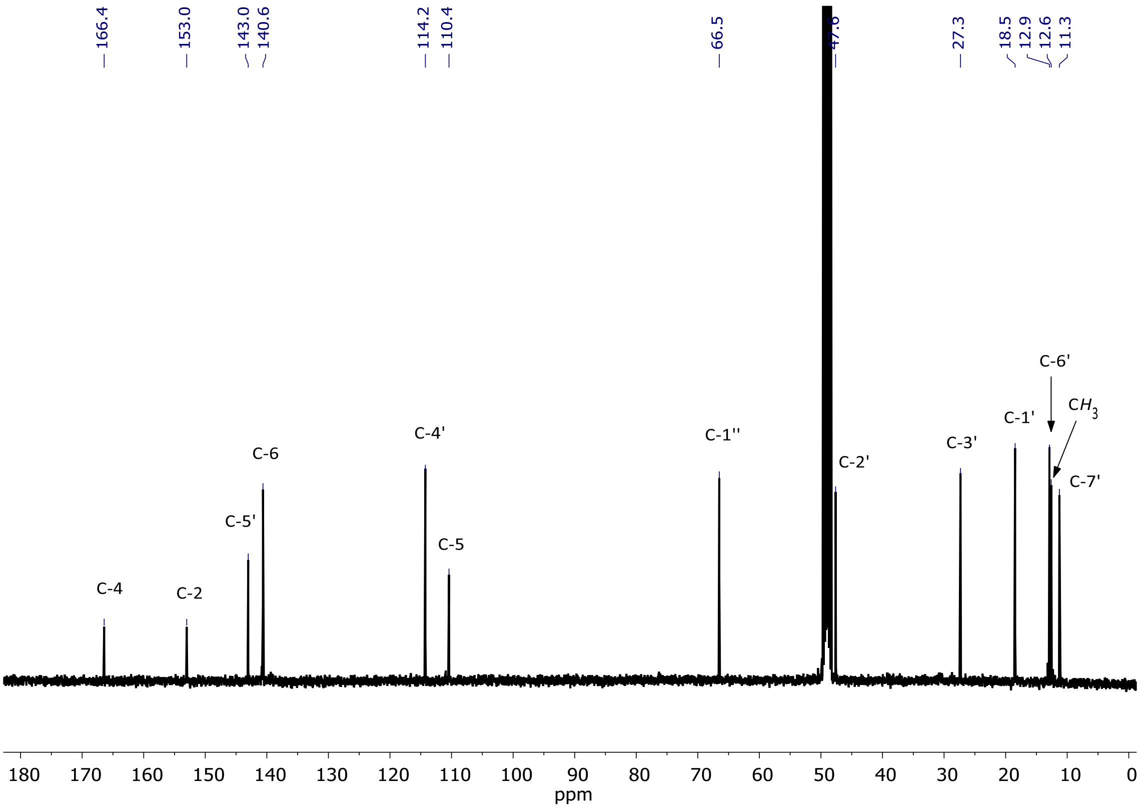

^{13}C{^{1}H} NMR (101 MHz, MeOH-d_4)

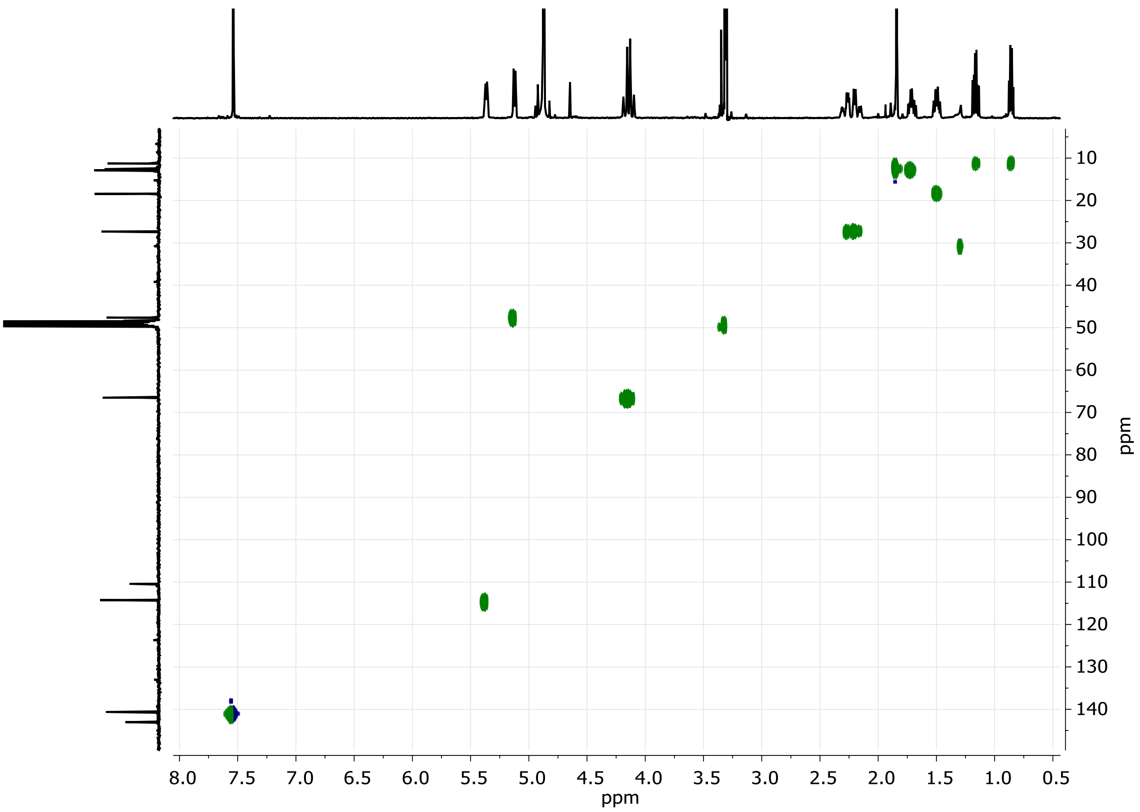

HSQC (400 MHz, MeOH-d_4)

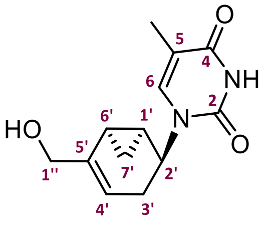

19

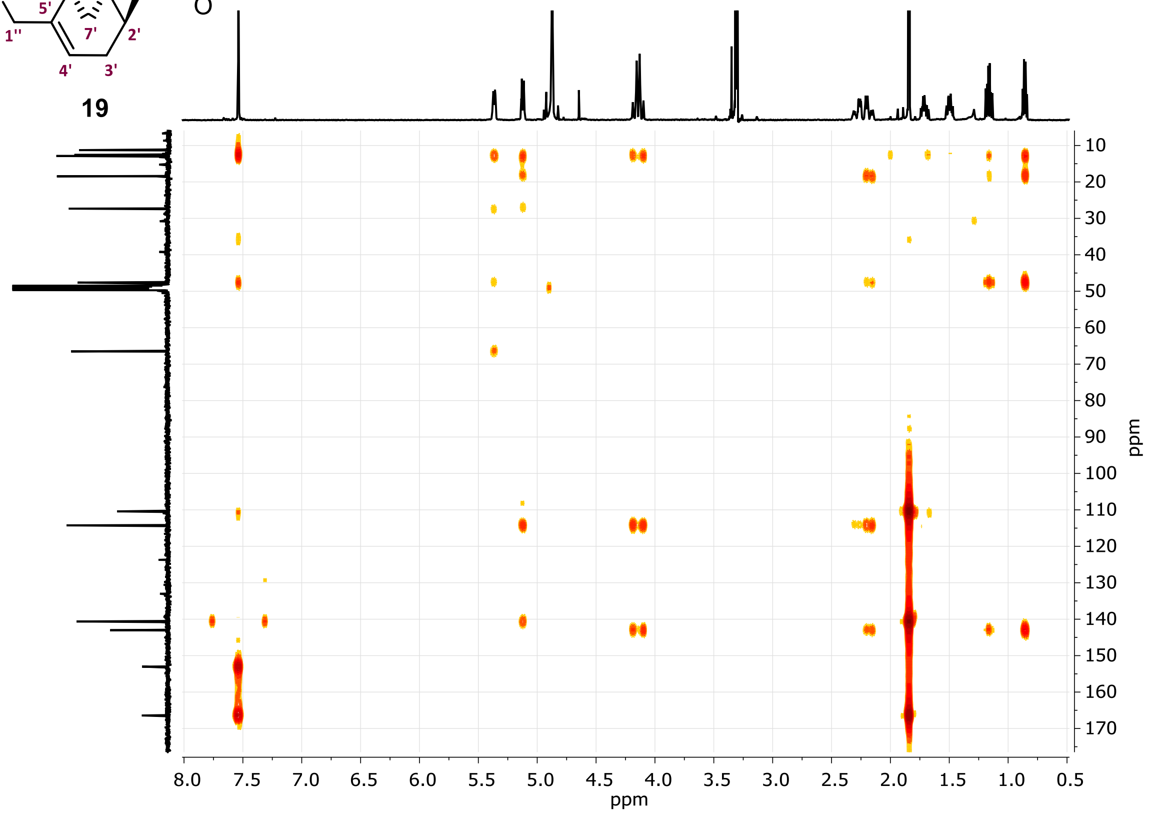

HMBC (400 MHz, MeOH-d_4)

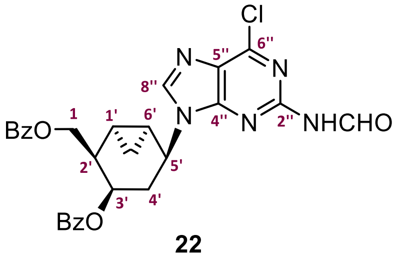

22

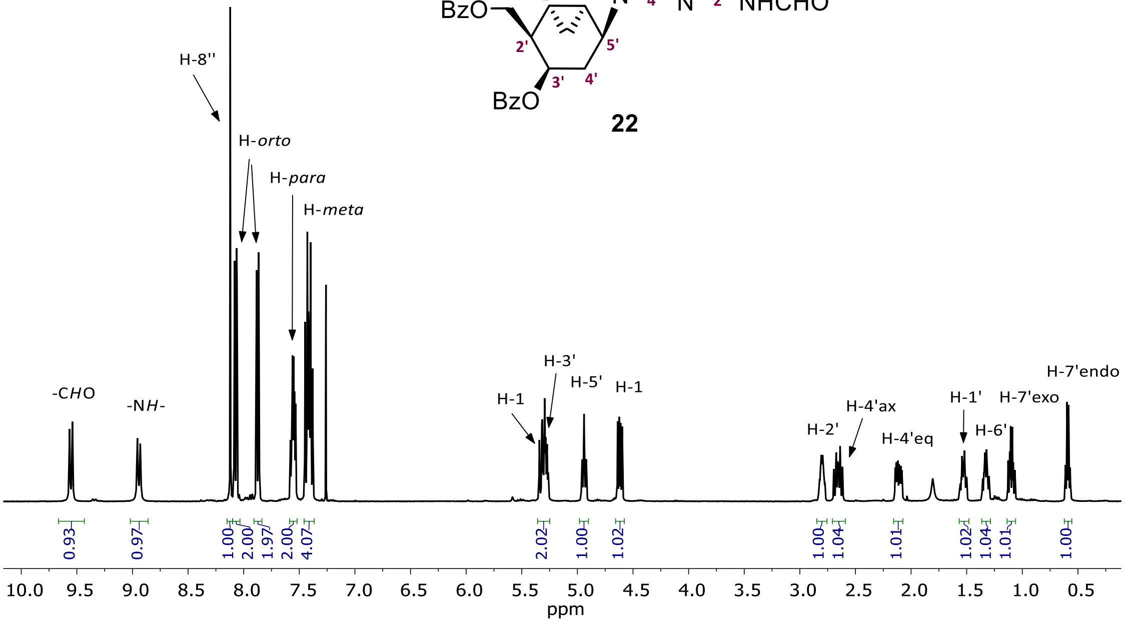

^{1}H NMR (400 MHz, $CDCl_3$)

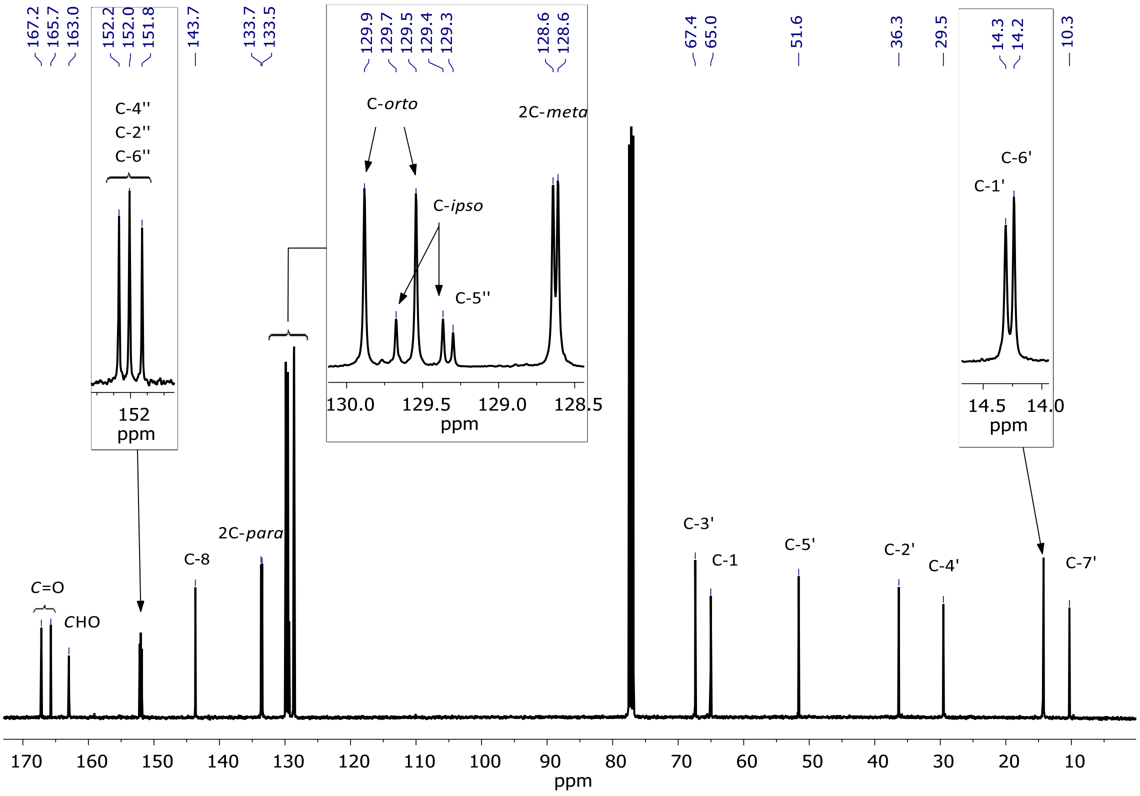

^{13}C{^{1}H} NMR (100 MHz, $CDCl_3$)

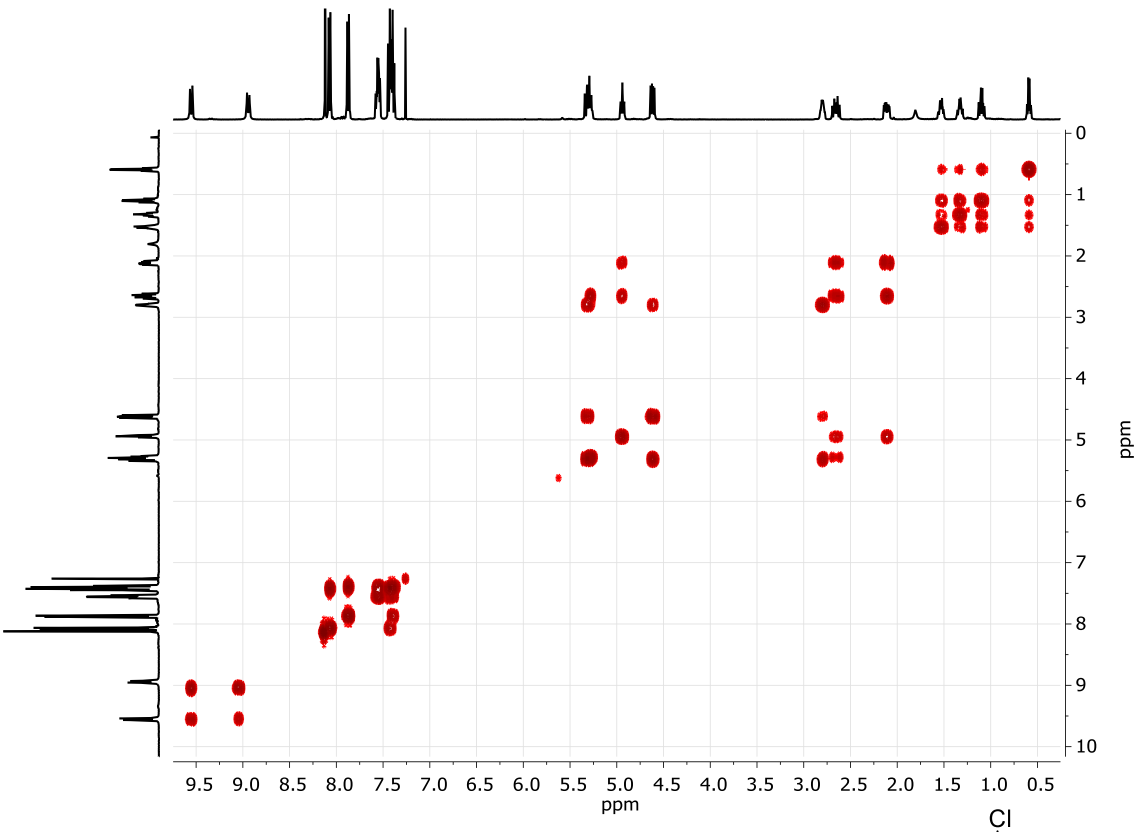

COSY (400 MHz, $CDCl_3$)

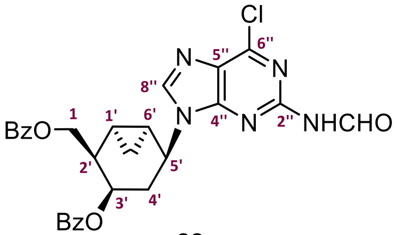

22

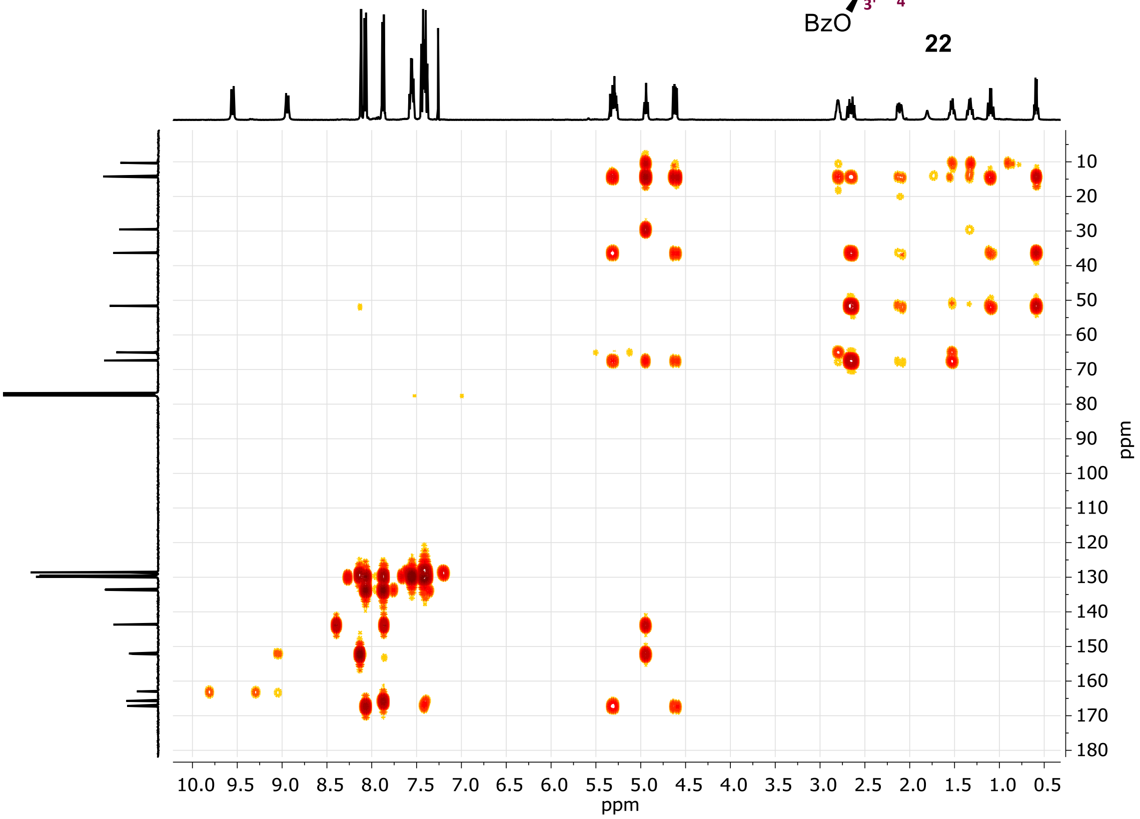

HMBC (400 MHz, $CDCl_3$)

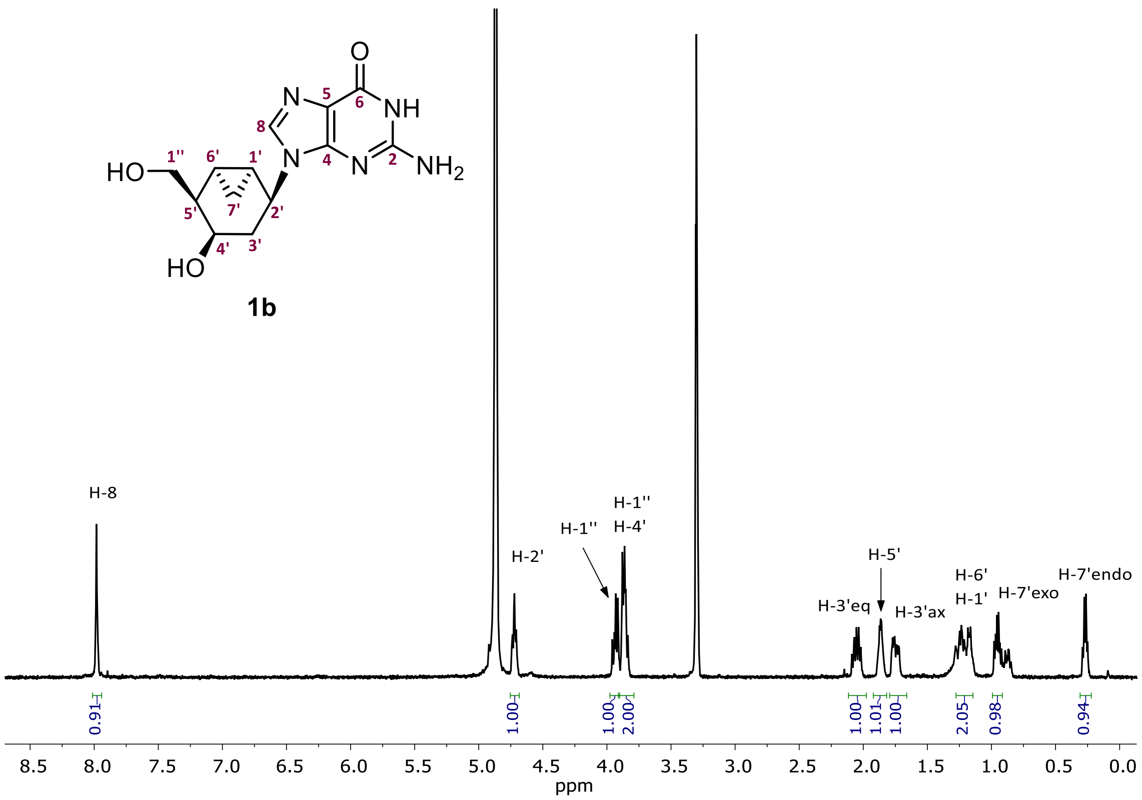

^{1}H NMR (400 MHz, MeOH-d_4)

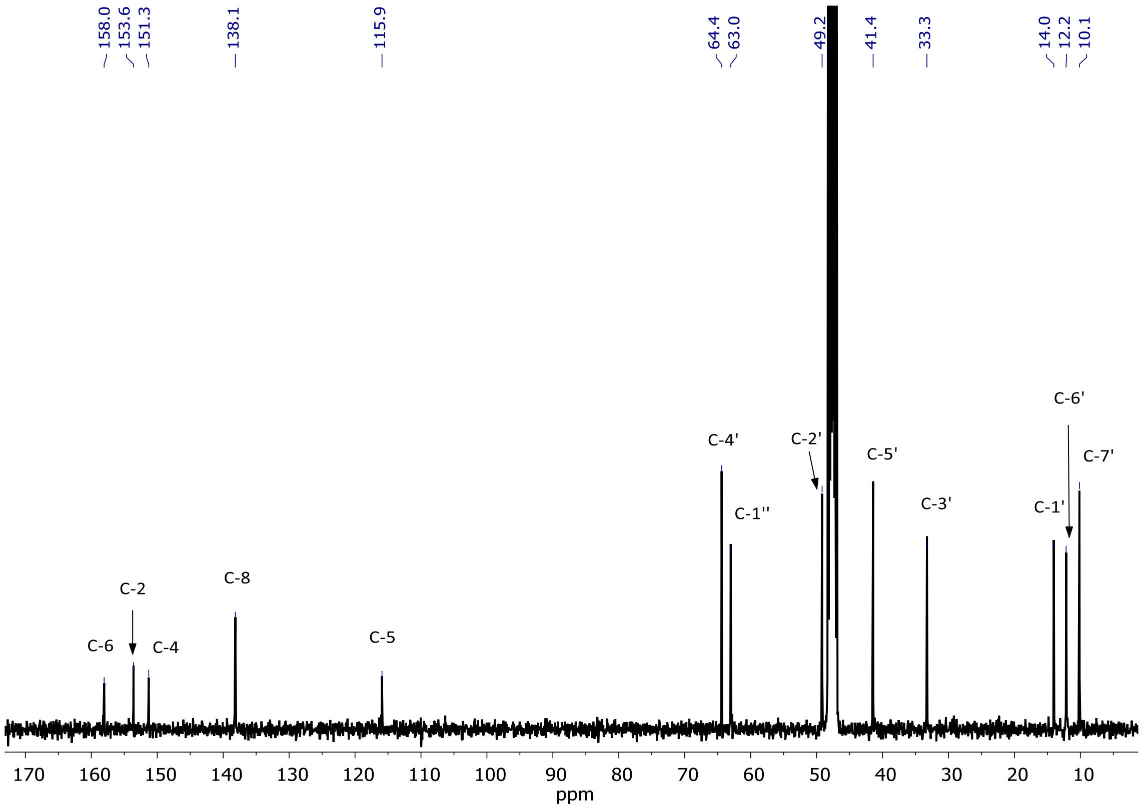

^{13}C{^{1}H} NMR (100 MHz, MeOH-d_4)

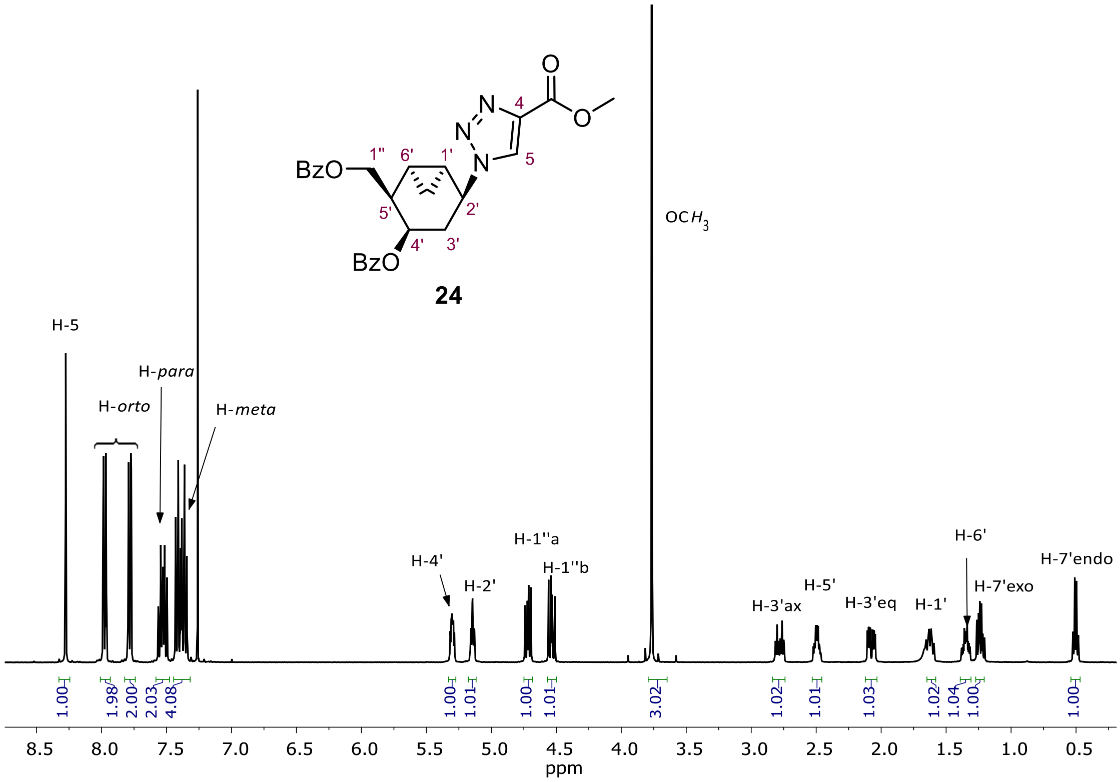

^{1}**H NMR** (400 MHz, $CDCl_3$)

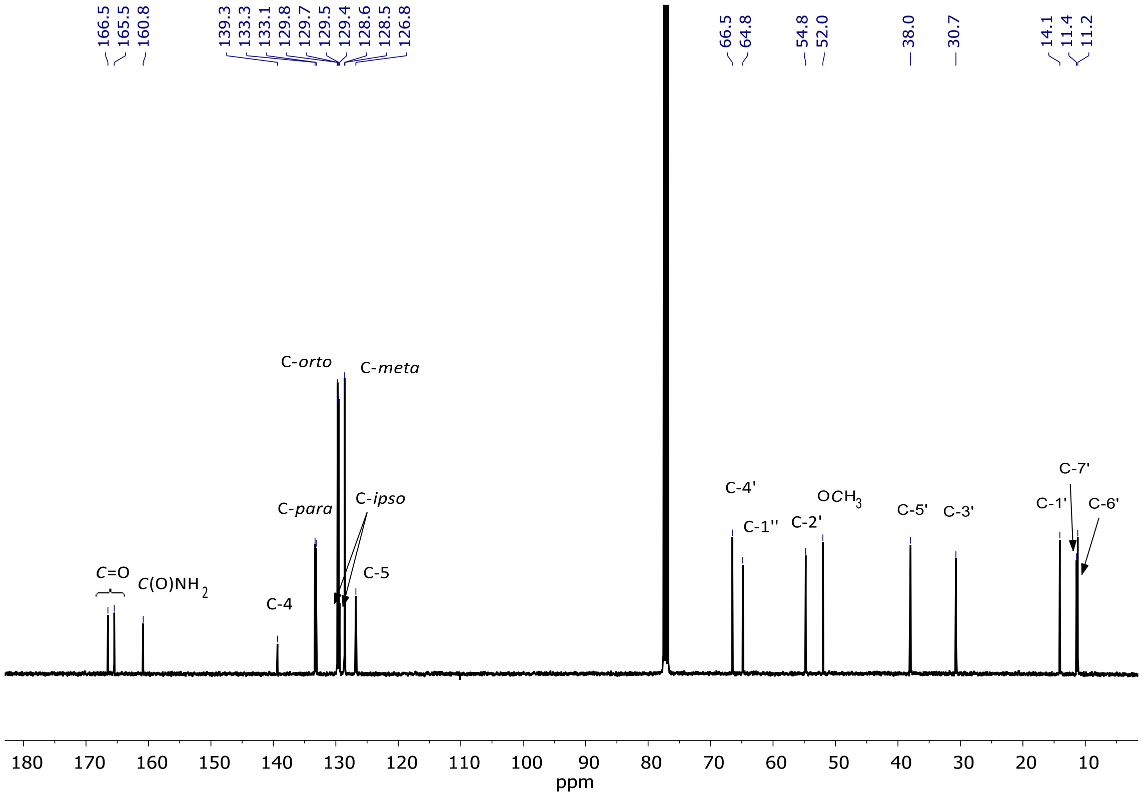

^{13}**C{^{1}H} NMR** (100 MHz, $CDCl_3$)

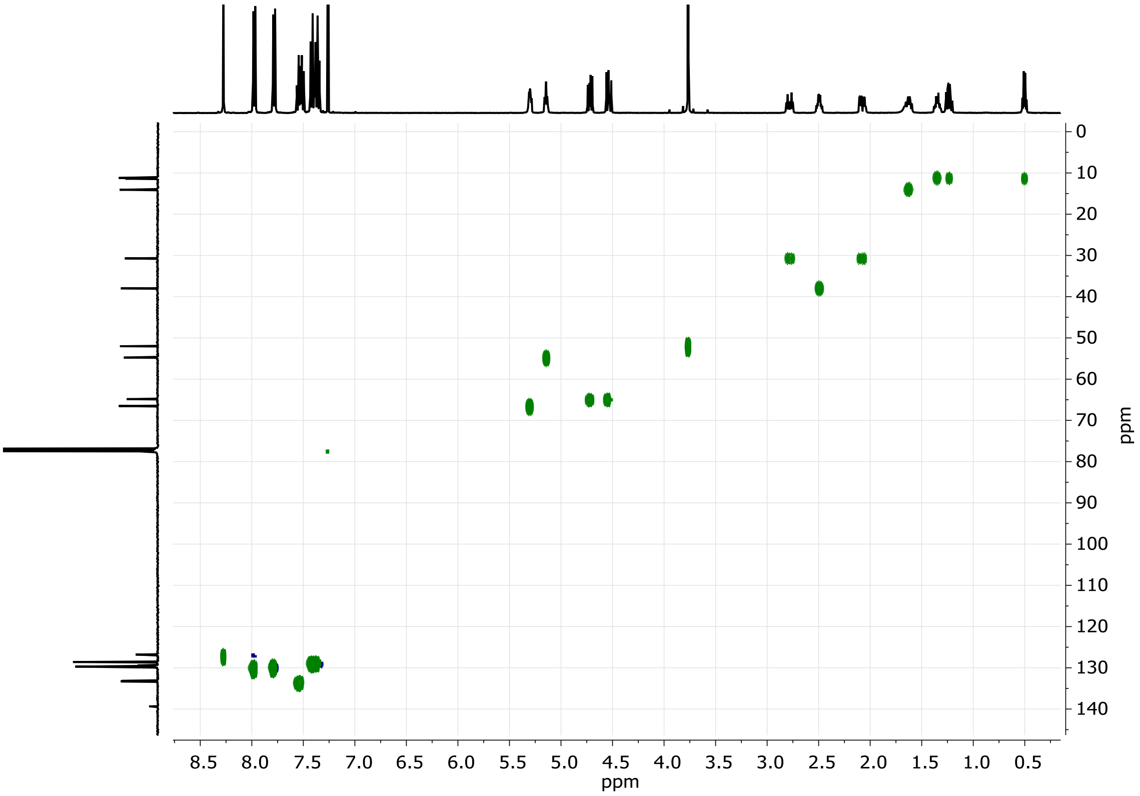

HSQC (400 MHz, $CDCl_3$)

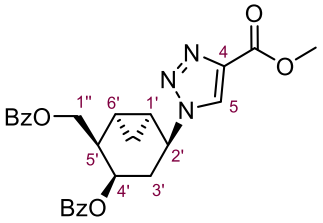

24

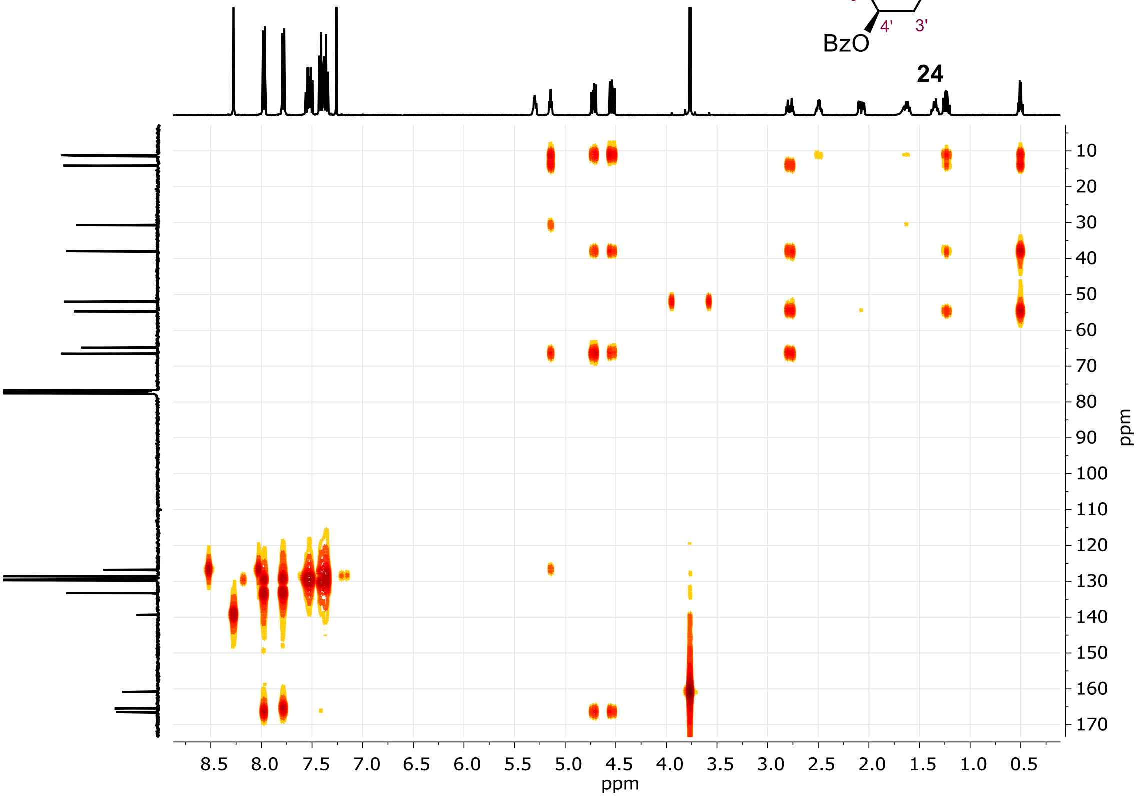

HMBC (400 MHz, $CDCl_3$)

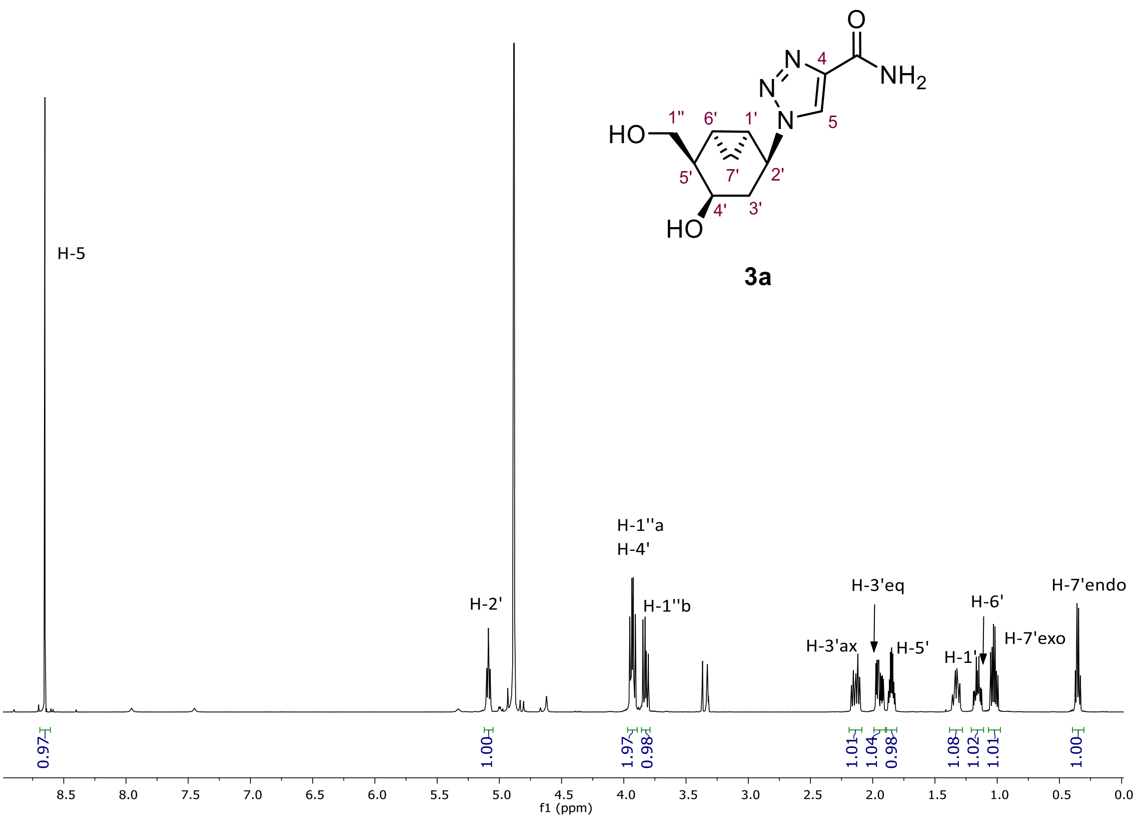

^{1}**H NMR** (400 MHz, MeOH-d_4)

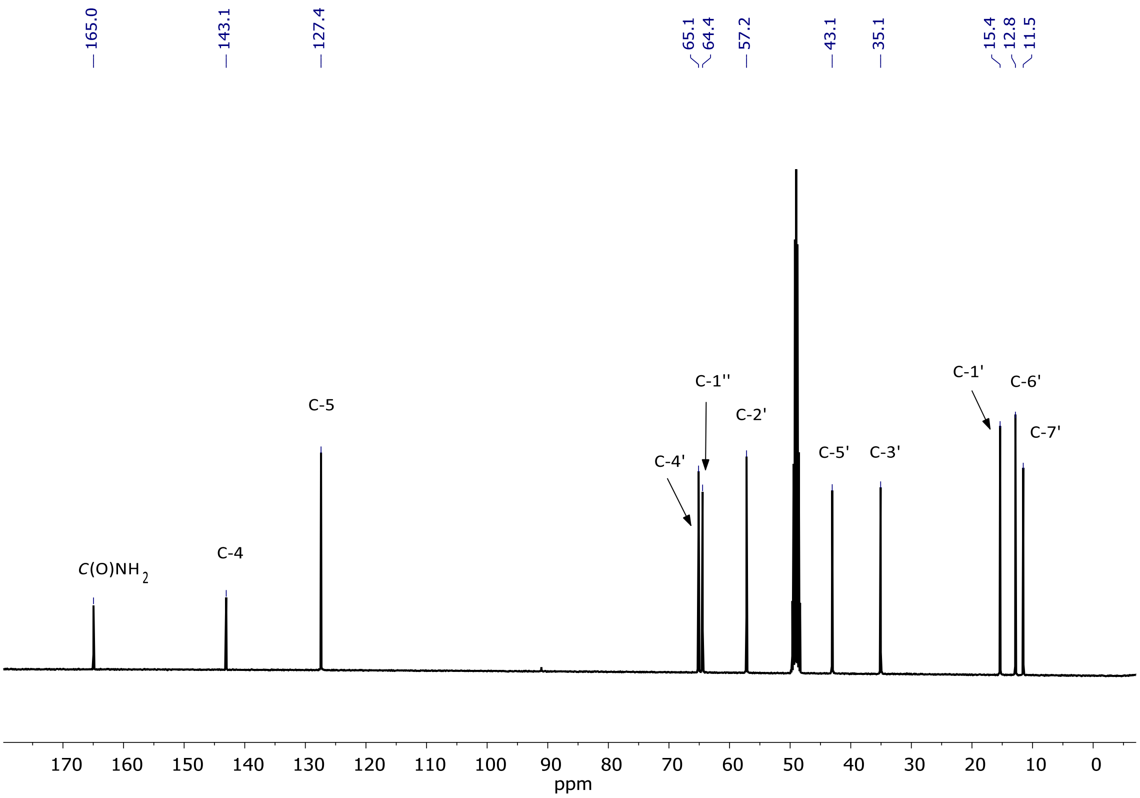

^{13}**C{^{1}H} NMR** (100 MHz, MeOH-d_4)

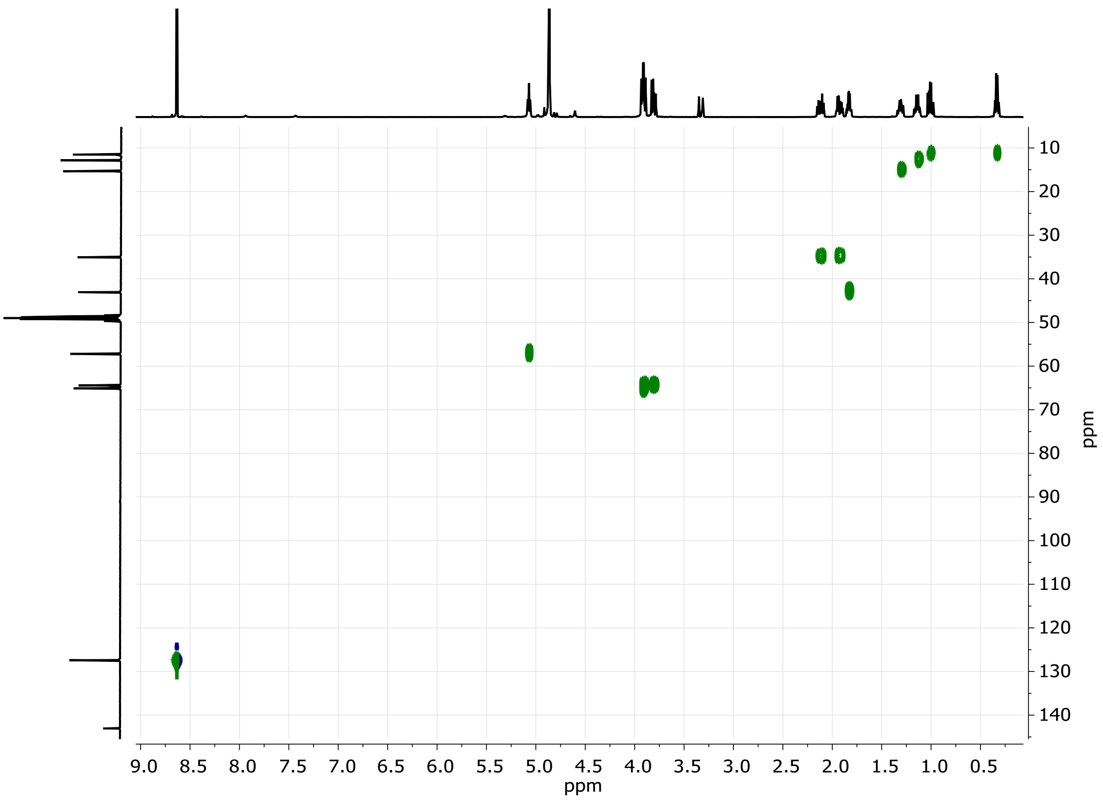

HSQC (400 MHz, MeOH-d_4)

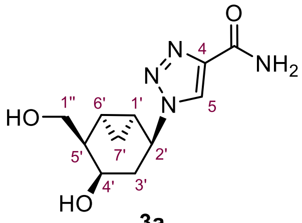

3a

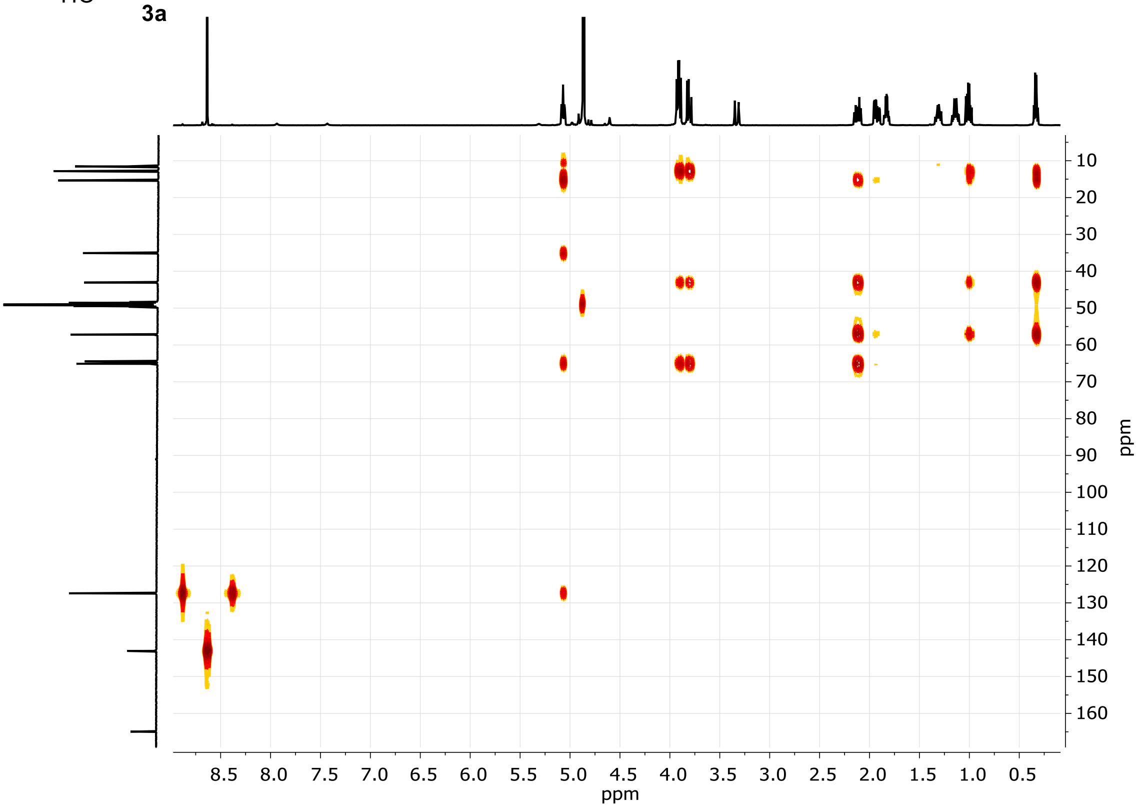

HMBC (400 MHz, MeOH-d_4)

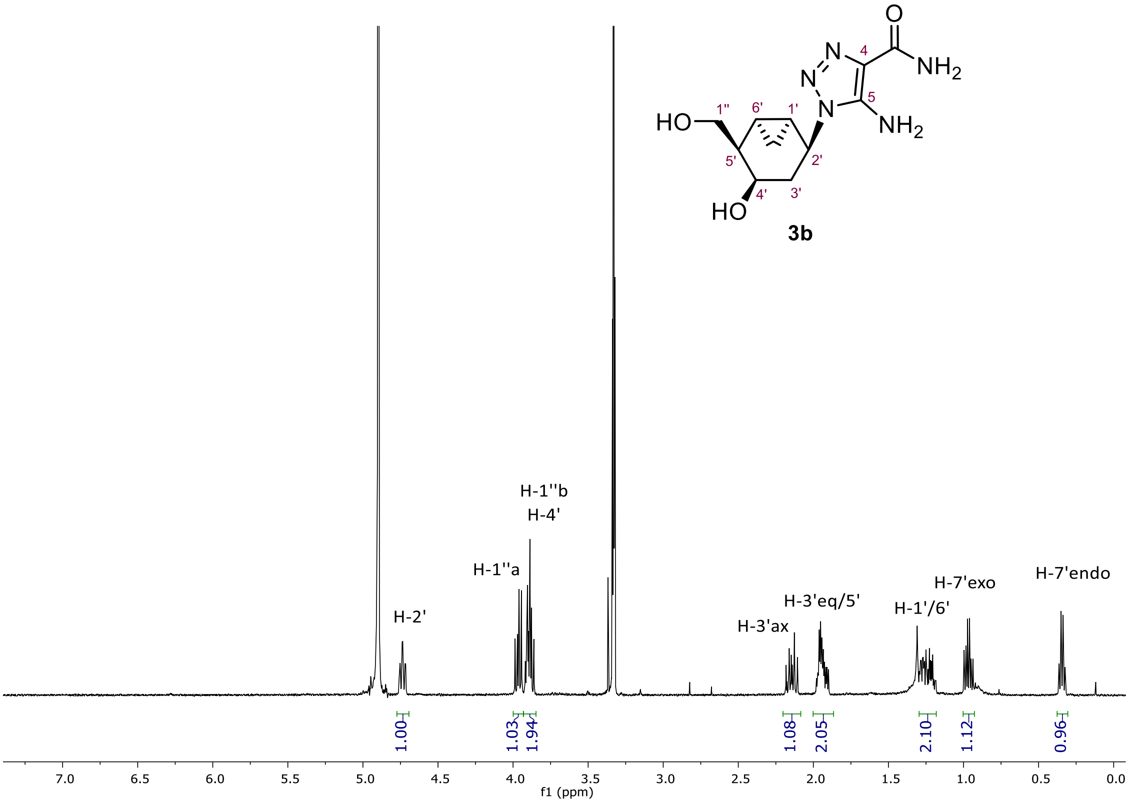

^{1}H NMR (400 MHz, MeOH-d_4)

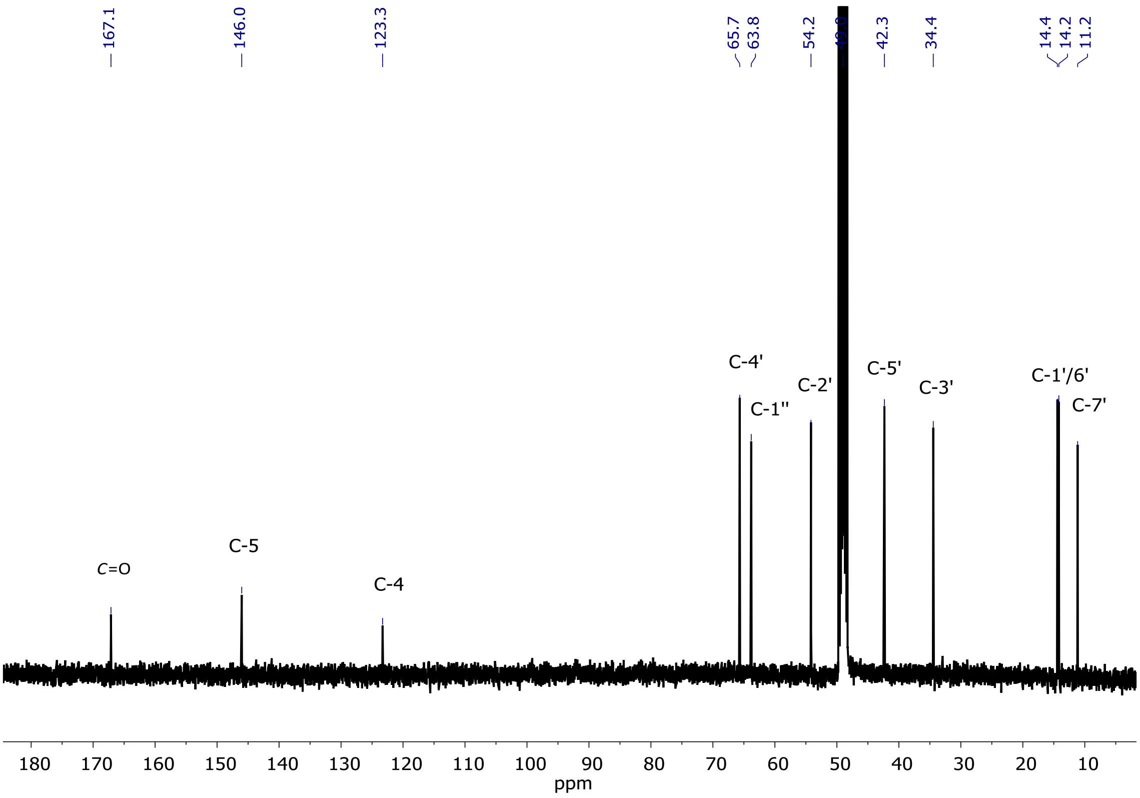

^{13}C{^{1}H} NMR (100 MHz, MeOH-d_4)

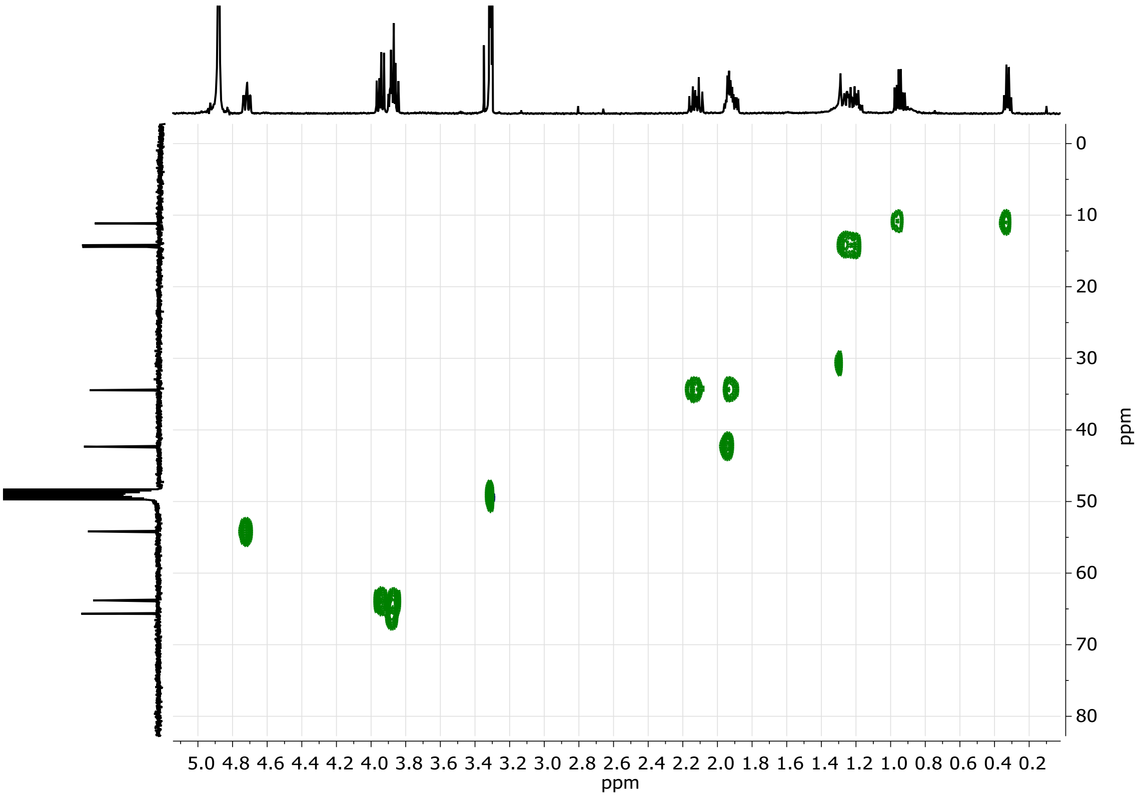

HSQC (400 MHz, MeOH-d_4)

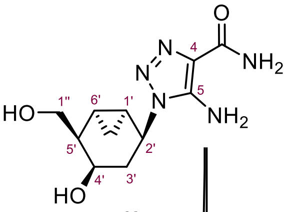

3b

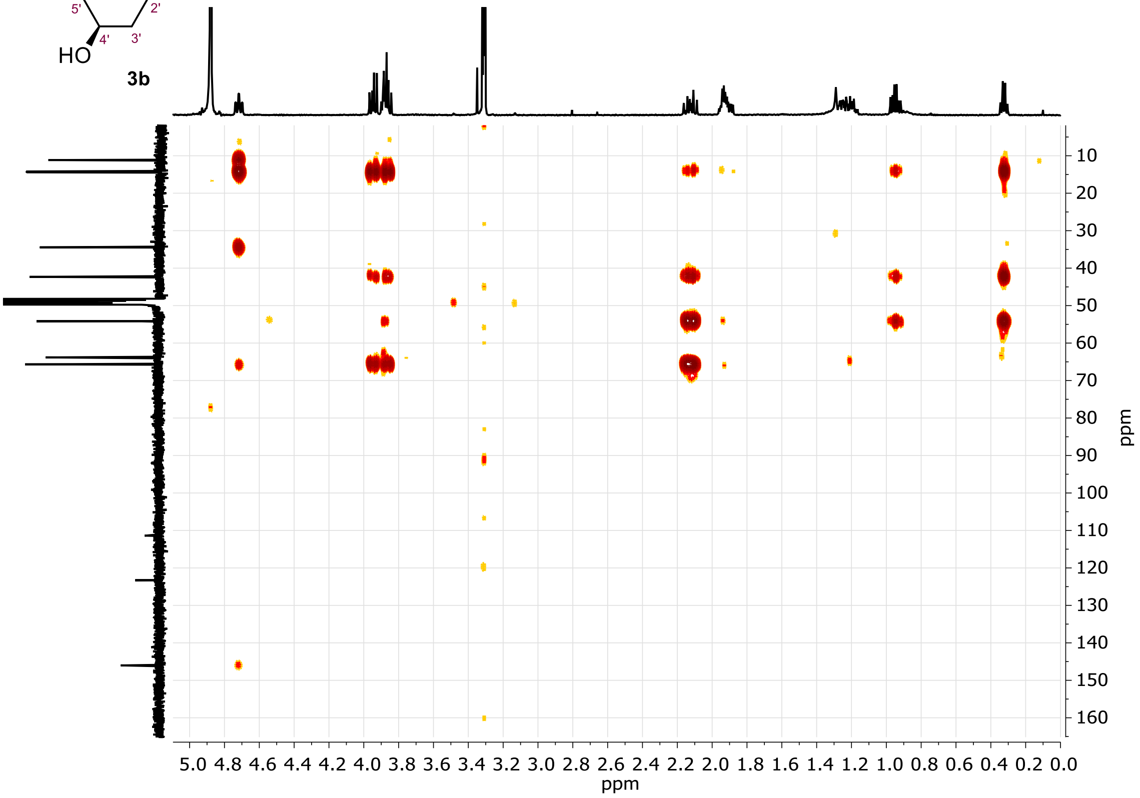

HMBC (400 MHz, MeOH-d_4)

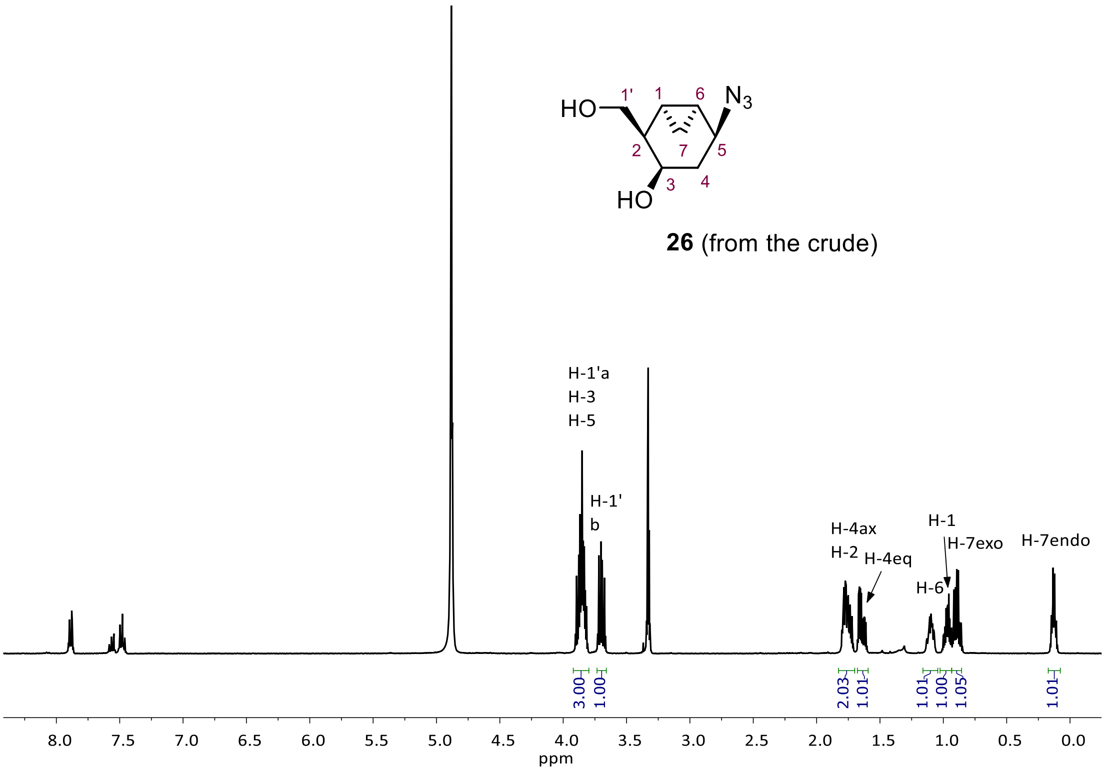

^{1}H NMR (400 MHz, MeOH-d_4)

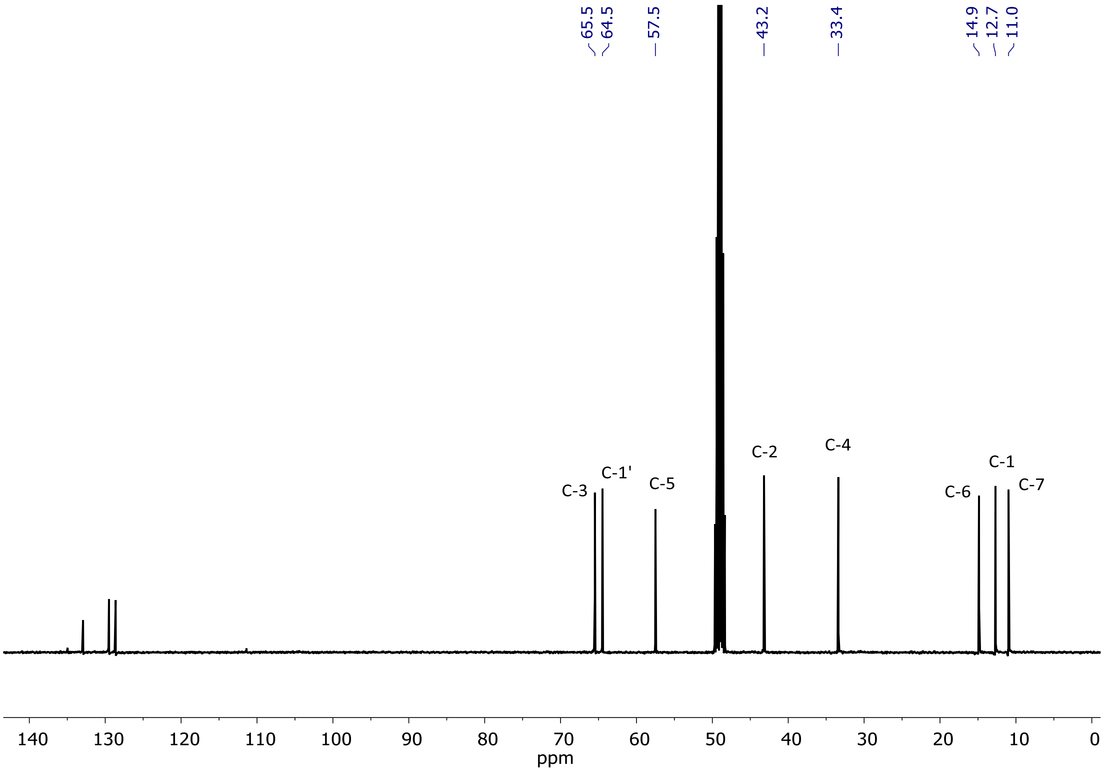

^{13}C{^{1}H} NMR (100 MHz MeOH-d_4)

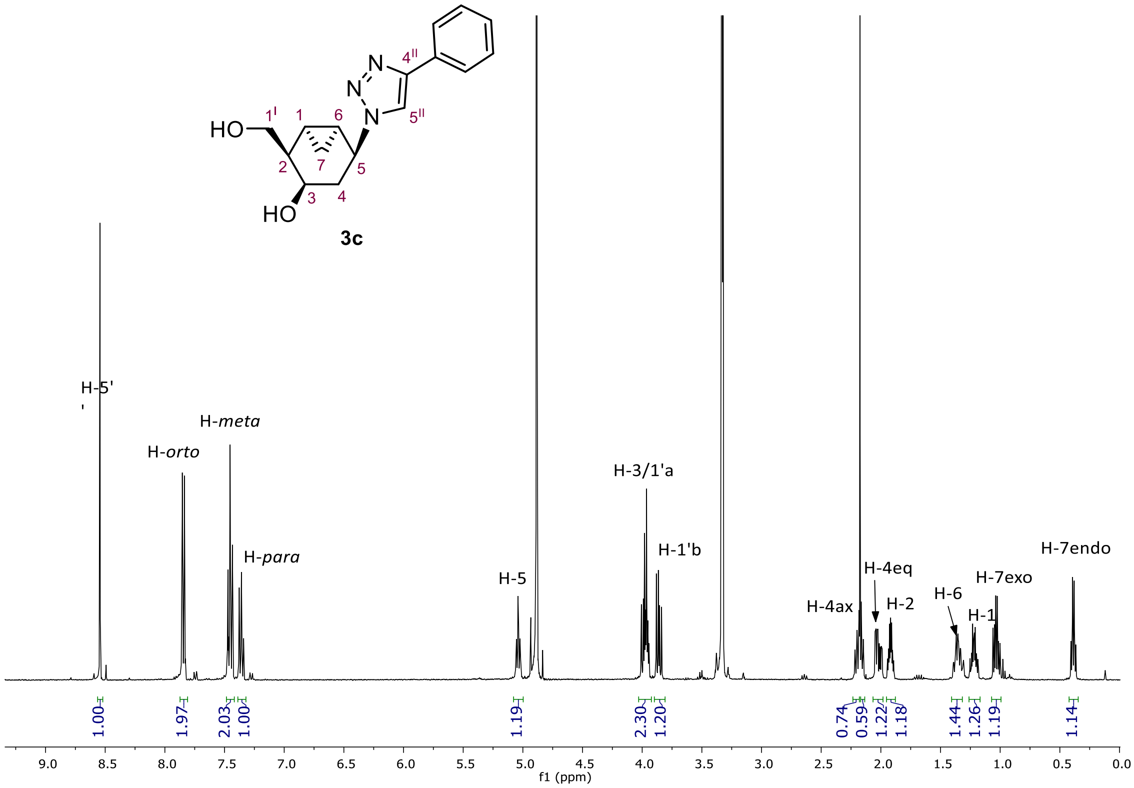

^{1}H NMR (400 MHz, MeOH-d_4)

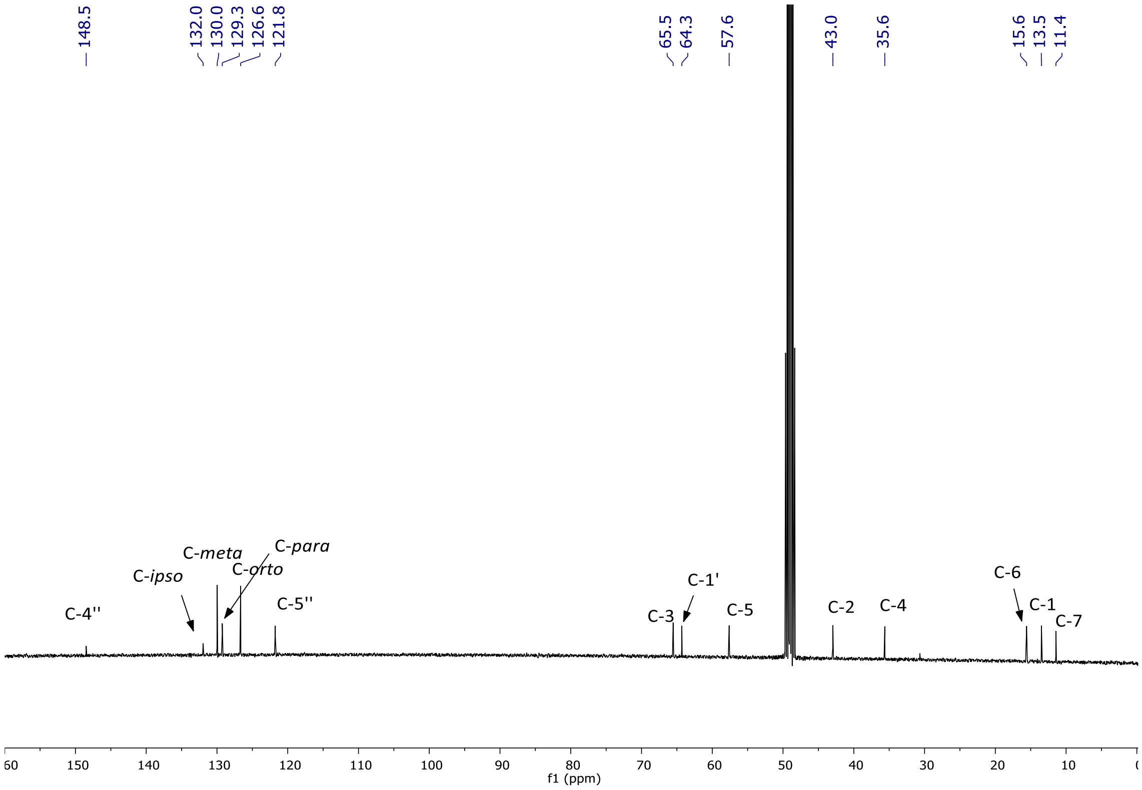

^{13}C{^{1}H} NMR (100 MHz, MeOH-d_4)

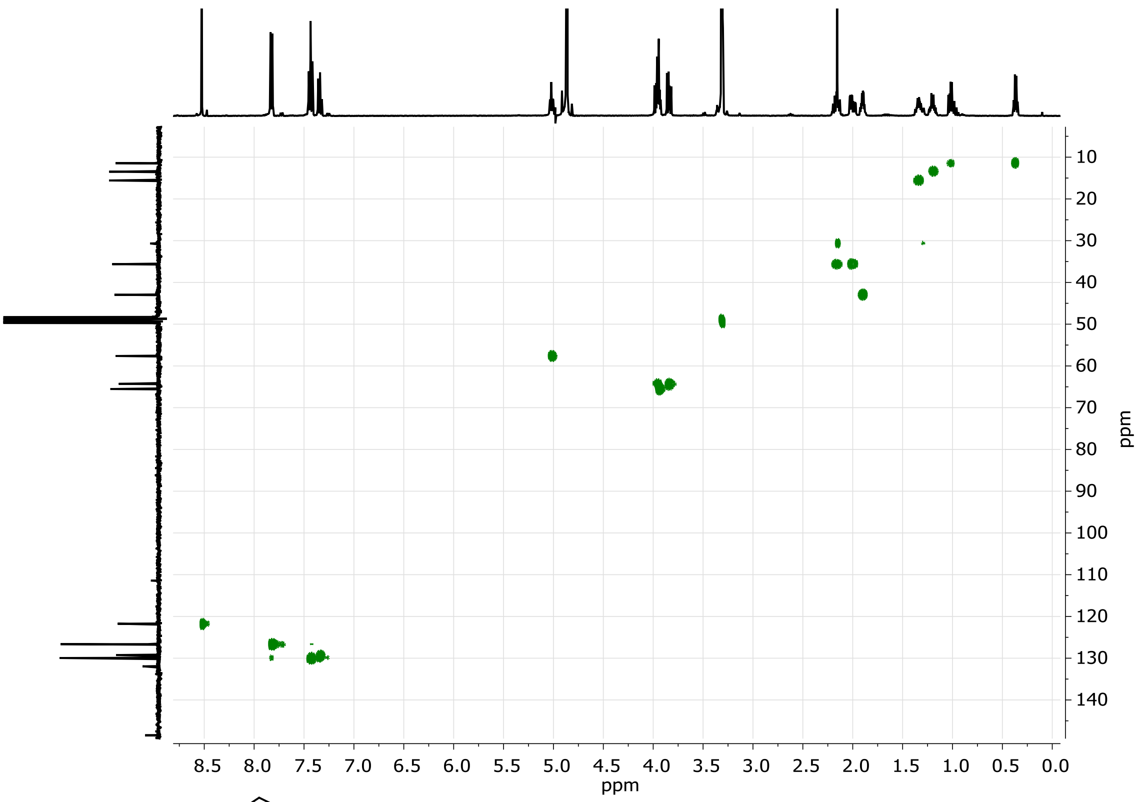

HSQC (400 MHz, MeOH-d_4)

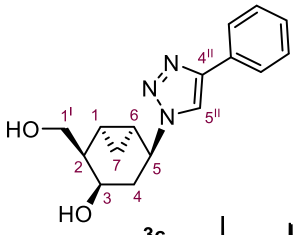

3c

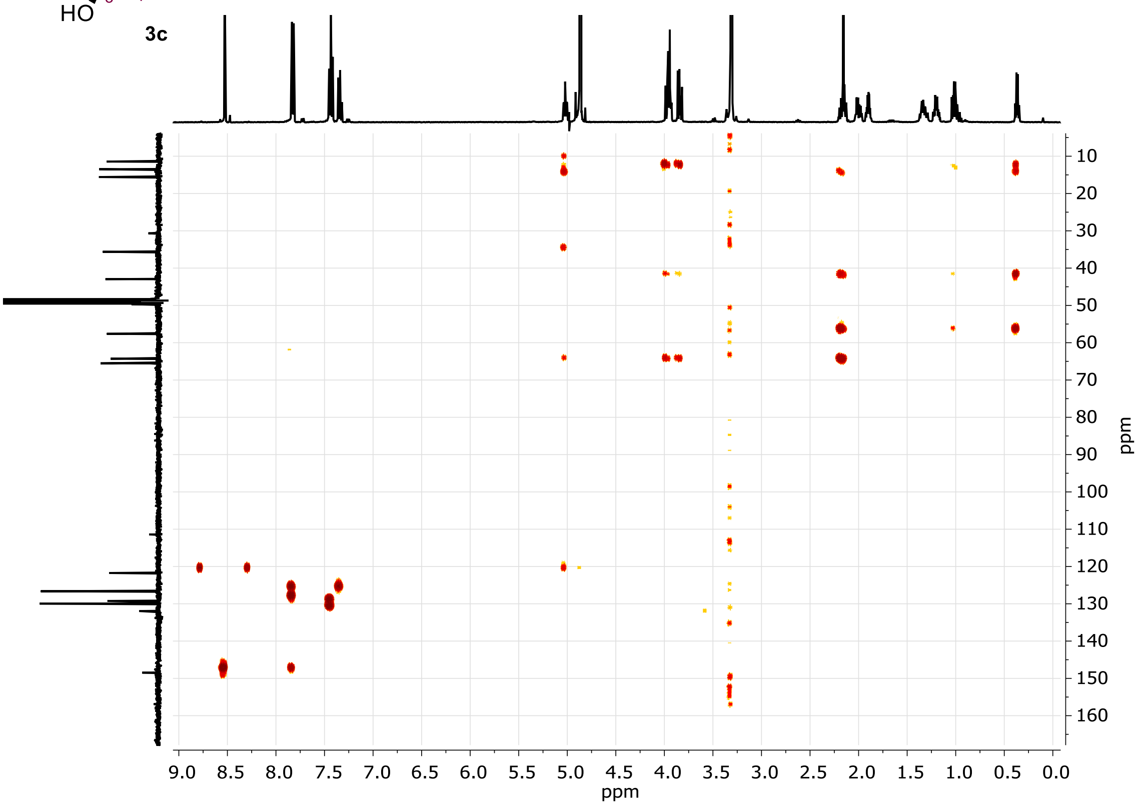

HMBC (400 MHz, MeOH-d_4)

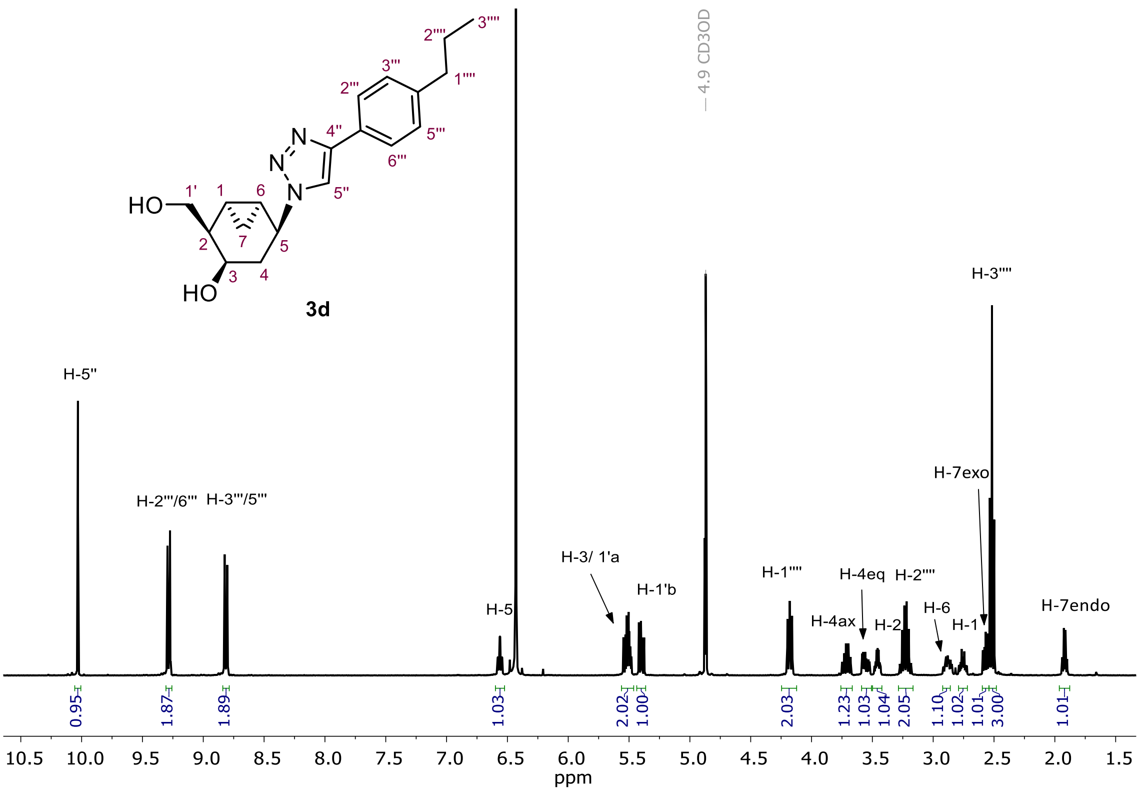

^{1}H NMR (400 MHz, MeOH-d_4)

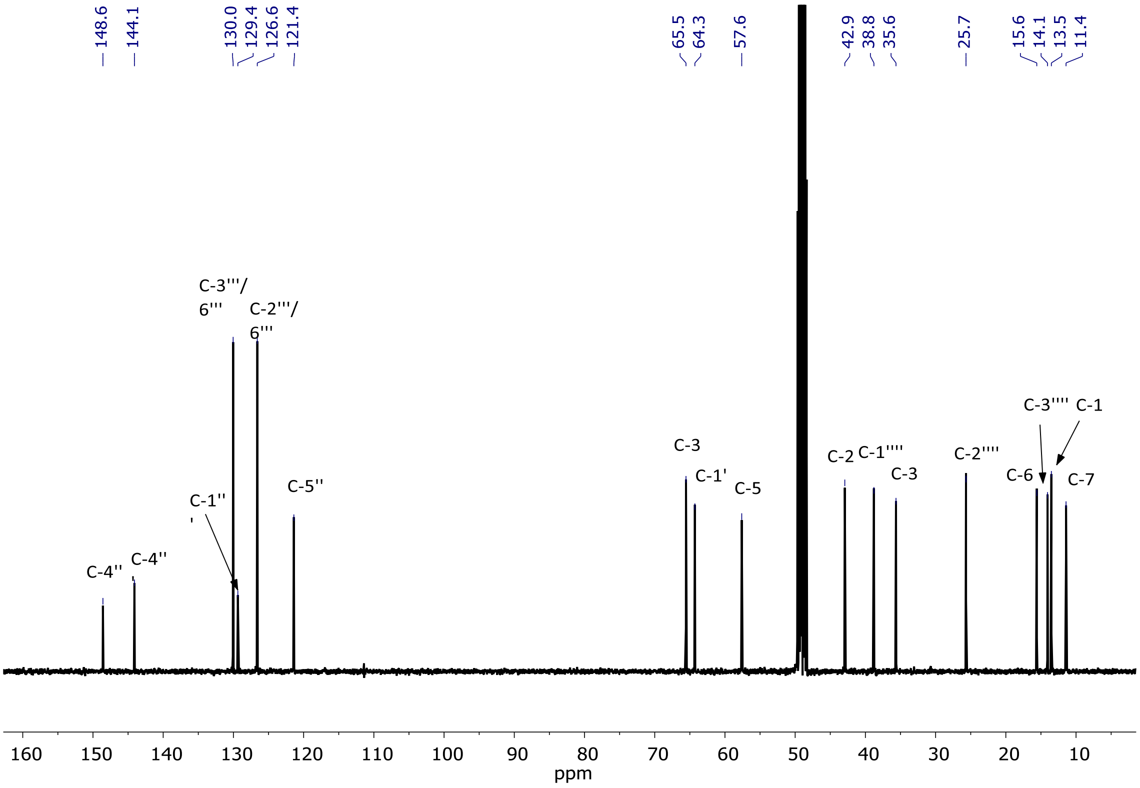

^{13}C{^{1}H} NMR (100 MHz, MeOH-d_4)

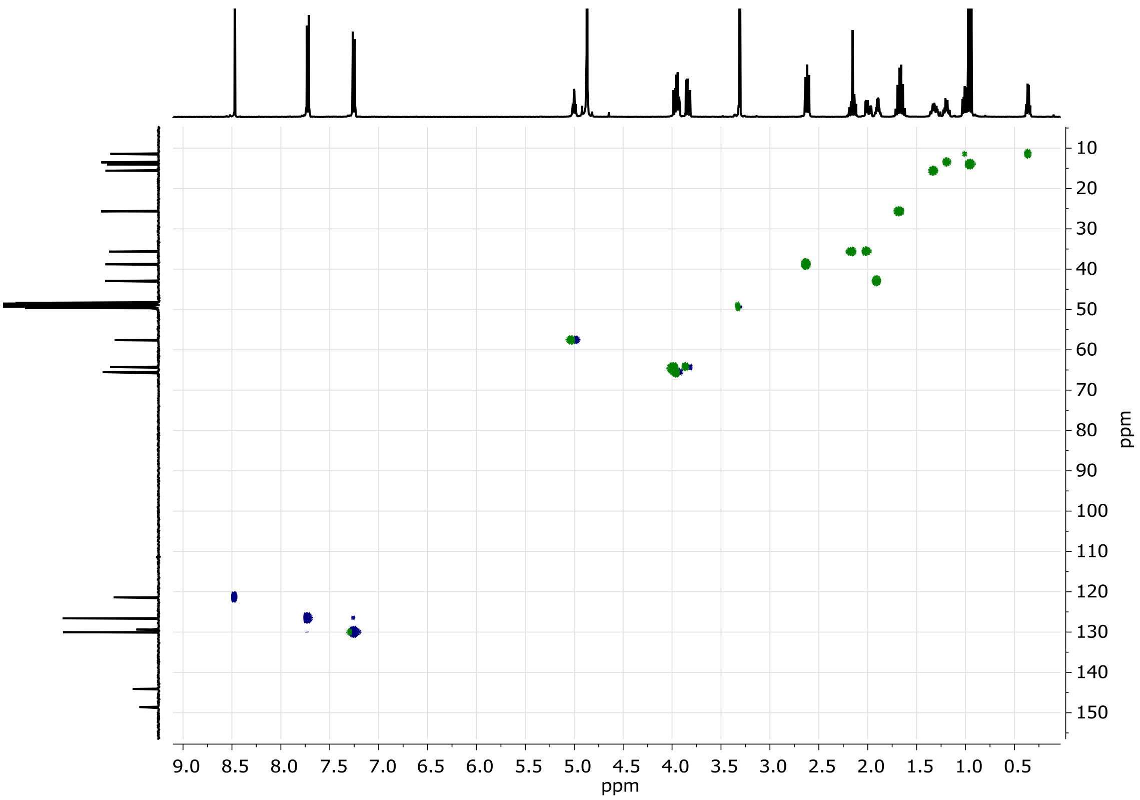

HSQC (400 MHz, MeOH-d_4)

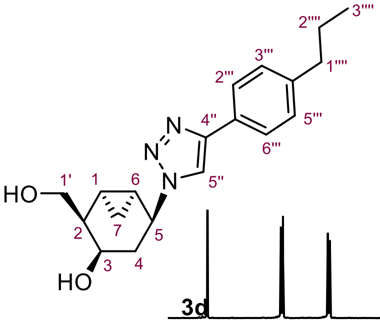

3d

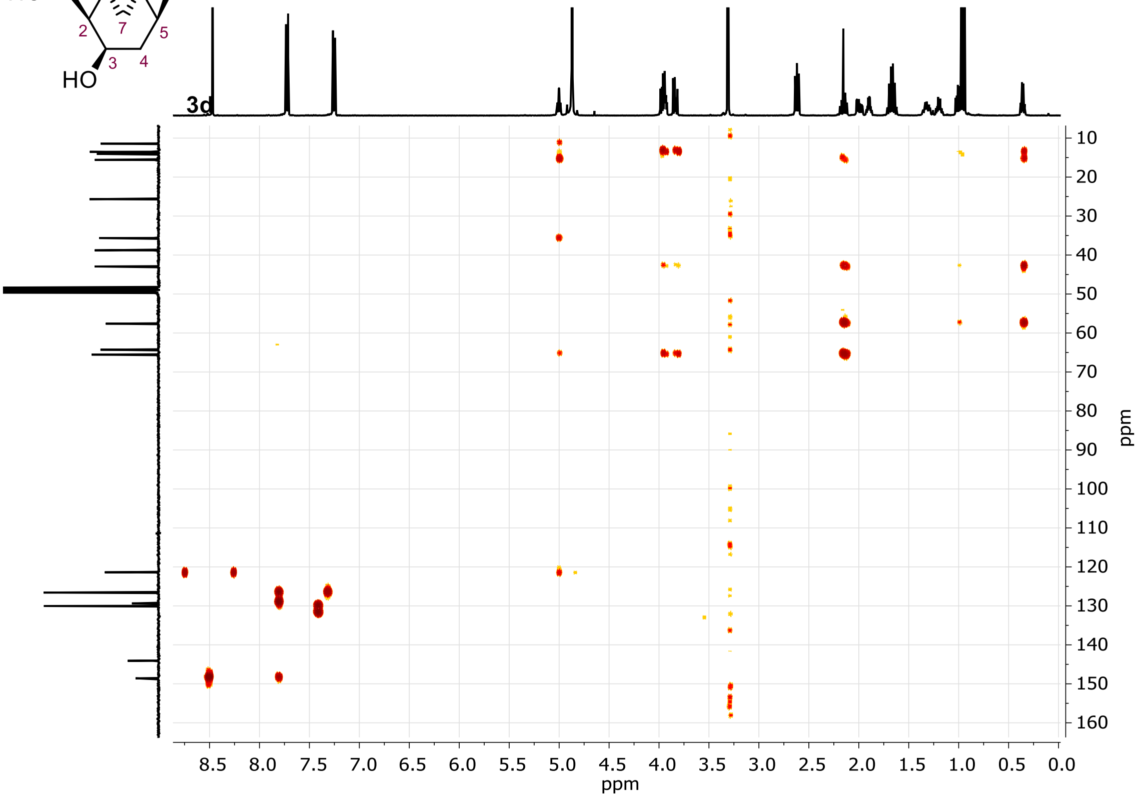

HMBC (400 MHz, MeOH-d_4)

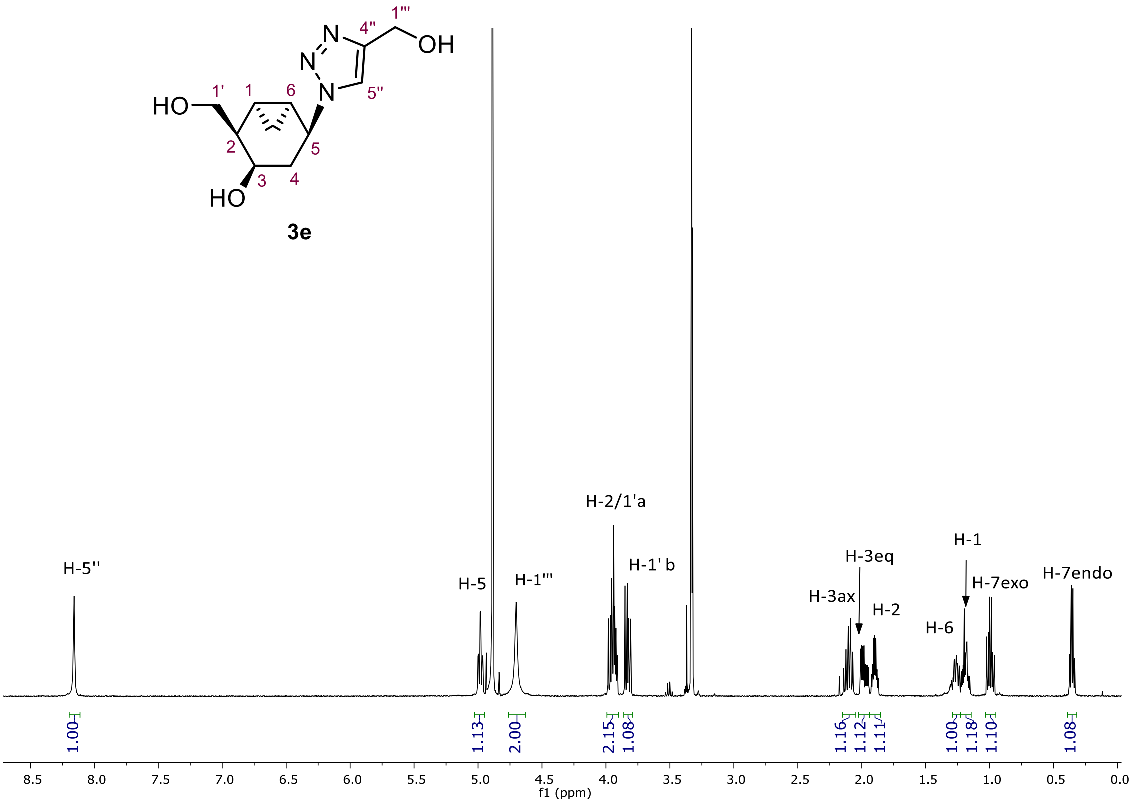

^{1}H NMR (400 MHz, MeOH-d_4)

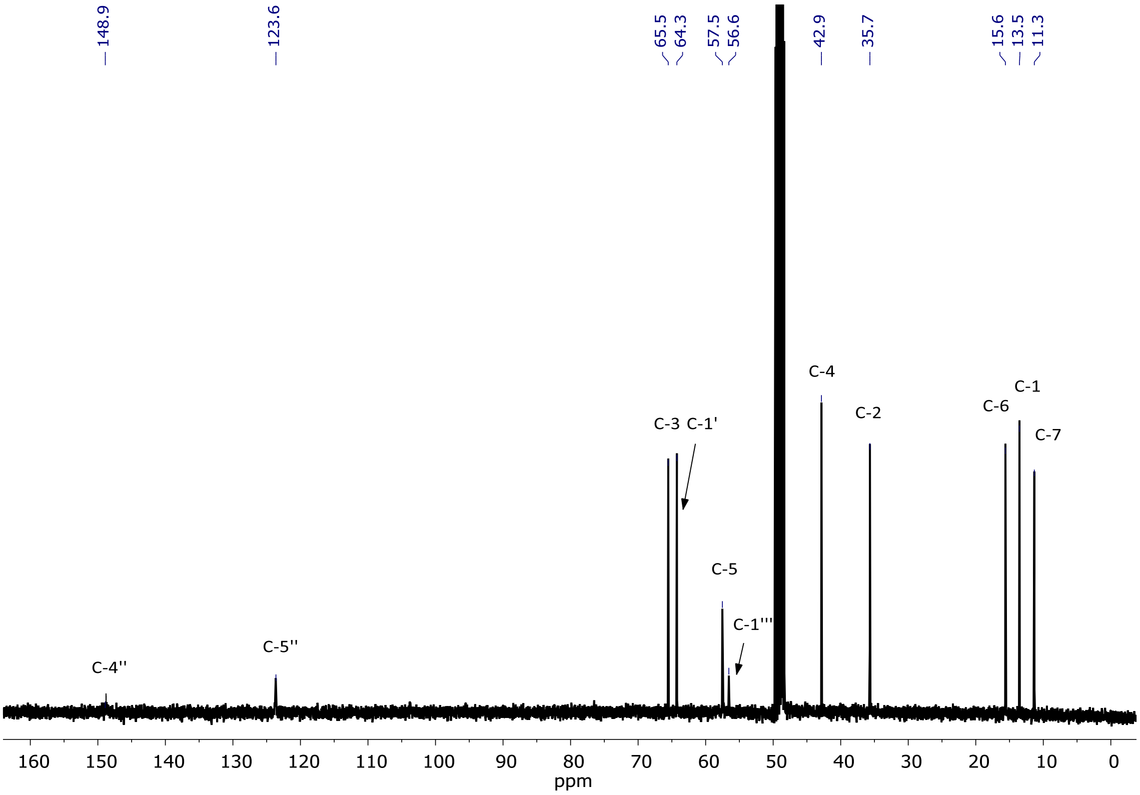

^{13}C{^{1}H} NMR (100 MHz, MeOH-d_4)

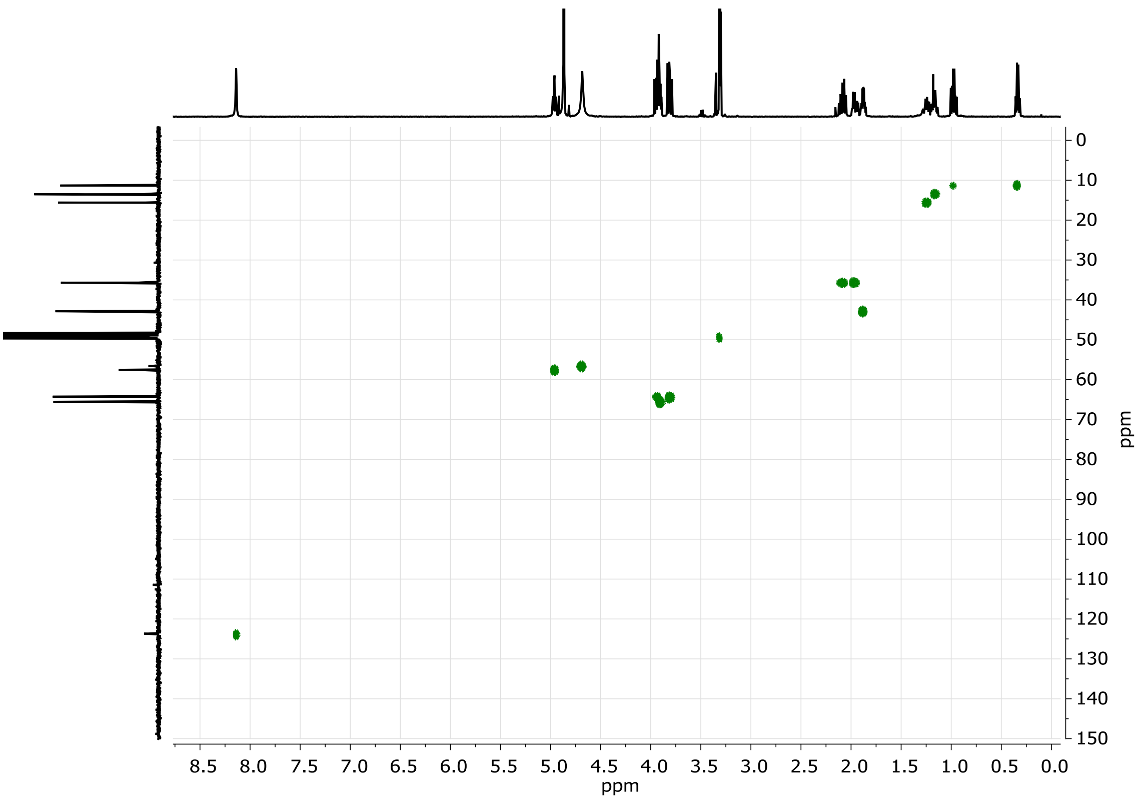

HSQC (400 MHz, MeOH-d_4)

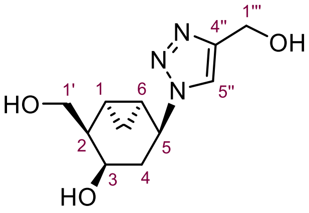

3e

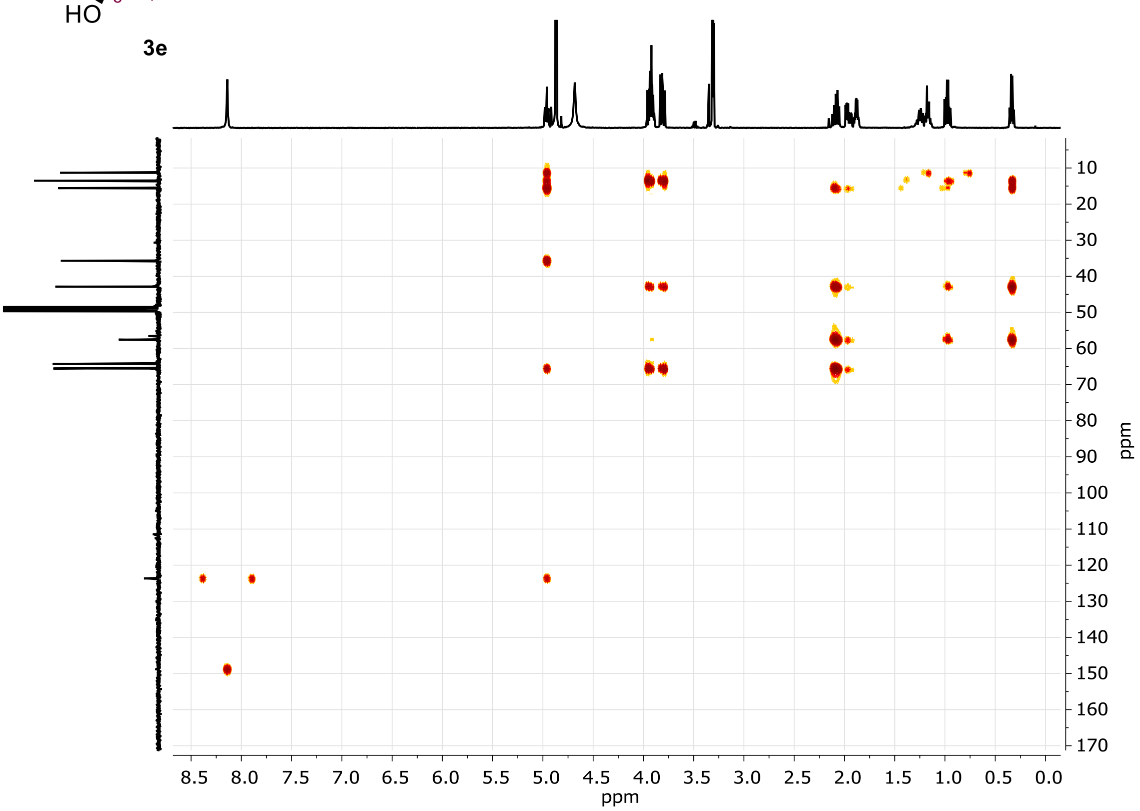

HMBC (400 MHz, MeOH-d_4)

EVALUATION OF CYTOTOXICITY AND ANTIVIRAL ACTIVITY

Nucleosides analogues **1a**, **1b**, **2**, **19** and **3a-e** (Figure S1), have been tested for the cytotoxicity and antiviral activity against different viruses (Tables S1-S5). The cytotoxicity and antiviral activity data against different viruses of the nucleoside analogues **1'a** and **1'b** (Figure S2) bearing a bicyclo[4.1.0]heptane moiety previously synthesized by our group[1] are also included in Tables S1-S4.

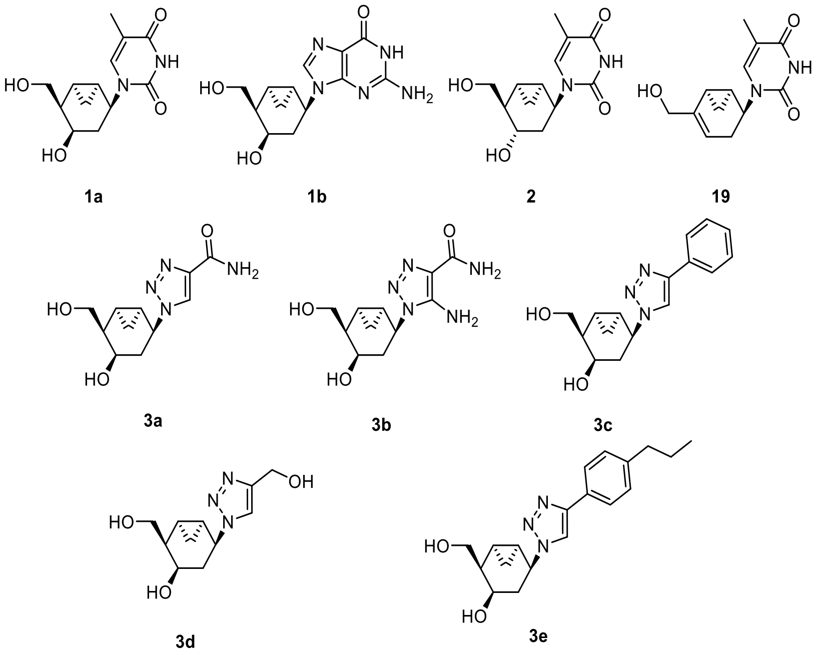

Figure S1. Synthesised prodrug candidates evaluated against different virus.

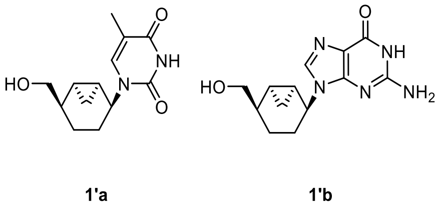

Figure S2. Related CNAs with a bicyclo[4.1.0]heptane scaffold.[1]

1) Domínguez-Pérez, B.; Ferrer, E.; Figueredo, M.; Maréchal, J. D.; Balzarini, J.; Alibés, R.; Busqué, F. Synthesis of Novel Nucleoside Analogues Built on a Bicyclo[4.1.0]heptane Scaffold. *J. Org. Chem.* **2015**, *80*, 9495-9505.

Table S1: Cytotoxicity and antiviral activity of compounds **1a**, **1b**, **2**, **19** and **3a-e** against Herpes simplex virus-1, HSV-1 (KOS), Herpes simplex virus-2, HSV-2 (G), Vaccinia virus, Adeno virus-2 and Human Coronavirus (229E) in human erythroleukemia (HEL) cell cultures.

**Compound**	**Conc. Unit**	**Cytotoxixity CC_{50}[a]**	**Antiviral EC_{50}[b]**				
			Herpes simplex virus-1 (KOS)	Herpes simplex virus-2 (G)	Vaccinia virus	Adeno virus-2	Human Coronavirus (229E)
**1a**	µg/mL	>50	>50	>50	>50	>50	>50
**1b**	µg/mL	>50	>50	>50	>50	>50	>50
**2**	µg/mL	>50	>50	>50	>50	>50	>50
**19**	µg/mL	>50	>50	>50	>50	>50	>50
**3a**	µg/mL	>50	>50	>50	>50	>50	>50
**3b**	µg/mL	>50	>50	>50	>50	>50	>50
**3c**	µg/mL	>50	>50	>50	>50	>50	>50
**3d**	µg/mL	>50	>50	>50	>50	>50	>50
**3e**	µg/mL	>50	>50	>50	>50	>50	>50
**1'a**	µg/mL	>100	>100	>100	>100	>100	--
**1'b**	µg/mL	>100	>100	>100	>100	>100	--
Brivudin	µM	>250	0.2	22.1	12.6	-	-
Cidofovir	µM	>250	39.3	7.5	8.7	9.9	-
Acyclovir	µM	>250	1.9	0.5	>250	-	-
Ganciclovir	µM	>100	0.4	0.09	>100	-	-
Zalcitabine	µM	-	-	-	-	12.0	-
Alovudine	µM	-	-	-	-	0.4	-
UDA	µg/mL	-	-	-	-	-	1.2

[a] 50% Cytotoxic concentration, as determined by measuring the cell viability with the colorimetric formazan-based MTS assay.

[b] 50% Effective concentration, or concentration producing 50% inhibition of virus-induced cytopathic effect, as determined by measuring the cell viability with the colorimetric formazan-based MTS assay.

Table S2. Cytotoxicity and antiviral activity of compounds **1a**, **1b**, **2 19** and **3a-d** against Vesicular stomatitis virus, Coxsackie virus B4, and Respiratory syncytial virus in HEp-2 (Human Epidermoid carcicoma) cell cultures.

Compound	Conc. Unit	Cytotoxixity CC_{50}[a]	Antiviral EC_{50}[b]		
			Vesicular stomatitis virus	Coxsackie virus B4	Respiratory syncytial virus
**1a**	µg/mL	>50	>50	>50	>50
**1b**	µg/mL	>50	>50	>50	>50
**2**	µg/mL	>50	>50	>50	>50
**19**	µg/mL	>50	>50	>50	>50
**3a**	µg/mL	>50	>50	>50	>50
**3b**	µg/mL	>50	>50	>50	>50
**3c**	µg/mL	>50	>50	>50	>50
**3d**	µg/mL	17.8	>50	>50	>50
**3e**	µg/mL	>50	>50	>50	>50
**1'a**	µg/mL	>100	>100	>100	>100
**1'b**	µg/mL	>100	>100	>100	>100
DS-10,000	µg/mL	>100	0.08	2.5	0.01
Ribavirin	µM	>250	149	117	6.7

[a] 50% Cytotoxic concentration, as determined by measuring the cell viability with the colorimetric formazan-based MTS assay.

[b] 50% Effective concentration, or concentration producing 50% inhibition of virus-induced cytopathic effect, as determined by measuring the cell viability with the colorimetric formazan-based MTS assay.

Table S3. Cytotoxicity and antiviral activity of compounds **1a**, **1b**, **2**, **19** and **3a-3e** against Reovirus-1, Sindbis virus, Coxsackie virus B4, Punta Toro virus, Yellow Fever virus and Zika virus in Vero cell (kidney epithelial cells from African green monkey) cultures.

**Compound**	**Conc. Unit**	**Cytotoxicity CC_{50}[a]**	**Antiviral EC_{50}[b]**					
			Reo-virus-1	Sindbis virus	Coxsackie virus B4	Punta Toro virus	Yellow Fever virus	Zika virus
**1a**	µg/mL	>50	>50	>50	>50	>50	>50	>50
**1b**	µg/mL	>50	>50	>50	>50	>50	>50	>50
**2**	µg/mL	>50	>50	>50	>50	>50	>50	>50
**19**	µg/mL	>50	>50	>50	>50	>50	>50	>50
**3a**	µg/mL	>50	>50	>50	>50	>50	>50	>50
**3b**	µg/mL	>50	>50	>50	>50	>50	>50	>50
**3c**	µg/mL	>50	>50	>50	>50	>50	>50	>50
**3d**	µg/mL	>50	>50	>50	**13.5**	>50	>50	>50
**3d**	µg/mL	78.8	>100	>100	**9.4**	>100	>100	>100
**3e**	µg/mL	>50	>50	>50	>50	>50	>50	>50
**1'a**	µg/mL	>100	>100	>100	>100	>100	--	--
**1'b**	µg/mL	>100	>100	>100	>100	>100	--	--
Ds-10,000	µg/mL	>100	>100	14.9	3.8	1.3	6.9	3.4
Mycophenolic acid	µM	>100	7.1	3.6	>100	>100	2.6	0.2

[a] 50% Cytotoxic concentration, as determined by measuring the cell viability with the colorimetric formazan-based MTS assay.

[b] 50% Effective concentration, or concentration producing 50% inhibition of virus-induced cytopathic effect, as determined by measuring the cell viability with the colorimetric formazan-based MTS assay.

Table S4. Cytotoxicity and antiviral activity of compounds **1a**, **1b**, **2**, **19** and **3a-3e** against Influenza A virus A/Ned/378/05A (H1N1), Influenza A virus A/HK/7/87 (H3N2) and Influenza B virus B/Ned/537/05 in MDCK (Madin-Darby Canine Kidney) cell cultures.

**Compound**	**Conc. Unit**	**Cytotoxixity CC_{50}[a]**	**Antiviral EC_{50}[b]**		
			Influenza A virus A/Ned/378/05 (H1N1)	Influenza A virus A/HK/7/87 (H3N2)	Influenza B virus B/Ned/537/05
**1a**	µg/mL	>50	>50	>50	>50
**1b**	µg/mL	>50	>50	>50	>50
**2**	µg/mL	>50	>50	>50	>50
**19**	µg/mL	18.6	>50	>50	>50
**3a**	µg/mL	>50	>50	>50	>50
**3b**	µg/mL	>50	>50	>50	>50
**3c**	µg/mL	20.4	>50	>50	>50
**3d**	µg/mL	10.6	>50	>50	>50
**3e**	µg/mL	>50	>50	>50	>50
**1'a**	µg/mL	>100	>100	>100	>100
**1'b**	µg/mL	>100	>100	>100	>100
Zanamivir	µM	>100	0.9	4.9	1.4
Ribavirin	µM	>100	7.4	6.4	9.3
Rimantadine	µM	>200	88.8	0.06	>200

[a] 50% Cytotoxic concentration, as determined by measuring the cell viability with the colorimetric formazan-based MTS assay.
[b] 50% Effective concentration, or concentration producing 50% inhibition of virus-induced cytopathic effect, as determined by measuring the cell viability with the colorimetric formazan-based MTS assay.

Table S5. Cytotoxicity and antiviral activity of compounds **1a**, **1b**, **2**, **19** and **3a-e** against Human immunodeficiency virus (III_B), and Human immunodeficiency virus (ROD) in MT-4 cell cultures (human lymphocytic cell line).

**Compound**	**Conc. Unit**	**Cytotoxixity CC_{50}[a]**	**Antiviral EC_{50}[b]**	
			Human immunodeficiency virus (III_B)	Human immunodeficiency virus (ROD)
**1a**	µg/mL	99.07	>99.07	>99.07
**1b**	µg/mL	>125	>125	>125
**2**	µg/mL	>125	>125	>125
**19**	µg/mL	109.2	>109.2	>109.2
**3a**	µg/mL	>125	>125	>125
**3b**	µg/mL	>125	>125	>125
**3c**	µg/mL	96.22	>96.22	>96.22
**3d**[c]	µg/mL	9.36	>9.36	>9.36
**3e**	µg/mL	≥115.82	>115.82	>115.82
Neviparine	µg/mL	>4	0.075	>4
Zidovudine	µg/mL	>2	0.002	0.002
Lamivudine	µg/mL	>20	0.58	2.27
Didanosine	µg/mL	>50	17.95	19.40

[a] 50% Cytotoxic concentration, as determined by measuring the cell viability with the colorimetric formazan-based MTS assay.

[b] 50% Effective concentration, or concentration producing 50% inhibition of virus-induced cytopathic effect, as determined by measuring the cell viability with the colorimetric formazan-based MTS assay.

[C] Crystallization observed at 125 µg/mL.

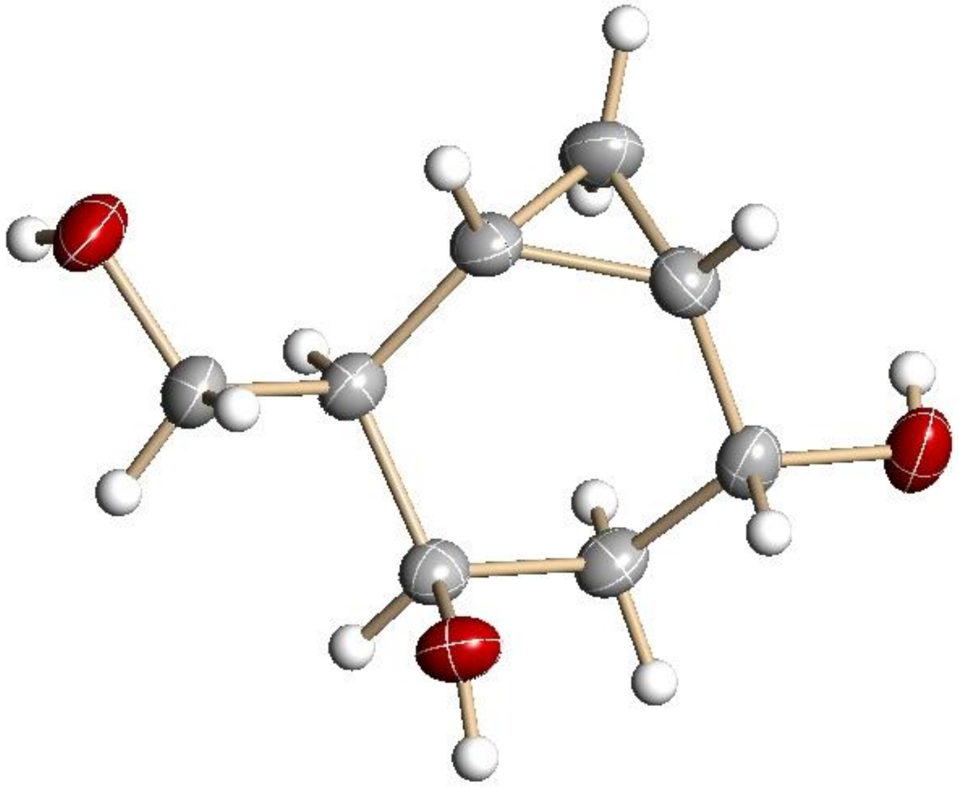

Figure S3. Thermal ellipsoid plot of compound **12**. The ellipsoid contour probability level is 50%.

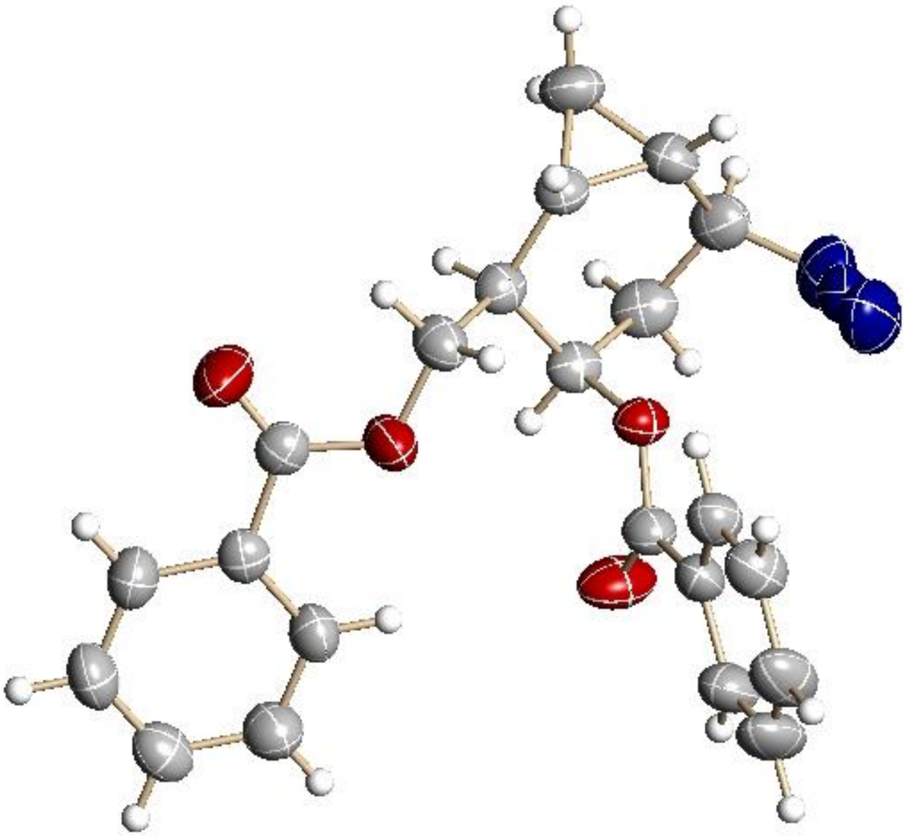

Figure S4. Thermal ellipsoid plot of compound **6**. The ellipsoid contour probability level is 50%.

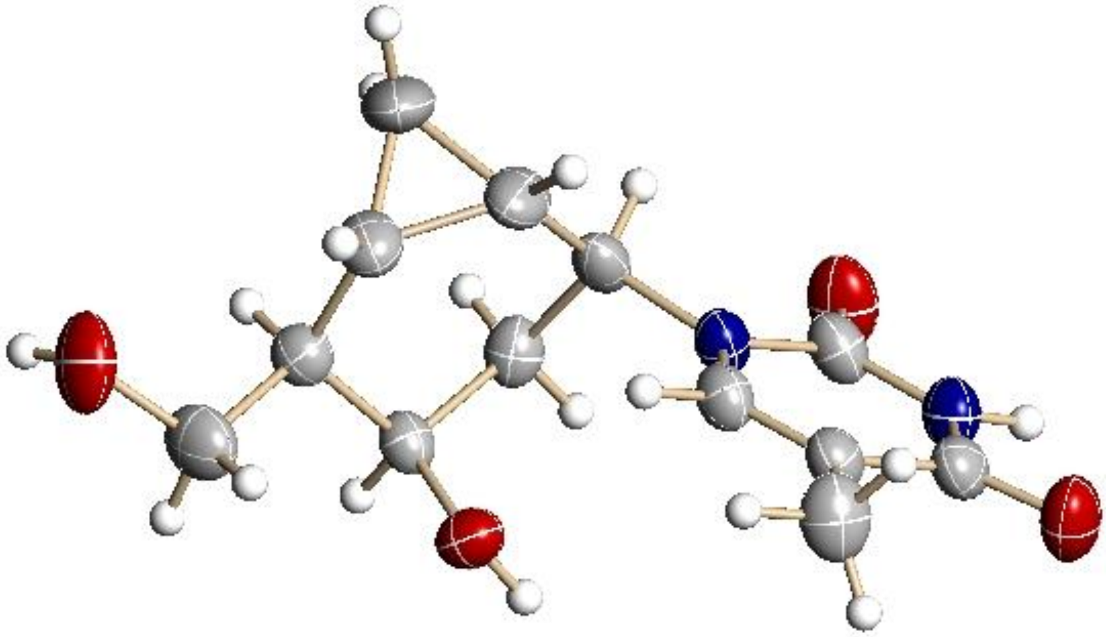

Figure S5. Thermal ellipsoid plot of compound **1a**. The ellipsoid contour probability level is 50%.

X-ray Structure Determination.

Adequate crystals were obtained by crystallization from AcOEt/hexane for compound **6** and from MeO/Et_2O for compounds **12** and **1a.** Data were collected using Mo Kα radiation in a SMART-APEX. An empirical absorption correction was applied (SADABS). Structures were solved by direct methods (SHELXS-86) and refined by full-matrix least-squares methods on F2 for all reflections (SHELXL- 2016). Non-hydrogen atoms were refined anisotropically. Hydrogen atoms bonded to carbon atoms were placed in calculated positions with isotropic displacement parameters fixed at 1.2 times the Ueq of the corresponding carbon atoms. Hydrogen atoms bonded to N or O atoms were found and refined with isotropic displacement parameters fixed at 1.5 times the Ueq of the corresponding heteroatoms.

Crystal data and further refinement details are presented in **Tables S6-S8**.

Table S6. Crystal data and structure refinement for compound **12**.

Empirical formula	$C_8 H_{14} O_3$	
Formula weight	158.19	
Temperature	296(2) K	
Wavelength	0.71073 Å	
Crystal system	Monoclinic	
Space group	$P\ 2_1$	
Unit cell dimensions	a = 6.4586(4) Å	α= 90°.
	b = 9.2744(6) Å	β= 104.515(1)°.
	c = 6.7751(5) Å	γ = 90°.
Volume	392.87(5) $Å^3$	
Z	2	
Density (calculated)	1.337 Mg/m^3	
Absorption coefficient	0.101 mm^{-1}	
F(000)	172	
Crystal size	0.56 x 0.50 x 0.21 mm^3	
Theta range for data collection	3.106 to 28.514°.	
Index ranges	-8<=h<=8, -12<=k<=12, -8<=l<=8	
Reflections collected	3124	
Independent reflections	1802 [R(int) = 0.0090]	
Completeness to theta = 25.000°	99.9 %	
Absorption correction	Semi-empirical from equivalents	
Max. and min. transmission	1 and 0.897	
Refinement method	Full-matrix least-squares on F^2	
Data / restraints / parameters	1802 / 1 / 109	
Goodness-of-fit on F^2	1.048	
Final R indices [I>2sigma(I)]	R1 = 0.0308, wR2 = 0.0813	
R indices (all data)	R1 = 0.0313, wR2 = 0.0819	
Absolute structure parameter	0.0(2)	
Largest diff. peak and hole	0.175 and -0.176 e.$Å^{-3}$	

Table S7. Crystal data and structure refinement for compound **6**.

Empirical formula	$C_{22} H_{21} N_3 O_4$	
Formula weight	391.42	
Temperature	296(2) K	
Wavelength	0.71073 Å	
Crystal system	Orthorhombic	
Space group	$P 2_1 2_1 2_1$	
Unit cell dimensions	a = 7.0431(3) Å	α= 90°.
	b = 10.4201(5) Å	β= 90°.
	c = 26.5539(13) Å	γ = 90°.
Volume	1948.79(16) $Å^3$	
Z	4	
Density (calculated)	1.334 Mg/m^3	
Absorption coefficient	0.093 mm^{-1}	
F(000)	824	
Crystal size	0.72 x 0.29 x 0.22 mm^3	
Theta range for data collection	1.534 to 28.820°.	
Index ranges	-9<=h<=9, -13<=k<=14, -34<=l<=35	
Reflections collected	15592	
Independent reflections	4735 [R(int) = 0.0224]	
Completeness to theta = 25.000°	100.0 %	
Refinement method	Full-matrix least-squares on F^2	
Data / restraints / parameters	4735 / 0 / 262	
Goodness-of-fit on F^2	1.023	
Final R indices [I>2sigma(I)]	R1 = 0.0380, wR2 = 0.0859	
R indices (all data)	R1 = 0.0480, wR2 = 0.0910	
Absolute structure parameter	0.2(3)	
Largest diff. peak and hole	0.142 and -0.158 e.$Å^{-3}$	

Table S8. Crystal data and structure refinement for compound **1a**.

Empirical formula	$C_{13} H_{18} N_2 O_4$	
Formula weight	266.29	
Temperature	296(2) K	
Wavelength	0.71073 Å	
Crystal system	Orthorhombic	
Space group	$P\ 2_1\ 2_1\ 2_1$	
Unit cell dimensions	a = 7.0327(6) Å	α= 90°.
	b = 9.9275(9) Å	β= 90°.
	c = 18.7647(17) Å	γ = 90°.
Volume	1310.1(2) $Å^3$	
Z	4	
Density (calculated)	1.350 Mg/m^3	
Absorption coefficient	0.101 mm^{-1}	
F(000)	568	
Crystal size	0.18 x 0.13 x 0.03 mm^3	
Theta range for data collection	2.171 to 28.765°.	
Index ranges	-9<=h<=9, -12<=k<=13, -24<=l<=23	
Reflections collected	10580	
Independent reflections	3156 [R(int) = 0.0808]	
Completeness to theta = 25.000°	99.3 %	
Absorption correction	Semi-empirical from equivalents	
Max. and min. transmission	1 and 0.860	
Refinement method	Full-matrix least-squares on F^2	
Data / restraints / parameters	3156 / 0 / 182	
Goodness-of-fit on F^2	1.026	
Final R indices [I>2sigma(I)]	R1 = 0.0709, wR2 = 0.1028	
R indices (all data)	R1 = 0.1352, wR2 = 0.1173	
Absolute structure parameter	-0.2(9)	
Largest diff. peak and hole	0.150 and -0.178 e.$Å^{-3}$	
